# Global burden of atrial fibrillation/atrial flutter and its attributable risk factors from 1990 to 2021

**DOI:** 10.1093/europace/euae195

**Published:** 2024-07-10

**Authors:** Siyuan Cheng, JinZheng He, Yuchen Han, Shaojie Han, Panpan Li, Huanyan Liao, Jun Guo

**Affiliations:** Department of Cardiology, The First Affiliated Hospital of Jinan University, No. 613, Huangpu Avenue West, Tianhe District, Guangzhou, Guangdong 510630, China; Department of Cardiology, The First Affiliated Hospital of Jinan University, No. 613, Huangpu Avenue West, Tianhe District, Guangzhou, Guangdong 510630, China; Department of Cardiology, The First Affiliated Hospital of Jinan University, No. 613, Huangpu Avenue West, Tianhe District, Guangzhou, Guangdong 510630, China; Department of Cardiology, The First Affiliated Hospital of Jinan University, No. 613, Huangpu Avenue West, Tianhe District, Guangzhou, Guangdong 510630, China; Department of Cardiology, The First Affiliated Hospital of Jinan University, No. 613, Huangpu Avenue West, Tianhe District, Guangzhou, Guangdong 510630, China; Department of Cardiology, The First Affiliated Hospital of Jinan University, No. 613, Huangpu Avenue West, Tianhe District, Guangzhou, Guangdong 510630, China; Department of Cardiology, The First Affiliated Hospital of Jinan University, No. 613, Huangpu Avenue West, Tianhe District, Guangzhou, Guangdong 510630, China

**Keywords:** Atrial fibrillation/flutter, Global Burden of Disease, Incidence, Deaths, Disability-adjusted life years, Risk factors

## Abstract

**Aims:**

To devise effective preventive measures, a profound understanding of the evolving patterns and trends in atrial fibrillation (AF) and atrial flutter (AFL) burdens is pivotal. Our study was designed to quantify the burden and delineate the risk factors associated with AF and AFL across 204 countries and territories spanning 1990–2021.

**Methods and results:**

Data pertaining to AF and AFL were sourced from the Global Burden of Disease Study 2021. The burden of AF/AFL was evaluated using metrics such as incidence, disability-adjusted life years (DALYs), deaths, and their corresponding age-standardized rates (ASRs), stratified by age, sex, socio-demographic index (SDI), and human development index (HDI). The estimated annual percentage change was employed to quantify changes in ASRs. Population attributable fractions were calculated to determine the proportional contributions of major risk factors to age-standardized AF/AFL deaths. This analysis encompassed the period from 1990 to 2021. Globally, in 2021, there were 4.48 million incident cases [95% uncertainty interval (UI): 3.61–5.70], 8.36 million DALYs (95% UI: 6.97–10.13) and 0.34 million deaths (95% UI: 0.29–0.37) attributed to AF/AFL. The AF/AFL burden in 2021, as well as its trends from 1990 to 2021, displayed substantial variations based on gender, SDI quintiles, and geographical regions. High systolic blood pressure emerged as the leading contributor to age-standardized AF/AFL incidence, prevalence, death, and DALY rate globally among all potential risk factors, followed closely by high body mass index.

**Conclusion:**

Our study underscores the enduring significance of AF/AFL as a prominent public health concern worldwide, marked by profound regional and national variations. Despite the substantial potential for prevention and management of AF/AFL, there is a pressing imperative to adopt more cost-effective strategies and interventions to target modifiable risk factors, particularly in areas where the burden of AF/AFL is high or escalating.

## Graphical Abstract

**Graphical Abstract:**
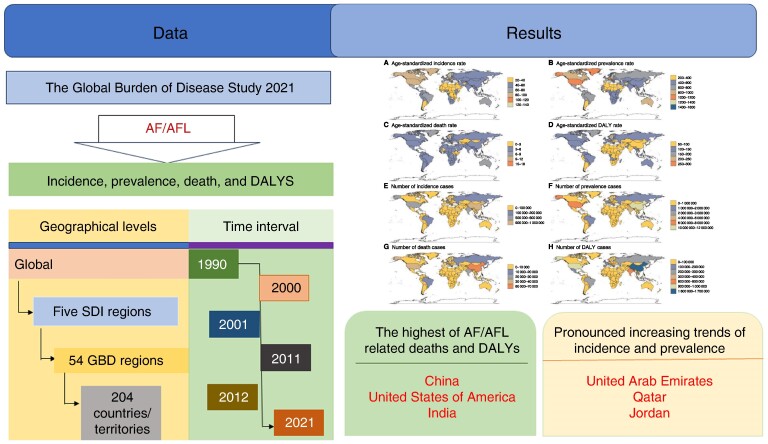

**What’s new?**

- What is already known?
  Previous studies have estimated the risk factors that influence the burden of multiple cardiovascular diseases. Among cardiovascular diseases, atrial fibrillation (AF) and atrial flutter (AFL) are often overlooked by the public because of their relatively low incidence, but they often lead to life-threatening and serious complications that affect public health.
  Among them, some studies related to the Global Burden of Disease (GBD) database on AF/AFL data suggest that the burden of AF/AFL is not limited to developed or less developed countries and a high burden of these diseases was seen in countries with various socio-demographic indices.
- Finally, a comparison of the years of life lost and years lived with disability in AF/AFL by gender leads to the conclusion that more emphasis should be placed on reducing mortality in female AF/AFL patients in the future.
- What does this study add?
  This study mainly focused on the latest data of AF/AFL belong to GBD and analysed multiple indicators from national, regional, and age-standard dimensions to identify potential risk factors including prevalence, deaths, years lived with disability-adjusted life years (DALYs) that influence the development of AF/AFL.
  We have refined the study of AF/AFL to each country in the world, based on the geographical information of the GBD database, and have obtained age-standardized incidence and age-standardized mortality rates for AF/AFL for 204 countries worldwide.
  In addition, we calculated DALYs, which is a more comprehensive indicator to assess disease burden, enabling a better evaluation of disease risk and quality of life of the population. By calculating them, we found that although age-standardized DALYs decreased over the study period, a larger number of DALYs were lost due to AF/AFL over time during the analysed period.
- What do the new findings imply?
  Accurate assessment of the burden of AF/AFL is essential for formulating effective preventative prevention and treatment programmes and optimizing health system resource allocation.
  The number of cases, deaths, and DALYs of AF/AFL showed an overall increasing trend and obvious geographical differences in the past three decades, despite the decreases in age-standardized rates. The burden of cardiomyopathy remains a persistent threat to global public health. These results provide an epidemiological foundation that can guide public health efforts and policymakers.

## Introduction

Atrial fibrillation (AF) and atrial flutter (AFL) are prevalent tachyarrhythmias that can lead to severe complications, including heart failure and stroke, significantly impacting patients’ physical and mental health and quality of life.^1^ The aetiology of AF/AFL is complex, involving risk factors such as hypertension, smoking, alcohol consumption, high-sodium intake, obesity, and socio-demographic variables.^2^ Recent trends indicate a steep rise in AF-related hospitalizations, imposing heavy burdens on patients and the global healthcare system.^3^ Regular assessments of the current AF burden and its risk factors are crucial for developing effective prevention and control strategies.

The global prevalence of AF has doubled from 1990 to 2019, reaching 59.7 million cases in 2019.^4^ This trend is expected to continue. In the USA, AF/AFL affects 3–5 million individuals and is projected to increase to over 8 million by 2050 due to population aging.^5^ In Europe, the prevalence is anticipated to surge from 8.8 million to 18 million by 2060.^6^

Economic globalization has led to disparities in morbidity and mortality among countries. In less developed regions, low cultural and educational levels contribute to a higher disease burden. However, in economically advanced countries with better healthcare resources and education, while AF/AFL incidence is higher, advanced diagnosis and treatment measures improve survival rates.^7^

A previous study evaluated the burden of AF from 1990 to 2019, utilizing data from the Global Burden of Disease (GBD) 2019 study. However, given the advancements in research and the emergence of new data, an updated assessment is paramount. Notably, since the Global Burden of Disease, Injury, and Risk Factor Study 2021 (GBD 2021) update, there has been a lack of a systematic study comprehensively updating the epidemiological trends in AF/AFL. Furthermore, while previous studies have examined the epidemiological aspects of AF in specific time periods within a particular country or region,^8–10^ there is currently no available research that utilizes the recently updated GBD database to analyse the trends of AF/AFL over the span of ∼30 years from 1990 to 2021, as well as the specific epidemiological situation of AF/AFL in 2021. In general, our research is conducted on a broader scale and over a longer duration, using the latest information. Therefore, leveraging the GBD 2021 data, this study aims to conduct a thorough and systematic analysis of the disease burden of AF/AFL. Specifically, it will evaluate the global, regional, and national trends in AF/AFL prevalence, incidence, death, disability-adjusted life years (DALYs), and risk factors. This analysis holds significant value, providing crucial epidemiological information that can inform policy decisions and interventions.

## Methods

### Data sources

The data for this study were sourced from the Global Burden of Diseases, Injuries, and Risk Factors (GBD) 2021 database, a refined version of the GBD 2019 iteration. This comprehensive data set incorporates 100 983 diverse sources to rigorously evaluate the disease burden of 371 diseases and injuries. Among these sources, vital registration systems, cancer registries, and verbal autopsy data play pivotal roles, guaranteeing the utmost accuracy and reliability of the data. Furthermore, to gain deeper insights into the underlying causes and determinants of AF and AFL, GBD 2021 incorporates population-based surveys, epidemiological studies, and published literature. Covering 204 countries across all five socio-demographic index (SDI) regions and 54 GBD regions, GBD 2021 employs a standardized and comparable approach to estimate population size, fertility rates, incidence, prevalence, mortality, and DALYs. Leveraging this robust data set, our study aimed to thoroughly investigate the global burden of AF and AFL. Specifically, we relied on the GBD 2021 database to accurately estimate the global, regional, and national incidence, prevalence, mortality, and DALYs attributable to AF and AFL from 1990 to 2021. The waiver of informed consent was approved by the University of Washington's Institutional Review Board due to the use of de-identified aggregated data.

### Definitions

In the GBD study, we included all forms of AF and AFL, whether paroxysmal, persistent, or permanent/chronic.

The case definition of input data of AF/AFL was offered by GBD 2021 Methods Appendices: (i) irregularly irregular RR intervals (in the absence of complete AV block); (ii) no distinct P waves on the surface ECG; and (iii) an atrial cycle length (when visible) that is typically variable and not exceeding 200 ms. The International Classification of Disease (ICD) codes used for inclusion of hospital and claims are I48I48.9 for ICD-10 and 427.3 for ICD-9 (https://www.healthdata.org/gbd/methods-appendices-2021/atrial-fibrillation-and-flutter).

### Years of life lost and years lived with disability

Years of life lost (YLLs) were calculated by multiplying standard life expectancy at death by the number of deaths. Years lived with disability (YLDs) were estimated as YLLs converted to health status, weighted by disability severity. The sum of YLLs and YLDs comprised DALYs.^11^

### Risk factors

Risk factors for AF/AFL were identified based on causation evidence, exposure data availability, and modification potential.^12,13^ The GBD 2021 identified six risk factors: high body mass index (BMI), elevated systolic blood pressure, smoking, excessive alcohol consumption, high-sodium diet, and lead exposure. Their precise definitions and contribution to AF/AFL-related death are reported in previous studies.^14^

### Age-standardized rates and estimated annual percentage change

In this research, age-standardized rates (ASRs) were employed to quantify the occurrence, prevalence, death, and DALYs associated with AF and AFL. Additionally, we calculated the estimated annual percentage change (EAPC) in prevalence to evaluate the global burden of AF/AFL. Age-standardized rate was determined by adjusting the global age distribution to a standard population,^15^ which is crucial for comparing populations with varying age structures or analysing changes in the age structure of a single group over time. Standardization enables a comparison per 100 000 individuals, offering valuable insights into changes in disease patterns and risk factors within a community. Therefore, ASR serves as a tool for developing more focused and precise approaches for prevention and therapy.

Uncertainty intervals (UIs) were computed based on 1000 draw-level estimates for each parameter, with 95% UI defined as the 25th and 975th values across all draws. Estimated annual percentage change serves as a concise metric for assessing trends in ASR over specific time periods. The regression line follows the natural logarithm of the ratio, *y* = *α* + *βx* + *ɛ*, where *y* = ln(ASR), *x* = calendar year, and *ε* represents the error. Estimated annual percentage change is reported with a 95% confidence interval (CI). An increase in ASR is indicated when EAPC estimates and their lower 95% CI exceed 0, whereas a decrease is suggested when EAPC estimates and their upper 95% CI are less than 0. Furthermore, to investigate the implications of EAPC, we analysed its correlation with ASR 2021 and HDI 2021 nationally.^16^

### Mortality rates

Mortality rates were estimated using population registry data coded with ICD or household surveys. We improved data comparability using statistical methods, including code reclassification, noise reduction, and Bayesian geospatial regression (CODEm),^17^ which leveraged age, space, and time to create smooth trends for 204 countries/territories. Disease incidence and prevalence were derived from diverse population-representative sources, including cohort/registry reports, surveys, registry/cohort microdata, and health system data. Consistent estimates were generated using DisMod-MR and MR-BRT, which were adjusted for methodological and definitional differences.^17^

### Advantage of Global Burden of Disease

The GBD aims to estimate all quantities for each location, even in data scarcity. Best estimates and uncertainties are provided, motivating the search for better global data and statistical methods. This commitment to best estimates distinguishes the GBD from other efforts. Emphasizing comparability across time and geography is crucial for policymakers to understand disease burdens and trends. To ensure comparability, the entire historical time series is recalculated with each GBD release, preventing spurious comparisons.^18^ The principles of data inclusion and use in the GBD database ensure data accuracy.

### Statistical analyses

The estimation of AF/AFL-related disease burden involved several key steps. Firstly, we described the disease burden of AF/AFL globally and in several subtypes, including sexes, age groups, SDI regions, GBD regions, and countries based on the available data. Secondly, we determined the changing trend of AF/AFL by using the linear regression model. To gain further insights into the evolving patterns across diverse GBD regions, we employed a hierarchical cluster analysis based on the EAPC values. Finally, we delved into the risk factors that contribute to the disease burden of AF/AFL.

All statistical analyses were conducted using R software, version 4.3.1, and a two-sided *P*-value of less than 0.05 was deemed statistically significant.

### Ethical approval and consent to participate

The authors are accountable for all aspects of the work in ensuring that questions related to the accuracy or integrity of any part of the work are appropriately investigated and resolved. The study was conducted in accordance with the Declaration of Helsinki (as revised in 2013). All information from the GBD programme is available and free for the public, so the agreement of the medical ethics committee board was not necessary.

## Results

### Global atrial fibrillation/atrial flutter disease burden trends from 1990 to 2021

In 2021, AF/AFL affected 52.55 million individuals worldwide, a 137% increase from 1990. Incidence cases rose 124.0%, yet the age-standardized incidence rate (ASIR) decreased by 7% from 1990 to 2019, with the most substantial decreases in high–middle SDI countries (*Table 1*).

**Table 1:** The incidence cases and age-standardized incidence of AF/AFL in 1990 and 2021, and its temporal trends from 1990 to 2021, by sex, SDI quintile, and GBD region

Characteristics	Number of incidence cases in 1990	ASIR per 100 000 (95% UI)	Number of incidence cases in 2021	ASIR per 100 000 (95% UI)	1990–2021 EAPC (95% CI)
Global	2 006 571 (1 554 971–2 640 379)	52.51 (40.39–69.01)	4 484 926 (3 610 620–5 706 019)	52.12 (41.85–66.23)	−0.07 (−0.1 to 0.04)
Sex					
Female	991 284 (754 261–1 318 501)	47.43 (35.96–62.83)	2 189 116 (1 723 110–2 821 648)	47.26 (37.38–60.87)	−0.14 (−0.19 to 0.09)
Male	1 015 287 (786 876–1 319 475)	58 (44.89–75.8)	2 295 811 (1 853 679–2 899 436)	57.11 (46.19–72.14)	−0.01 (−0.04 to 0.02)
Age					
30–34 years	15 107 (7183–26 341)	3.92 (1.86–6.83)	23 416 (12 042–39 643)	3.87 (1.99–6.56)	0.03 (−0.01 to 0.08)
35–39 years	41 648 (19 800–72 550)	11.82 (5.62–20.6)	65 838 (34 018–111 027)	11.74 (6.07–19.8)	0.03 (−0.05 to 0.11)
40–44 years	65 501 (42 710–93 217)	22.86 (14.91–32.54)	114 394 (78 355–159 571)	22.87 (15.66–31.9)	0.08 (0.01–0.16)
45–49 years	84 207 (45 669–139 230)	36.27 (19.67–59.96)	176 605 (97 586–285 863)	37.3 (20.61–60.37)	0.16 (0.11–0.22)
50–54 years	129 770 (83 189–199 064)	61.05 (39.13–93.65)	280 850 (186 052–418 556)	63.12 (41.82–94.07)	0.18 (0.13–0.24)
55–59 years	176 558 (92 934–301 568)	95.33 (50.18–162.83)	396 964 (222 705–650 354)	100.31 (56.28–164.34)	0.22 (0.18–0.27)
60–64 years	261 806 (174 202–380 009)	163.01 (108.46–236.6)	541 164 (378 939–758 697)	169.09 (118.4–237.06)	0.09 (0.05–0.14)
65–69 years	325 287 (162 174–532 039)	263.16 (131.2–430.42)	716 938 (399 319–1 121 794)	259.91 (144.76–406.68)	−0.07 (−0.1 to 0.04)
70–74 years	307 200 (180 716–452 942)	362.86 (213.46–535.01)	744 978 (484 038–1 048 294)	361.92 (235.15–509.28)	−0.12 (−0.17 to 0.06)
75–79 years	292 707 (150 751–492 956)	475.52 (244.9–800.83)	610 372 (351 355–987 789)	462.81 (266.41–748.98)	−0.22 (−0.29 to 0.14)
80–84 years	192 143 (108 815–307 705)	543.15 (307.6–869.81)	449 218 (280 998–692 292)	512.9 (320.84–790.44)	−0.29 (−0.34 to 0.23)
85–89 years	84 322 (46 129–146 833)	558.01 (305.26–971.69)	239 659 (134 248–412 265)	524.17 (293.62–901.68)	−0.3 (−0.37 to 0.23)
90–94 years	24 343 (11 815–41 169)	568.07 (275.71–960.74)	94 683 (46 423–155 360)	529.27 (259.5–868.45)	−0.3 (−0.37 to 0.22)
95+ years	5971 (2542–11 224)	586.51 (249.66–1102.5)	29 847 (12 724–55 872)	547.62 (233.45–1025.12)	−0.23 (−0.35 to 0.11)
SDI region					
High SDI	722 978 (553 815–943 982)	64.54 (50.18–83.44)	1 334 730 (1 139 377–1 572 638)	65.1 (56.11–76.05)	−0.07 (−0.13 to 0.02)
High–middle SDI	476 340 (365 294–628 545)	48.86 (37.88–63.69)	934 174 (740 417–1 198 977)	47.16 (37.67–60.25)	−0.17 (−0.22 to 0.13)
Middle SDI	454 020 (351 991–596 801)	48.88 (37.12–64.96)	1 330 376 (1 031 639–1 752 211)	51.11 (39.2–67.85)	0.13 (0.11–0.16)
Low–middle SDI	268 077 (206 734–354 728)	49.52 (37.34–65.82)	682 885 (523 053–912 036)	50.99 (38.44–67.86)	0.1 (0.09–0.1)
Low SDI	82 742 (63 353–109 675)	41.74 (31.59–55.83)	198 494 (153 240–261 939)	43.25 (32.7–57.75)	0.12 (0.11–0.14)
GBD region					
Africa	94 649 (72 757–124 599)	37.44 (28.36–50.03)	236 601 (184 192–310 285)	39.67 (30.1–52.99)	0.18 (0.17–0.19)
African region	76 975 (59 174–101 358)	38.39 (29.23–51.28)	190 098 (148 900–248 116)	40.53 (30.93–54.24)	0.17 (0.16–0.18)
America	431 466 (328 004–569 905)	70.35 (54.35–92.79)	1 001 435 (861 625–1 181 844)	73.91 (63.91–87.14)	0.13 (0.09–0.16)
Andean Latin America	10 658 (8333–13 962)	54.4 (41.63–72.43)	33 004 (25 562–43 750)	56.44 (43.34–75.08)	0.2 (0.16–0.24)
Asia	847 177 (658 303–1 110 926)	46.28 (35.31–61.36)	2 316 760 (1 795 505–3 052 653)	46.69 (35.91–61.67)	0.04 (−0.01 to 0.08)
Australasia	17 291 (15 419–19 399)	73 (65.35–81.38)	38 348 (29 260–50 108)	73.34 (57.3–94.35)	0.07 (0.02–0.12)
Advanced Health System	1 022 854 (784 833–1 335 740)	61.89 (48.19–80.1)	1 743 242 (1 467 626–2 087 293)	62.3 (52.86–73.64)	−0.07 (−0.11 to 0.03)
Basic Health System	631 666 (486 720–830 826)	47.18 (35.86–62.71)	1 793 166 (1 393 880–2 337 507)	49.03 (37.82–64.66)	0.1 (0.06–0.15)
Caribbean	15 317 (11 699–20 439)	60.1 (46.18–79.67)	31 921 (24 658–42 125)	59.14 (45.45–78.13)	−0.05 (−0.06 to 0.04)
Central Africa	9540 (7287–12 574)	38.27 (29.03–50.85)	22 890 (17 827–30 032)	38.52 (29.12–51.44)	0 (−0.02 to 0.02)
Central Asia	20 049 (15 184–26 387)	44.05 (33.4–58.03)	36 231 (27 486–47 288)	44.53 (33.54–57.85)	0.02 (0.01–0.03)
Central Europe	76 656 (57 337–101 380)	50.47 (38.22–65.71)	122 626 (94 472–154 039)	55.16 (44.12–68.47)	0.09 (0.03–0.16)
Central Latin America	49 656 (38 646–65 616)	62.71 (48.02–83.5)	154 316 (119 073–205 022)	62.38 (47.92–83.05)	0.01 (0–0.03)
Central Sub-Saharan Africa	7721 (5859–10 151)	40.17 (30.36–53.2)	19 101 (14 928–25 118)	39.56 (29.91–52.82)	−0.07 (−0.08 to 0.06)
Commonwealth High Income	102 045 (80 575–128 314)	66.48 (53.45–82.63)	168 324 (128 748–220 985)	63.45 (49.49–81.27)	−0.21 (−0.3 to 0.12)
Commonwealth Low Income	33 265 (25 661–43 639)	42.03 (31.83–55.46)	95 180 (72 868–125 062)	44.08 (33.33–58.5)	0.16 (0.1–0.21)
Commonwealth Middle Income	269 238 (206 507–357 604)	49.37 (36.94–65.65)	737 282 (558 882–986 926)	51.04 (38.26–67.87)	0.13 (0.11–0.14)
East Asia	321 766 (246 730–424 271)	42.98 (32.71–56.93)	953 898 (737 314–1 249 815)	45.11 (35.12–59.61)	0.15 (0.06–0.23)
East Asia and Pacific—WB	556 485 (431 578–728 024)	45.56 (34.86–60.23)	1 502 124 (1 166 947–1 960 812)	45.88 (35.78–60.38)	0.02 (−0.06 to 0.11)
Eastern Africa	23 006 (17 775–30 254)	36.85 (28.1–49.14)	59 825 (46 912–77 352)	40.03 (30.52–53.6)	0.25 (0.22–0.29)
Eastern Europe	130 200 (98 553–170 809)	46.52 (35.77–60.37)	176 794 (133 418–231 758)	50.47 (38.83–65.64)	0.3 (0.24–0.37)
Eastern Mediterranean Region	64 362 (48 685–85 764)	40.64 (30.01–54.79)	168 555 (129 707–220 844)	41.84 (31.02–56.11)	0.09 (0.09–0.1)
Eastern Sub-Saharan Africa	24 732 (19 155–32 329)	36.74 (28.1–49.06)	62 823 (49 401–81 796)	39.66 (30.44–52.91)	0.23 (0.2–0.27)
Europe	628 460 (482 090–820 171)	59.88 (46.4–77.32)	921 025 (743 812–1 134 137)	59.26 (48.17–72.45)	−0.15 (−0.19 to 0.12)
Europe and Central Asia—WB	640 726 (491 835–836 075)	59.44 (46.01–76.78)	944 647 (762 072–1 162 893)	58.71 (47.61–71.92)	−0.16 (−0.19 to 0.12)
European Region	645 411 (495 378–842 226)	59.47 (46.04–76.83)	957 812 (773 899–1 178 186)	58.93 (47.79–72.11)	−0.14 (−0.18 to 0.11)
High-income Asia Pacific	86 518 (66 803–113 257)	43.22 (33.54–56.82)	152 471 (116 375–199 323)	36.27 (29.16–46.41)	−0.57 (−0.81 to 0.33)
High-income North America	278 728 (205 978–367 024)	77.6 (59.7–101.77)	586 320 (534 188–649 281)	88.24 (80.92–97.25)	0.41 (0.36–0.46)
Latin America and Caribbean—WB	155 084 (120 428–204 193)	60.29 (46.41–80.24)	419 759 (326 058–552 907)	59.78 (46.2–79.1)	−0.09 (−0.12 to 0.05)
Limited Health System	330 401 (253 868–438 192)	47.81 (35.93–63.52)	902 933 (688 786–1 206 282)	49.54 (37.21–65.91)	0.13 (0.12–0.14)
Middle East and North Africa—WB	38 963 (29 402–51 606)	36.34 (26.69–48.88)	122 751 (95 491–159 162)	39.26 (29.24–51.99)	0.26 (0.23–0.29)
Minimal Health System	19 236 (14 736–25 370)	36.17 (27.55–48.33)	41 319 (32 318–53 954)	36.82 (28.01–49.14)	0.05 (0.04–0.06)
North Africa and Middle East	49 894 (38 440–65 909)	33.93 (25.04–45.3)	145 164 (114 585–186 086)	35.04 (26.72–45.54)	0.03 (0.01–0.05)
North America	278 730 (205 977–367 029)	77.6 (59.7–101.76)	586 333 (534 192–649 308)	88.23 (80.92–97.24)	0.41 (0.36–0.46)
Northern Africa	18 164 (13 699–24 225)	33.99 (24.85–45.85)	50 230 (37 855–66 426)	36.18 (26.69–48.45)	0.18 (0.18–0.19)
Oceania	1295 (1006–1696)	51.68 (39.37–68.62)	3484 (2741–4545)	52.33 (39.94–69.52)	0.04 (0.02–0.06)
Region of the Americas	431 466 (328 004–569 905)	70.35 (54.35–92.79)	1 001 435 (861 625–1 181 844)	73.91 (63.91–87.14)	0.13 (0.09–0.16)
South-East Asia Region	318 971 (245 989–420 440)	52.28 (39.47–69.41)	900 234 (685 905–1 204 221)	53.02 (40.03–70.51)	0.06 (0.05–0.06)
South Asia	249 150 (190 686–331 288)	50.08 (37.35–66.5)	698 920(527 866–934 818)	51.01 (38.09–67.89)	0.08 (0.07–0.08)
South Asia—WB	256 475 (196 479–341 026)	49.93 (37.25–66.32)	716 509 (541 397–958 398)	50.98 (38.09–67.83)	0.08 (0.07–0.09)
Southeast Asia	135 279 (105 015–176 227)	58.77 (44.77–78.09)	369 518 (285 921–483 602)	59.66 (45.6–79.28)	0.07 (0.06–0.08)
Southern Africa	17 300 (13 410–22 718)	43.7 (33.2–58.43)	38 853 (30 201–50 898)	44.59 (33.95–59.42)	0.04 (0.03–0.06)
Southern Latin America	19 573 (14 786–26 223)	42.83 (32.53–57.25)	28 852 (23 283–35 802)	33 (26.86–40.72)	−0.93 (−1.14 to 0.73)
Southern Sub-Saharan Africa	11 862 (9126–15 604)	47.32 (35.92–63.2)	25 556 (19 614–33 834)	47.6 (36.27–63.27)	0 (−0.01 to 0.01)
Sub-Saharan Africa—WB	76 773 (59 193–100 905)	38.39 (29.24–51.28)	186 914 (146 216–243 323)	40.71 (31.04–54.52)	0.19 (0.18–0.2)
Tropical Latin America	60 437 (46 588–78 717)	68.71 (52.98–90.29)	172 830 (133 612–224 369)	67.55 (52.09–88.35)	−0.24 (−0.29 to 0.19)
Western Africa	26 639 (20 444–35 254)	36.71 (27.89–49.3)	64 803 (50 701–84 637)	40.03 (30.53–53.49)	0.32 (0.3–0.34)
Western Europe	410 476 (310 302–539 191)	69.97 (54.22–90.22)	600 735 (491 661–740 671)	68.19 (56.6–82.27)	−0.25 (−0.31 to 0.18)
Western Pacific Region	456 416 (352 936–596 877)	43.4 (33.28–57.43)	1 235 061 (959 063–1 617 546)	43.94 (34.32–57.83)	0.05 (−0.06–0.15)
Western Sub-Saharan Africa	29 311 (22 484–38 784)	36.54 (27.78–49.07)	72 014 (56 373–94 024)	39.82 (30.36–53.28)	0.31 (0.29–0.34)
World Bank High Income	877 638 (670 891–1 149 645)	65.65 (50.94–84.68)	1 539 259 (1 305 981–1 830 970)	65.75 (56.34–77.11)	−0.1 (−0.15 to 0.05)
World Bank Low Income	45 614 (35 271–60 031)	37.75 (28.79–50.26)	106 133 (83 284–138 198)	39.07 (29.73–52.07)	0.11 (0.1–0.12)
World Bank Lower Middle Income	467 409 (360 579–618 460)	48.61 (36.88–64.58)	1 229 044 (942 518–1 636 405)	50.66 (38.34–67.51)	0.14 (0.13–0.16)
World Bank Upper Middle Income	613 489 (475 778–802 697)	45.57 (35–59.98)	1 606 205 (1 249 213–2 086 053)	47.81 (37.21–62.46)	0.12 (0.07–0.16)

ASIR, age-standardized incidence rate; CI, confidence interval; EAPC, estimated annual percentage change; UI, uncertainty interval.

Atrial fibrillation/AFL-related deaths totalled 0.34 million in 2021, a 196.0% increase since 1990, with the greatest increases in low–middle SDI countries. Atrial fibrillation/AFL contributed to 8.36 million DALYs in 2021, a 149.0% increase from 1990. Despite this, the age-standardized DALYs rate remained stable, with the most significant decreases in high–middle SDI countries (*Table 4*).

**Figure 1:**
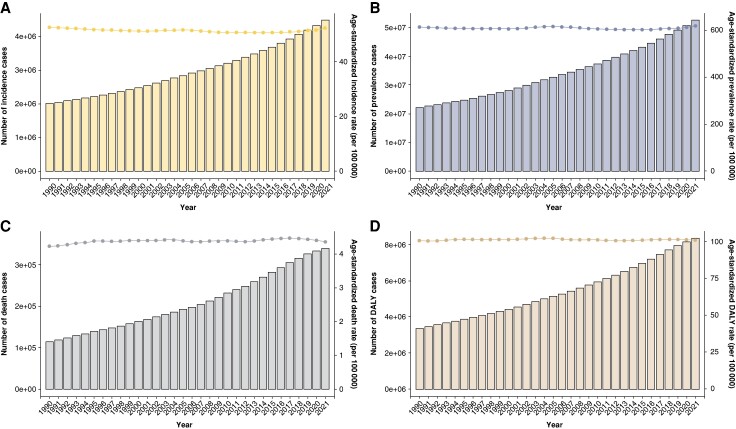
Age-standardized rate of incidence, prevalence, deaths, and DALYs change curves for AF/AFL patients from 1990 to 2021 (*A*: incidence; *B*: prevalence; *C*: deaths; *D*: DALYs), AF, atrial fibrillation; AFL, atrial flutter; DAYLs, disability-adjusted life years.

Furthermore, our study revealed a consistent trend from 1990 to 2021: the ASR of incidence, prevalence, deaths, and DALYs associated with AF/AFL all displayed a gradual upward trajectory (*Figure 1*).

Age groups 70–79 exhibited the highest AF/AFL prevalence, while 65–74 showed higher incidence rates (see Supplementary material online, *Figure S1*; *Tables 1* and *2*). Unexpectedly, these groups had lower death rates than those over 80. The 80–89 age groups had higher death numbers (see Supplementary material online, *Figure S1*; *Table 3*), while the 80–84 age group exhibited the highest DALYs (see Supplementary material online, *Figure S1*; *Table 4*). The ASRs for AF/AFL prevalence, incidence, death, and DALYs were generally consistent, except for the >95 age group, which had the highest ASRs for death and DALYs, as well as higher ASRs for prevalence and incidence (see Supplementary material online, *Figure S1*; *Tables 1–4*).

**Table 2:** The prevalence cases and age-standardized prevalence of AF/AFL in 1990 and 2021, and its temporal trends from 1990 to 2021, by sex, SDI quintile, and GBD region

Characteristics	Number of prevalence cases in 1990	ASPR per 100 000 (95% UI)	Number of prevalence cases in 2021	ASPR per 100 000 (95% UI)	1990–2021 EAPC (95% CI)
Global	22 214 495 (17 526 215–28 522 555)	616.58 (485.22–795.26)	52 552 045 (43 137 876–64 963 854)	620.51 (511.36–768.88)	−0.01 (−0.04 to 0.01)
Sex					
Female	10 722 025 (8 387 109–13 889 787)	529.53 (413.11–690.99)	24 652 999 (20 031 485–30 931 400)	529.12 (430.79–663.14)	−0.11 (−0.14 to 0.07)
Male	11 492 470 (9 169 280–14 778 457)	727.79 (578.43–929.67)	27 899 046 (23 026 328–34 646 024)	728.88 (601.91–895.81)	0.05 (0.01–0.08)
Age					
30–34 years	25 623 (12 183–44 678)	6.65 (3.16–11.59)	39 719 (20 427–67 237)	6.57 (3.38–11.12)	0.03 (−0.01 to 0.08)
35–39 years	162 149 (77 064–282 497)	46.03 (21.88–80.2)	256 344 (132 418–432 320)	45.71 (23.61–77.08)	0.03 (−0.05 to 0.11)
40–44 years	377 636 (215 997–616 168)	131.82 (75.4–215.08)	656 235 (399 610–1 037 908)	131.18 (79.88–207.48)	0.08 (−0.01 to 0.18)
45–49 years	646 620 (423 376–934 929)	278.48 (182.34–402.65)	1 353 910 (928 807–1 903 644)	285.93 (196.16–402.03)	0.18 (0.11–0.25)
50–54 years	1 095 609 (742 124–1 567 304)	515.41 (349.12–737.31)	2 389 266 (1 675 385–3 345 918)	537.01 (376.56–752.02)	0.22 (0.17–0.28)
55–59 years	1 665 495 (1 148 311–2 351 318)	899.3 (620.04–1269.61)	3 768 098 (2 703 971–5 140 067)	952.19 (683.29–1298.89)	0.24 (0.2–0.29)
60–64 years	2 443 603 (1 710 856–3 502 831)	1521.46 (1065.23–2180.97)	5 129 048 (3 785 334–7 130 208)	1602.59 (1182.74–2227.86)	0.21 (0.16–0.25)
65–69 years	3 171 531 (2 332 473–4 302 603)	2565.77 (1886.97–3480.8)	7 295 372 (5 581 877–9 525 430)	2644.76 (2023.58–3453.22)	0.1 (0.07–0.14)
70–74 years	3 430 276 (2 436 200–4 711 283)	4051.77 (2877.59–5564.87)	8 620 198 (6 527 851–11 285 872)	4187.83 (3171.33–5482.86)	0.03 (0.01–0.05)
75–79 years	3 824 489 (2 716 413–5 185 707)	6213.07 (4412.95–8424.44)	8 126 007 (6 203 968–10 550 937)	6161.46 (4704.1–8000.14)	−0.07 (−0.12 to 0.03)
80–84 years	3 065 788 (2 137 338–4 230 387)	8666.31 (6041.79–11 958.38)	7 390 279 (5 575 337–9 773 322)	8438.02 (6365.77–11 158.92)	−0.18 (−0.23 to 0.14)
85–89 years	1 644 788 (1 160 635–2 253 811)	10 884.66 (7680.69–14 914.96)	4 783 628 (3 657 230–6 243 735)	10 462.47 (7998.88–13 655.93)	−0.22 (−0.25 to 0.19)
90–94 years	533 159 (379 666–727 836)	12 441.9 (8859.97–16 984.93)	2 116 059 (1 620 933–2 759 252)	11 828.58 (9060.87–15 423.97)	−0.25 (−0.29 to 0.22)
95+ years	127 728 (92 690–172 440)	12 545.93 (9104.3–16 937.64)	627 882 (481 052–811 292)	11 520.13 (8826.14–14 885.24)	−0.4 (−0.48 to 0.32)
SDI region					
High SDI	8 631 675 (6 800 989–11 018 130)	766.6 (608.09–974.16)	17 401 068 (15 107 026–20 202 156)	788.35 (690.97–910.9)	0.01 (−0.04 to 0.06)
High-middle SDI	5 443 470 (4 254 251–7 001 406)	587.77 (464.15–754.8)	11 539 353 (9 330 859–14 532 463)	581.39 (473.47–731.22)	−0.07 (−0.11 to 0.03)
Middle SDI	4 673 673 (3 692 430–6 104 440)	537.07 (422.34–692.7)	14 670 503 (11 595 532–19 210 983)	579.06 (457.58–748.76)	0.27 (0.23–0.3)
Low-middle SDI	2 632 144 (2 062 576–3 472 861)	524.91 (409.36–683.63)	6 929 826 (5 413 491–9 033 562)	546.49 (425.68–711.16)	0.14 (0.13–0.14)
Low SDI	806 009 (629 010–1 060 706)	442.34 (345.89–576.61)	1 959 344 (1 535 182–2 587 655)	463.23 (362.02–602.71)	0.16 (0.14–0.17)
GBD region					
Africa	941 460 (736 073–1 235 896)	400.94 (313.8–522.72)	2 360 173 (1 857 776–3 087 042)	432.2 (338.56–561.67)	0.24 (0.23–0.25)
African Region	775 290 (607 120–1 018 272)	416.1 (326.66–543.17)	1 910 957 (1 503 222–2 506 203)	445.11 (349.88–580.51)	0.21 (0.2–0.22)
America	4 940 658 (3 856 859–6 355 883)	818.85 (638.14–1051.33)	11 838 087 (10 333 854–13 745 300)	865.5 (757.2–1002.86)	0.18 (0.15–0.21)
Andean Latin America	112 466 (88 857–146 494)	599.92 (471.7–773.74)	371 258 (290 314–478 820)	647.36 (505.19–835.55)	0.33 (0.29–0.37)
Asia	8 735 972 (6 929 130–11 403 331)	507.59 (401.04–652.62)	25 739 092 (20 431 798–33 586 165)	529.04 (420.26–680.91)	0.17 (0.11–0.22)
Australasia	208 038 (190 582–225 387)	885.17 (810.92–960.4)	508 534 (400 838–650 591)	913.63 (725.73–1163.47)	0.2 (0.14–0.25)
Advanced Health System	12 222 732 (9 640 972–15 612 567)	741.05 (586.99–942.34)	22 809 374 (19 494 004–26 913 584)	758.79 (653.83–888.91)	0 (−0.04 to 0.03)
Basic Health System	6 595 731 (5 212 030–8 602 425)	521.5 (409.74–673.94)	20 312 357 (16 149 649–26 488 232)	565.23 (448.8–734.45)	0.28 (0.23–0.33)
Caribbean	168 732 (130 817–218 385)	677.46 (527.46–877.57)	367 640 (289 060–476 682)	678.59 (533.41–881.12)	0 (−0.01 to 0.01)
Central Africa	93 396 (72 260–121 487)	409.11 (318.66–536.18)	222 244 (172 762–293 087)	417.19 (325.98–548.17)	0.03 (0.02–0.05)
Central Asia	222 460 (171 711–290 357)	513.48 (395.59–666.95)	392 829 (305 783–509 896)	536.72 (410.76–698.74)	0.14 (0.13–0.15)
Central Europe	901 372 (699 705–1 154 305)	616.69 (478.07–790.3)	1 585 129 (1 281 858–1 949 452)	679 (554.31–827.23)	0.07 (−0.01 to 0.15)
Central Latin America	524 834 (413 226–681 703)	702.85 (548.24–910.96)	1 711 299 (1 340 757–2 226 156)	707.02 (552.56–921.13)	0.05 (0.04–0.07)
Central Sub-Saharan Africa	73 688 (57 051–95 882)	427.06 (330.92–558.11)	182 594 (142 529–241 993)	424.57 (331.96–559.66)	−0.05 (−0.06 to 0.04)
Commonwealth High Income	1 210 095 (991 971–1 481 117)	775.44 (641.5–944.83)	2 206 051 (1 744 928–2 807 667)	776.81 (623.32–980.84)	−0.04 (−0.12 to 0.05)
Commonwealth Low Income	334 217 (260 342–437 906)	451.09 (351.94–588.94)	974 439 (758 654–1 285 016)	481.26 (375.71–622.66)	0.2 (0.13–0.27)
Commonwealth Middle Income	2 575 238 (2 004 475–3 401 695)	515.89 (400.14–677.12)	7 289 406 (5 639 477–9 559 055)	534.76 (414.74–699.12)	0.14 (0.12–0.15)
East Asia	3 359 584 (2 649 140–4 376 801)	462.71 (363.3–601.04)	11 215 165 (8 885 909–14 572 495)	526.44 (420.6–683.56)	0.46 (0.36–0.55)
East Asia and Pacific—WB	5 951 516 (4 725 492–7 716 784)	510.85 (405.09–659.19)	17 648 921 (14 078 772–22 918 938)	538.33 (430.97–698.4)	0.21 (0.12–0.29)
Eastern Africa	233 016 (182 584–305 069)	403.02 (317.12–525.1)	613 141 (484 225–794 335)	449.74 (354.47–589.63)	0.36 (0.32–0.4)
Eastern Europe	1 601 348 (1 245 865–2 080 668)	584.41 (454.97–753.25)	2 327 313 (1 805 306–3 029 889)	648.92 (507–836.25)	0.39 (0.32–0.47)
Eastern Mediterranean Region	617 722 (482 076–807 811)	419.72 (324.21–551.04)	1 620 763 (1 270 894–2 108 348)	439.05 (338.82–577.02)	0.14 (0.13–0.15)
Eastern Sub-Saharan Africa	253 297 (198 232–333 959)	407.55 (320.71–534.37)	645 690 (507 531–839 253)	448.76 (354.06–589.55)	0.31 (0.27–0.35)
Europe	7 541 663 (5 933 601–9 618 858)	718.21 (567.84–913.28)	12 501 839 (10 430 278–15 157 563)	745.07 (623.4–894.31)	0 (−0.06 to 0.06)
Europe and Central Asia—WB	7 677 156 (6 038 274–9 794 201)	712.64 (563.2–906.75)	12 750 321 (10 623 543–15 489 013)	738.74 (617.05–888.34)	0 (−0.06 to 0.06)
European Region	7 731 573 (6 080 359–9 864 457)	713.03 (563.47–907.35)	12 922 170 (10 770 948–15 682 290)	741.43 (619.28–890.72)	0.01 (−0.04 to 0.07)
High-income Asia Pacific	1 052 102 (825 876–1 364 552)	533.61 (423.32–688.79)	2 165 450 (1 737 905–2 727 227)	465.22 (383.84–577.04)	−0.35 (−0.57 to 0.12)
High-income North America	3 293 625 (2 543 345–4 240 128)	900.45 (701.18–1153.05)	7 106 415 (6 544 296–7 755 026)	1031.17 (952.3–1117.88)	0.45 (0.4–0.51)
Latin America and Caribbean—WB	1 673 632 (1 320 723–2 176 946)	683.84 (539.07–880.28)	4 790 261 (3 804 195–6 151 981)	690.58 (546.33–885.8)	0.03 (0.01–0.06)
Limited Health System	3 178 402 (2 478 060–4 199 624)	501.04 (389.98–655.87)	8 972 444 (6 971 956–11 767 643)	522.78 (406.55–682.52)	0.16 (0.15–0.17)
Middle East and North Africa—WB	375 472 (290 501–488 856)	380.85 (290.8–498.53)	1 234 540 (982 525–1 572 313)	430.42 (336.65–554.31)	0.42 (0.37–0.46)
Minimal Health System	190 105 (148 322–248 601)	385.58 (301.59–504.55)	405 919 (318 040–533 566)	400.11 (313.12–525.02)	0.1 (0.09–0.11)
North Africa and Middle East	474 266 (369 341–613 686)	345.93 (267.05–451.62)	1 417 367 (1 140 557–1 788 911)	366.92 (291.62–468.4)	0.1 (0.07–0.14)
North America	3 293 680 (2 543 371–4 240 208)	900.4 (701.15–1153)	7 106 694 (6 544 488–7 755 397)	1031.09 (952.22–1117.81)	0.45 (0.4–0.51)
Northern Africa	170 923 (131 521–222 736)	345.44 (263.42–451.45)	488 952 (377 680–633 795)	382.73 (291.74–503.08)	0.31 (0.3–0.32)
Oceania	12 769 (10 040–16 711)	558.23 (439.17–726.94)	34 994 (27 519–45 179)	578.44 (455.22–750.19)	0.11 (0.09–0.12)
Region of the Americas	4 940 658 (3 856 859–6 355 883)	818.85 (638.14–1051.33)	11 838 087 (10 333 854–13 745 300)	865.5 (757.2–1002.86)	0.18 (0.15–0.21)
South-East Asia Region	3 072 847 (2 405 905–4 056 426)	549.41 (428.11–715.54)	9 073 737 (7 060 465–11 839 908)	562.44 (439.23–732.19)	0.08 (0.07–0.09)
South Asia	2 357 498 (1 827 475–3 099 820)	519.49 (401.88–683.87)	6 882 583 (5 295 961–9 026 139)	530.9 (410.19–696.69)	0.09 (0.08–0.1)
South Asia—WB	2 431 655 (1 885 931–3 198 053)	518.43 (401.36–682.64)	7 073 322 (5 450 120–9 275 204)	531.76 (411.28–697.32)	0.1 (0.09–0.11)
Southeast Asia	1 377 746 (1 089 207–1 799 890)	637.21 (502.03–823.86)	3 898 608 (3 091 004–5 118 881)	662.33 (519.61–854.36)	0.15 (0.13–0.16)
Southern Africa	175 743 (138 141–232 322)	475.82 (372.82–617.82)	390 977 (307 359–516 695)	486.35 (380.84–635.4)	0.04 (0.03–0.05)
Southern Latin America	212 016 (165 906–278 675)	475.26 (372.44–622.31)	348 813 (291 741–425 439)	391.08 (328.22–476.31)	−0.7 (−0.89 to 0.52)
Southern Sub-Saharan Africa	121 142 (94 906–158 838)	510.12 (398.24–662.2)	258 590 (201 941–342 437)	512.78 (398.19–671.03)	−0.01 (−0.02 to 0)
Sub-Saharan Africa—WB	773 516 (606 447–1 014 049)	416.4 (327.23–542.96)	1 877 042 (1 476 213–2 455 889)	448.06 (352.13–583.98)	0.24 (0.23–0.25)
Tropical Latin America	661 730 (521 046–861 787)	802.54 (631.12–1040.53)	2 004 719 (1 580 577–2 599 905)	794.78 (625.65–1025.18)	−0.07 (−0.09 to 0.06)
Western Africa	268 383 (209 722–355 329)	395.12 (307.42–517.94)	644 858 (505 872–851 041)	436.78 (340.72–569.7)	0.37 (0.34–0.39)
Western Europe	4 930 276 (3 885 223–6 301 409)	817.69 (649.57–1042.91)	8 410 140 (7 063 579–10 013 004)	844.93 (717.15–992.46)	−0.03 (−0.11 to 0.05)
Western Pacific Region	4 930 371 (3 920 786–6 383 438)	489.77 (388.41–631.09)	14 799 624 (11 807 166–19 225 175)	521.93 (418.59–676.77)	0.26 (0.16–0.36)
Western Sub-Saharan Africa	295 505 (230 745–390 715)	393.31 (305.99–515.67)	716 915 (561 805–945 355)	434.84 (339.54–567.05)	0.36 (0.34–0.39)
World Bank High Income	10 476 033 (8 244 581–13 417 137)	778.69 (616.76–991.8)	20 210 628 (17 363 354–23 544 726)	793.33 (690.8–919.75)	−0.03 (−0.07 to 0.02)
World Bank Low Income	455 301 (355 441–592 162)	404.43 (317.96–525.41)	1 074 925 (847 544–1 400 599)	429.65 (338.59–558.51)	0.2 (0.19–0.22)
World Bank Lower Middle Income	4 632 759 (3 622 773–6 109 074)	521.31 (405.61–679.33)	12 409 919 (9 701 543–16 240 678)	543.37 (424.19–708.56)	0.14 (0.13–0.16)
World Bank Upper Middle Income	6 622 791 (5 227 787–8 616 813)	520.99 (411.68–671.72)	18 804 420 (14 950 846–24 416 302)	563.57 (450.26–730.36)	0.27 (0.22–0.32)

ASPR, age-standardized prevalence rate; CI, confidence interval; EAPC, estimated annual percentage change; SDI, socio-demographic index; UI, uncertainty interval.

**Table 3:** The deaths cases and ASDR of AF/AFL in 1990 and 2021, and its temporal trends from 1990 to 2021, by sex, SDI quintile, and GBD region

Characteristics	Number of deaths cases in 1990	ASDR per 100 000 (95% UI)	Number of deaths cases in 2021	ASDR per 100 000 (95% UI)	1990–2021 EAPC (95% CI)
Global	114 540 (101 326–127 155)	4.24 (3.69–4.71)	338 947 (288 954–368 613)	4.36 (3.69–4.75)	0.1 (0.06–0.13)
Sex					
Female	71 862 (63 274–81 196)	4.25 (3.66–4.79)	204 247 (167 703–228 405)	4.29 (3.53–4.8)	0.02 (−0.02 to 0.06)
Male	42 677 (37 233–46 516)	4.2 (3.65–4.59)	134 700 (120 296–145 862)	4.44 (3.94–4.81)	0.21 (0.18–0.24)
Age					
30–34 years	69 (57–82)	0.02 (0.01–0.02)	115 (99–131)	0.02 (0.02–0.02)	0.19 (0.1–0.28)
35–39 years	100 (86–119)	0.03 (0.02–0.03)	175 (155–194)	0.03 (0.03–0.03)	0.26 (0.19–0.34)
40–44 years	249 (215–293)	0.09 (0.07–0.1)	458 (408–506)	0.09 (0.08–0.1)	−0.01 (−0.09 to 0.08)
45–49 years	555 (476–653)	0.24 (0.2–0.28)	1089 (965–1202)	0.23 (0.2–0.25)	−0.17 (−0.22 to 0.13)
50–54 years	1229 (1059–1438)	0.58 (0.5–0.68)	2396 (2128–2605)	0.54 (0.48–0.59)	−0.27 (−0.32 to 0.23)
55–59 years	2322 (2012–2716)	1.25 (1.09–1.47)	4777 (4243–5259)	1.21 (1.07–1.33)	−0.15 (−0.2 to 0.11)
60–64 years	3981 (3510–4608)	2.48 (2.19–2.87)	7691 (6941–8219)	2.4 (2.17–2.57)	−0.15 (−0.19 to 0.12)
65–69 years	5853 (5239–6650)	4.73 (4.24–5.38)	12 516 (11 315–13 448)	4.54 (4.1–4.88)	−0.17 (−0.21 to 0.12)
70–74 years	9667 (8623–10 986)	11.42 (10.19–12.98)	23 527 (21 468–25 286)	11.43 (10.43–12.28)	−0.1 (−0.16 to 0.04)
75–79 years	16 700 (15 216–18 461)	27.13 (24.72–29.99)	34 926 (30 951–37 685)	26.48 (23.47–28.57)	−0.06 (−0.1 to 0.02)
80–84 years	26 568 (23 684–29 178)	75.1 (66.95–82.48)	66 389 (57 921–71 821)	75.8 (66.13–82)	0.03 (−0.01 to 0.08)
85–89 years	25 172 (21 746–27 799)	166.58 (143.9–183.96)	78 629 (65 155–86 580)	171.97 (142.5–189.36)	0.16 (0.11–0.22)
90–94 years	16 532 (13 631–18 384)	385.8 (318.11–429)	73 759 (57 832–82 367)	412.31 (323.28–460.42)	0.23 (0.18–0.28)
95+ years	5543 (4299–6275)	544.41 (422.26–616.37)	32 499 (24 353–37 046)	596.28 (446.81–679.71)	0.22 (0.11–0.33)
SDI region					
High SDI	50 188 (44 165–53 072)	4.74 (4.14–5.03)	125 622 (103 230–137 669)	4.66 (3.88–5.08)	−0.01 (−0.06 to 0.04)
High-middle SDI	28 089 (25 067–30 559)	4.23 (3.69–4.63)	79 214 (67 067–88 012)	4.29 (3.62–4.77)	0.08 (0.03–0.13)
Middle SDI	22 063 (19 086–25 973)	4.18 (3.6–4.92)	83 853 (70 992–94 818)	4.26 (3.57–4.83)	−0.06 (−0.13 to 0.02)
Low-middle SDI	10 458 (7579–14 095)	3.07 (2.26–4.06)	39 263 (32 069–46 278)	4.08 (3.34–4.81)	1 (0.91–1.08)
Low SDI	3568 (2077–5030)	3.05 (1.79–4.32)	10 623 (7284–13 876)	3.74 (2.57–4.9)	0.87 (0.63–1.12)
GBD region					
Africa	5652 (4045–7088)	3.8 (2.75–4.78)	14 580 (11 839–17 231)	4.12 (3.32–4.8)	0.21 (0.16–0.27)
African Region	4569 (3226–5801)	3.92 (2.81–4.97)	11 578 (9298–13 913)	4.19 (3.38–4.96)	0.13 (0.07–0.18)
America	23 026 (20 355–24 387)	4.18 (3.66–4.44)	68 568 (57 542–74 531)	4.84 (4.08–5.25)	0.47 (0.41–0.53)
Andean Latin America	740 (634–855)	4.61 (3.97–5.3)	2079 (1699–2494)	3.81 (3.11–4.57)	−0.78 (−0.94 to 0.61)
Asia	39 389 (32 588–48 147)	3.64 (3.02–4.47)	153 470 (127 841–172 464)	3.84 (3.16–4.33)	0.03 (−0.03 to 0.1)
Australasia	1436 (1281–1525)	6.99 (6.16–7.45)	4337 (3529–4789)	6.58 (5.39–7.25)	0 (−0.14 to 0.14)
Advanced Health System	67 929 (60 972–71 579)	4.57 (4.06–4.84)	165 406 (137 851–180 403)	4.6 (3.88–5)	0.07 (0.03–0.12)
Basic Health System	33 124 (28 509–38 269)	4.33 (3.68–5.03)	122 234 (102 435–139 362)	4.35 (3.61–4.98)	−0.08 (−0.19 to 0.03)
Caribbean	1098 (985–1194)	5.59 (4.99–6.07)	2731 (2349–3048)	4.86 (4.2–5.42)	−0.42 (−0.51 to 0.33)
Central Africa	575 (375–790)	4.09 (2.67–5.61)	1542 (1090–2153)	4.55 (3.32–6.28)	0.24 (0.11–0.37)
Central Asia	684 (596–799)	1.87 (1.61–2.2)	1386 (1241–1519)	2.37 (2.09–2.6)	0.69 (0.43–0.95)
Central Europe	5931 (5508–6253)	5.1 (4.66–5.4)	11 104 (9859–11 914)	4.55 (4.03–4.88)	−0.21 (−0.41 to 0.02)
Central Latin America	2816 (2613–2927)	4.98 (4.56–5.21)	10 354 (8980–11 386)	4.55 (3.95–5.01)	−0.32 (−0.38 to 0.26)
Central Sub-Saharan Africa	439 (278–633)	4.11 (2.55–5.93)	1311 (886–1982)	4.67 (3.16–7.09)	0.36 (0.19–0.54)
Commonwealth High Income	8202 (7359–8624)	5.56 (4.94–5.86)	17 978 (15 000–19 477)	5.4 (4.53–5.84)	0 (−0.06 to 0.07)
Commonwealth Low Income	1720 (965–2432)	3.21 (1.8–4.54)	6349 (4515–8689)	4.49 (3.23–6.08)	0.89 (0.7–1.09)
Commonwealth Middle Income	9113 (6198–13 056)	2.69 (1.9–3.82)	37 601 (29 266–45 758)	3.68 (2.88–4.47)	1.17 (0.98–1.36)
East Asia	17 272(14 013–21 292)	4.94 (3.92–6.07)	67 666 (54 232–80 814)	4.3 (3.42–5.18)	−0.63 (−0.8 to 0.45)
East Asia and Pacific—WB	29 677 (25 385–34 949)	4.24 (3.54–4.98)	110 820 (90 873–127 792)	3.87 (3.16–4.48)	−0.51 (−0.6 to 0.41)
Eastern Africa	1188 (679–1622)	3.18 (1.86–4.4)	2975 (1963–4164)	3.21 (2.11–4.47)	−0.05 (−0.18 to 0.07)
Eastern Europe	8133 (7308–8953)	3.87 (3.44–4.3)	15 697 (14 042–17 056)	4.33 (3.87–4.71)	0.26 (0.15–0.37)
Eastern Mediterranean Region	3361 (2447–4411)	3.19 (2.3–4.19)	10 321 (8504–11 781)	3.94 (3.21–4.51)	0.63 (0.58–0.69)
Eastern Sub-Saharan Africa	1263 (687–1765)	3.17 (1.73–4.55)	3166 (1927–4731)	3.28 (1.97–4.89)	0.06 (−0.08 to 0.2)
Europe	46 117 (41 923–48 556)	5 (4.48–5.29)	101 544 (85 514–110 332)	5.15 (4.37–5.58)	0.27 (0.21–0.33)
Europe and Central Asia—WB	46 446 (42 163–48 945)	4.91 (4.4–5.21)	102 125 (86 140–110 922)	5.1 (4.33–5.52)	0.29 (0.23–0.35)
European Region	46 783 (42 474–49 297)	4.92 (4.4–5.22)	103 043 (86 887–111 924)	5.1 (4.33–5.52)	0.29 (0.23–0.35)
High-income Asia Pacific	5018 (4465–5373)	3.06 (2.68–3.3)	17 112 (13 415–19 331)	2.46 (2.01–2.74)	−1.27 (−1.61 to 0.93)
High-income North America	14 544 (12 556–15 549)	3.96 (3.41–4.24)	39 066 (32 116–42 759)	5.15 (4.27–5.61)	0.79 (0.72–0.87)
Latin America and Caribbean—WB	8658 (7910–9088)	4.72 (4.25–4.98)	29 903 (25 705–32 529)	4.48 (3.85–4.87)	−0.09 (−0.13 to 0.04)
Limited Health System	12 263 (8105–17 445)	2.88 (1.93–4.04)	48 384 (37 312–59 001)	3.82 (2.96–4.66)	1.02 (0.85–1.18)
Middle East and North Africa—WB	2546 (2017–3076)	3.73 (2.88–4.5)	8457 (6919–9558)	4.04 (3.24–4.58)	0.3 (0.26–0.34)
Minimal Health System	1049 (625–1451)	3.35 (2.01–4.62)	2551 (1722–3516)	3.97 (2.76–5.34)	0.61 (0.55–0.67)
North Africa and Middle East	3374 (2613–4241)	3.5 (2.68–4.4)	11 182 (9280–12 594)	3.92 (3.19–4.44)	0.53 (0.34–0.72)
North America	14 545 (12 557–15 550)	3.96 (3.41–4.24)	39 069 (32 118–42 762)	5.15 (4.27–5.61)	0.79 (0.72–0.87)
Northern Africa	1167 (874–1427)	3.69 (2.65–4.57)	3723 (2940–4423)	4.44 (3.43–5.3)	0.85 (0.76–0.93)
Oceania	65 (44–82)	4.48 (3.27–5.61)	185 (132–238)	4.29 (3.16–5.46)	−0.19 (−0.25 to 0.13)
Region of the Americas	23 026 (20 355–24 387)	4.18 (3.66–4.44)	68 568 (57 542–74 531)	4.84 (4.08–5.25)	0.47 (0.41–0.53)
South-East Asia Region	10 804 (7589–15 336)	2.94 (2.11–4.14)	48 708 (38 887–57 346)	4.06 (3.24–4.76)	1.12 (0.98–1.26)
South Asia	7594 (4655–11 542)	2.38 (1.46–3.62)	36 165 (27 041–46 073)	3.69 (2.75–4.71)	1.58 (1.38–1.78)
South Asia—WB	7917 (4944–11 954)	2.41 (1.51–3.64)	37 131 (27 937–46 998)	3.69 (2.78–4.7)	1.56 (1.36–1.75)
Southeast Asia	6120 (5116–7566)	4.24 (3.5–5.3)	22 401 (18 974–25 759)	5.27 (4.39–6.12)	0.61 (0.49–0.74)
Southern Africa	800 (606–981)	3.03 (2.32–3.75)	2285 (1893–2648)	4.17 (3.49–4.69)	1.05 (0.86–1.24)
Southern Latin America	1317 (1195–1407)	3.61 (3.22–3.88)	3299 (2853–3562)	3.53 (3.06–3.81)	0.74 (0.38–1.11)
Southern Sub-Saharan Africa	511 (417–626)	2.82 (2.26–3.48)	1413 (1228–1551)	4 (3.39–4.44)	1.14 (0.82–1.46)
Sub-Saharan Africa—WB	4511 (3165–5765)	3.83 (2.73–4.89)	10 920 (8703–13 237)	4.02 (3.21–4.81)	0.03 (−0.04 to 0.1)
Tropical Latin America	2727 (2419–2896)	4.99 (4.29–5.36)	11 540 (9640–12 679)	4.86 (4.05–5.34)	−0.12 (−0.21 to 0.03)
Western Africa	1923 (1442–2486)	4.54 (3.41–5.87)	4054 (3231–4671)	4.42 (3.57–5.06)	−0.3 (−0.4 to 0.21)
Western Europe	31 335 (27 682–33 261)	5.43 (4.75–5.77)	72 184 (58 846–79 292)	5.52 (4.56–6.04)	0.31 (0.24–0.38)
Western Pacific Region	25 237 (21 449–29 624)	4.2 (3.49–4.9)	94 170 (76 497–109 529)	3.71 (3–4.33)	−0.61 (−0.7 to 0.51)
Western Sub-Saharan Africa	2121 (1585–2724)	4.51 (3.37–5.78)	4568 (3662–5235)	4.46 (3.65–5.13)	−0.23 (−0.32 to 0.14)
World Bank High Income	58 610 (51 826–61 993)	4.74 (4.16–5.04)	146 131 (119 839–160 027)	4.7 (3.9–5.12)	0.04 (0–0.09)
World Bank Low Income	2540 (1612–3365)	3.41 (2.17–4.45)	6431 (4676–8594)	3.47 (2.55–4.65)	0.09 (−0.01 to 0.2)
World Bank Lower Middle Income	18 921 (14 539–24 851)	3.25 (2.54–4.23)	68 467 (56 967–78 231)	4.29 (3.55–4.91)	0.94 (0.85–1.03)
World Bank Upper Middle Income	34 293 (29 814–39 106)	4.23 (3.62–4.81)	117 545 (97 795–133 528)	4.12 (3.41–4.7)	−0.19 (−0.29 to 0.1)

ASDR, age-standardized death rate; CI, confidence interval; EAPC, estimated annual percentage change; SDI, socio-demographic index; UI, uncertainty interval.

**Table 4:** The DALYs cases and age-standardized DALYs of AF/AFL in 1990 and 2021, and its temporal trends from 1990 to 2021, by sex, SDI quintile, and GBD region

Characteristics	Number of DALYs cases in 1990	ASDAR per 100 000 (95% UI)	Number of DALYs cases in 2021	ASDAR per 100 000 (95% UI)	1990–2021 EAPC (95% CI)
Global	3 358 708 (2 715 430–4 141 735)	100.81 (82.82–122.62)	8 358 894 (6 970 688–10 133 489)	101.4 (84.89–122.41)	0 (−0.02 to 0.02)
Sex					
Female	1 788 781 (1 483 399–2 202 805)	93.29 (77.72–113.71)	4 326 773 (3 603 522–5 211 317)	92.24 (76.84–111.24)	−0.1 (−0.12 to 0.07)
Male	1 569 927 (1 231 979–1 964 377)	109.93 (88.5–134.72)	4 032 121 (3 310 673–4 901 830)	112.05 (93.3–135.28)	0.09 (0.08–0.11)
Age					
30–34 years	6240 (4700–8429)	1.62 (1.22–2.19)	10 078 (7925–13 319)	1.67 (1.31–2.2)	0.12 (0.05–0.19)
35–39 years	18 994 (11 451–31 608)	5.39 (3.25–8.97)	30 885 (19 367–49 682)	5.51 (3.45–8.86)	0.1 (0.03–0.16)
40–44 years	43 324 (28 002–67 491)	15.12 (9.77–23.56)	76 383 (51 014–116 783)	15.27 (10.2–23.34)	0.06 (−0.01 to 0.12)
45–49 years	77 141 (52 671–110 825)	33.22 (22.68–47.73)	158 384 (106 687–221 258)	33.45 (22.53–46.73)	0.08 (0.01–0.14)
50–54 years	136 433 (95 104–189 124)	64.18 (44.74–88.97)	286 618 (194 177–396 623)	64.42 (43.64–89.14)	0.06 (0.01–0.11)
55–59 years	212 173 (151 047–286 888)	114.56 (81.56–154.91)	463 951 (331 198–623 477)	117.24 (83.69–157.55)	0.1 (0.07–0.13)
60–64 years	309 184 (224 654–422 473)	192.51 (139.88–263.04)	628 936 (461 139–846 124)	196.51 (144.08–264.37)	0.07 (0.05–0.1)
65–69 years	390 230 (291 341–516 754)	315.7 (235.69–418.05)	871 575 (659 243–1 149 176)	315.97 (238.99–416.61)	−0.01 (−0.03 to 0.02)
70–74 years	456 083 (349 162–609 031)	538.72 (412.42–719.38)	1 124 193 (883 395–1 448 890)	546.15 (429.17–703.89)	−0.05 (−0.08 to 0.02)
75–79 years	553 399 (435 371–714 639)	899.02 (707.28–1160.97)	1 159 413 (922 654–1 463 210)	879.11 (699.59–1109.46)	−0.09 (−0.12 to 0.06)
80–84 years	556 332 (459 921–686 291)	1572.63 (1300.1–1939.99)	1 359 428 (1 140 384–1 636 165)	1552.16 (1302.06–1868.13)	−0.08 (−0.11 to 0.05)
85–89 years	366 609 (312 103–432 859)	2426.09 (2065.39–2864.52)	1 109 713 (941 751–1 289 736)	2427.1 (2059.74–2820.83)	0.02 (−0.01 to 0.05)
90–94 years	178 989 (151 780–205 234)	4176.92 (3541.96–4789.39)	776 007 (638 257–886 607)	4337.81 (3567.8–4956.05)	0.12 (0.08–0.16)
95+ years	53 578 (43 088–61 616)	5262.58 (4232.28–6052.16)	303 330 (235 930–347 976)	5565.37 (4328.74–6384.52)	0.09 (−0.02 to 0.2)
SDI region					
High SDI	1 327 542 (1 075 909–1 630 068)	119.83 (97.47–146.18)	2 788 952 (2 345 598–3 317 970)	118.88 (99.51–141.23)	−0.05 (−0.09 to 0.01)
High-middle SDI	827 405 (668 171–1 021 693)	98.39 (80.58–119.12)	1 871 378 (1 545 511–2 273 392)	96.58 (79.91–116.81)	−0.07 (−0.11 to 0.03)
Middle SDI	703 287 (554 668–874 920)	92.91 (76.32–114.46)	2 256 889 (1 828 561–2 776 292)	96.28 (79.25–117.4)	0.06 (0.02–0.1)
Low-middle SDI	371 209 (283 400–482 922)	80.09 (61.96–102.79)	1 110 930 (893 165–1 385 764)	94.25 (76.45–116.71)	0.55 (0.52–0.59)
Low SDI	124 604 (87 734–165 979)	75 (53.23–99.5)	321 963 (242 299–416 819)	84.52 (63.65–108.24)	0.48 (0.36–0.6)
GBD region					
Africa	163 182 (123 858–207 094)	79.38 (61.31–99.5)	404 602 (318 667–504 266)	84.82 (67.77–104.12)	0.18 (0.15–0.21)
African Region	133 067 (100 998–169 877)	81.97 (63–103.59)	324 559 (252 516–407 039)	86.78 (69.28–107.31)	0.13 (0.1–0.16)
America	691 298 (545 730–866 793)	117.14 (92.96–146.06)	1 766 101 (1 478 201–2 089 733)	128.09 (107.1–151.67)	0.28 (0.26–0.31)
Andean Latin America	19 240 (15 208–23 493)	106.95 (85.46–130.46)	56 583 (44 608–69 532)	99.93 (78.68–122.31)	−0.27 (−0.36 to 0.19)
Asia	1 296 203 (1 017 915–1 633 974)	85.07 (68.74–106.21)	4 026 948 (3 269 416–4 982 293)	87.96 (72.78–107.27)	0.05 (0.02–0.08)
Australasia	34 608 (29 287–40 036)	153.78 (131.23–177.04)	87 583 (72 120–105 463)	147.83 (121.51–179.59)	0 (−0.07 to 0.07)
Advanced Health System	1 854 832 (1 508 861–2 283 157)	115.61 (94.58–141.19)	3 690 884 (3 110 092–4 376 794)	116.21 (97.22–137.87)	0 (−0.03 to 0.03)
Basic Health System	1 016 078 (811 735–1 259 265)	93.24 (76.88–113.64)	3 167 936 (2 574 717–3 904 616)	95.63 (79.01–115.9)	0.04 (−0.02 to 0.09)
Caribbean	28 429 (23 264–34 542)	122.84 (102.16–147.29)	63 053 (51 870–76 838)	115.24 (94.57–140.74)	−0.19 (−0.22 to 0.15)
Central Africa	17 554 (12 646–22 862)	87.29 (63.15–113.72)	43 687 (32 238–58 266)	93.55 (71.25–124.63)	0.16 (0.08–0.24)
Central Asia	27 964 (21 497–36 702)	66.28 (51.57–86.71)	52 600 (41 314–67 337)	75 (59.9–95.5)	0.36 (0.27–0.45)
Central Europe	157 482 (132 449–188 984)	115.47 (98.44–136.8)	265 917 (225 064–316 547)	112.31 (94.56–134.27)	−0.11 (−0.25 to 0.02)
Central Latin America	80 232 (64 872–98 986)	115.99 (95.04–141.04)	269 931 (218 012–327 388)	113.57 (91.93–137.77)	−0.08 (−0.1 to 0.06)
Central Sub-Saharan Africa	14 316 (10 216–19 085)	91.59 (64.75–123.72)	37 999 (27 202–52 014)	98.45 (70.85–138.18)	0.19 (0.09–0.3)
Commonwealth High Income	199 845 (166 764–238 685)	130.07 (108.72–154.75)	375 501 (311 939–451 518)	125.14 (103.2–150.96)	−0.1 (−0.17 to 0.03)
Commonwealth Low Income	52 932 (37 002–69 847)	77.12 (52.16–101.33)	165 837 (125 868–221 627)	92.11 (70.84–122.05)	0.49 (0.41–0.57)
Commonwealth Middle Income	349 283 (261 859–462 726)	74.73 (57.14–98.18)	1 126 001 (871 329–1 421 471)	88.47 (69.27–110.18)	0.61 (0.53–0.69)
East Asia	533 837 (416 978–668 275)	93.83 (76.48–115.88)	1 723 468 (1 364 785–2 143 575)	89.83 (72.42–109.59)	−0.23 (−0.32 to 0.13)
East Asia and Pacific—WB	919 649 (738 620–1 141 882)	91.82 (75.43–112.52)	2 770 456 (2 258 354–3 426 063)	88.39 (72.47–107.95)	−0.22 (−0.27 to 0.18)
Eastern Africa	39 615 (28 015–51 748)	75.37 (52.9–97.44)	95 993 (71 329–126 402)	77.44 (57.33–101.91)	0.03 (−0.06 to 0.11)
Eastern Europe	242 292 (195 940–303 226)	95.36 (78.74–118)	388 580 (318 353–470 379)	107.73 (88.25–130.33)	0.36 (0.27–0.44)
Eastern Mediterranean Region	100 324 (76 902–127 934)	73.72 (56.63–93.59)	279 137 (229 154–335 913)	83.39 (68.66–99.79)	0.35 (0.31–0.39)
Eastern Sub-Saharan Africa	42 708 (29 866–56 564)	75.84 (52–99.79)	103 373 (74 839–140 257)	79.25 (57.05–108.27)	0.1 (0.01–0.19)
Europe	1 198 852 (987 948–1 467 361)	118.48 (98.39–143.79)	2 143 013 (1 803 191–2 528 795)	120.68 (100.81–143.89)	0.09 (0.04–0.14)
Europe and Central Asia—WB	1 214 570 (1 000 558–1 488 285)	117.02 (97.18–142.14)	2 172 465 (1 826 634–2 567 749)	119.45 (99.73–142.53)	0.1 (0.04–0.15)
European Region	1 223 481 (1 007 878–1 499 361)	117.11 (97.25–142.26)	2 196 895 (1 847 967–2 596 531)	119.67 (99.9–142.83)	0.1 (0.05–0.15)
High-income Asia Pacific	156 515 (125 660–193 831)	83.08 (67.38–102.03)	364 512 (301 067–443 767)	69.03 (56.4–84.74)	−0.84 (−1.05 to 0.63)
High-income North America	443 449 (346 576–559 226)	121.41 (94.78–152.92)	1 015 978 (854 240–1 202 900)	144.37 (121–171.39)	0.54 (0.5–0.58)
Latin America and Caribbean—WB	252 238 (202 086–312 391)	111.77 (91.56–136.53)	759 213 (621 128–925 884)	110.77 (90.75–135)	0.01 (−0.01 to 0.03)
Limited Health System	450 177 (334 989–591 416)	76.45 (57.46–100.26)	1 417 153 (1 105 985–1 794 958)	89.2 (69.89–111.71)	0.54 (0.47–0.61)
Middle East and North Africa—WB	68 089 (54 317–84 311)	76.79 (61.85–93.96)	214 656 (175 101–253 240)	82.45 (68.2–97.11)	0.25 (0.23–0.27)
Minimal Health System	32 96 (23 155–43 170)	74.94 (52.14–99.31)	74 138 (53 922–97 881)	83.45 (61.87–109.17)	0.37 (0.33–0.41)
North Africa and Middle East	88 830 (69 529–110 179)	71.6 (56.5–88.83)	265 649 (218 559–309 833)	76.1 (63.33–88.63)	0.23 (0.12–0.33)
North America	443 458 (346 583–559 237)	121.4 (94.78–152.91)	1 016 018 (854 266–1 202 948)	144.36 (120.99–171.37)	0.54 (0.5–0.58)
Northern Africa	31 512 (24 263–39 022)	73.13 (57.14–90.01)	91 381 (73 526–109 865)	82.98 (66.66–99.61)	0.52 (0.48–0.56)
Oceania	2441 (1825–3093)	110.29 (85.01–137.38)	6606 (5044–8584)	110.15 (86.19–141.7)	−0.02 (−0.05 to 0)
Region of the Americas	691 298 (545 730–866 793)	117.14 (92.96–146.06)	1 766 101 (1 478 201–2 089 733)	128.09 (107.1–151.67)	0.28 (0.26–0.31)
South-East Asia Region	417 493 (317 065–554 526)	80.36 (61.52–105.39)	1 411 590 (1 109 818–1 780 709)	94.51 (75.57–117.08)	0.56 (0.5–0.61)
South Asia	311 117 (228 511–416 726)	71.78 (52.92–96.69)	1 071 260 (820 127–1 366 680)	88.3 (68.02–111.45)	0.74 (0.66–0.82)
South Asia—WB	322 015 (236 399–431 212)	72.05 (53.39–97.03)	1 099 152 (842 561–1 399 533)	88.37 (68.14–111.44)	0.73 (0.65–0.81)
Southeast Asia	200 296 (158 700–252 961)	102.35 (82.98–128.37)	612 969 (501 403–745 497)	115.4 (95.88–139.11)	0.34 (0.27–0.41)
Southern Africa	26 546 (20 762–33 713)	76.59 (60.7–96.62)	67 819 (54 307–84 419)	92.52 (75.8–112.25)	0.63 (0.53–0.74)
Southern Latin America	34 600 (28 133–42 981)	82.4 (67.73–101.03)	67 844 (58 167–78 537)	74.67 (63.84–86.8)	0.14 (−0.08 to 0.36)
Southern Sub-Saharan Africa	17 046 (13 347–21 744)	75.25 (59.56–95.82)	41 787 (34 114–50 581)	90.4 (74.56–108.99)	0.62 (0.44–0.8)
Sub-Saharan Africa—WB	132 276 (100 035–169 517)	81.17 (62.05–103.01)	314 448 (244 803–395 915)	85.46 (67.58–105.82)	0.1 (0.06–0.13)
Tropical Latin America	90 777 (71 331–113 772)	122.82 (99.71–151.03)	304 099 (245 416–375 972)	122.75 (99.62–151.65)	−0.04 (−0.09 to 0)
Western Africa	47 954 (37 436–60 883)	84.55 (66.61–106.13)	105 722 (82 182–130 285)	85.51 (67.49–103.77)	−0.08 (−0.13 to 0.02)
Western Europe	779 577 (634 739–949 783)	130.74 (106.98–159.03)	1 440 284 (1 208 224–1 709 735)	131.15 (108.91–156.67)	0.08 (0.03–0.12)
Western Pacific Region	770 687 (616 064–954 600)	89.58 (73.54–109.4)	2 321 265 (1 884 305–2 862 897)	84.93 (69.3–103.8)	−0.27 (−0.32 to 0.21)
Western Sub-Saharan Africa	52 952 (41 258–67 156)	84.18 (66.13–105.66)	118 819 (92 666–146 393)	86.12 (68.4–104.09)	−0.03 (−0.08 to 0.01)
World Bank High Income	1 565 655 (1 267 474–1 925 064)	120.87 (98.19–147.64)	3 207 228 (2 700 060–3 814 655)	119.83 (100.24–142.58)	−0.04 (−0.06 to 0.01)
World Bank Low Income	81 572 (58 834–105 441)	76.39 (55.06–98.05)	195 087 (148 558–251 095)	78.76 (61.15–101.4)	0.1 (0.04–0.16)
World Bank Lower Middle Income	648 511 (502 187–837 064)	82.29 (64.97–104.65)	1 925 763 (1 541 040–2 393 244)	96 (77.68–117.4)	0.51 (0.48–0.55)
World Bank Upper Middle Income	1 052 444 (844 931–1 303 925)	92.23 (76.46–111.67)	3 014 727 (2 451 365–3 720 467)	93.03 (77.02–113.01)	−0.03 (−0.08 to 0.02)

ASDAR, age-standardized DALYs rate; CI, confidence interval; EAPC, estimated annual percentage change; SDI, socio-demographic index; UI, uncertainty interval.

From 1990 to 2021, the incidence, prevalence, deaths, and DALYs for AF/AFL increased for both sexes. Males had a slightly higher disease burden than females, with their prevalence and incidence exceeding women post-2000. However, females consistently had higher death and DALY counts. Age-standardized rate analysis showed male ASRs for prevalence, incidence, and DALYs surpassed females’ throughout the period. Conversely, before 2005, female ASR for death exceeded males’, but since 2005, male ASR for death has increased faster (see Supplementary material online, *Figure S2*).

Age-standardized rates and case counts for AF/AFL incidence, DALYs, prevalence, and death were higher in upper SDI quintiles from 1990 to 2021. Low–middle SDI regions had higher ASRs for incidence than high–middle and middle SDI regions. High SDI quintile consistently had the highest values. Trends in high–middle and middle SDI regions were similar (see Supplementary material online, *Figure S3*; *Tables 1–4*). Cluster analysis revealed distinct patterns, with Southern Latin America showing a significant increase. Thirty-eight regions, including World Bank High Income, fell into the minor increase category. Fourteen regions, such as World Bank Lower Middle Income, remained stable or had minor decreases. High-income Asia Pacific showed a significant decrease (see Supplementary material online, *Figure S4*).

**Figure 2:**
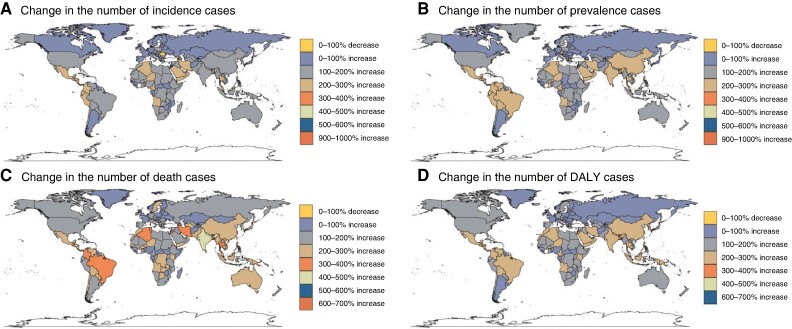
Change in numbers of incidence, prevalence, deaths, and DALYs for AF/AFL patients across 204 countries and territories for both sexes from 1990 to 2021, AF, atrial fibrillation; AFL, atrial flutter; DAYLs, disability-adjusted life years.

**Figure 3:**
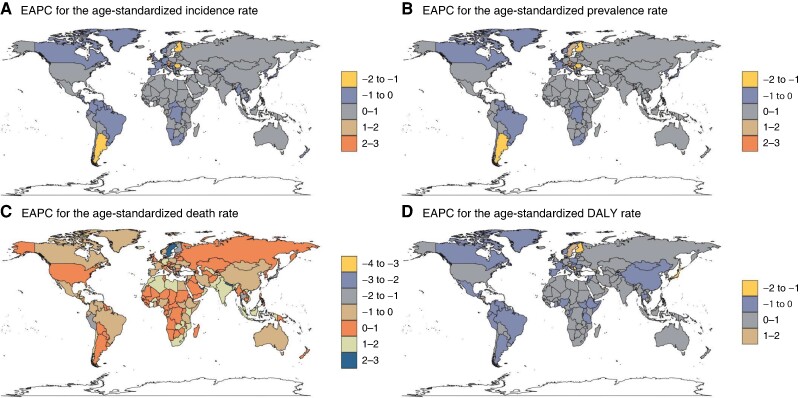
The EAPC of AF/AFL in 204 countries and territories between 1990 and 2019, AF, atrial fibrillation; AFL, atrial flutter; EAPC, estimated annual percentage change.

From 1990 to 2021, the United Arab Emirates (971% increase), Qatar (907%), and Jordan (971%) had the highest increases in AF/AFL incidence cases. These three countries also showed the greatest prevalence increases, with the UAE (942%), Qatar (908%), and Jordan (557%). For mortality, Kuwait (606%), Maldives (590%), and Bhutan (571%) had the highest death increases. In terms of DALYs, the UAE (660%), Qatar (557%), and Kuwait (460%) showed the most substantial increases. Romania (−6%), Georgia (−4%), and Niue (−2%) had the largest decreases in incidence cases. Niue uniquely showed decreasing trends in prevalence (−1%), mortality (−24%), and DALYs (−12%) (*Figure 2*). Using the EAPC, Austria (2.21), Czechia (1.50), and Israel (1.25) showed the highest incidence growth rates, while Argentina (−1.31), Finland (−1.16), and Romania (−1.21) had the largest decreases. For prevalence, Austria (2.33), Israel (1.46), and Czechia (1.44) led growth, and Romania (−1.17), Finland (−1.12), and Argentina (−1.03) showed the greatest reductions. In mortality, the United Arab Emirates (2.97), Sweden (2.57), and Lesotho (2.28) had the highest growth; Guam (−3.00), Qatar (−2.71), and Saint Lucia (−2) decreased the most. For DALYs, the United Arab Emirates (1.96), Sweden (1.61), and Austria (1.46) grew the most, while Cyprus (−1.71), Qatar (−1.66), and Finland (−1.47) decreased most (*Figure 3*; Supplementary material online, *Tables S1–S4*).

### Global age distribution of atrial fibrillation/atrial flutter disease burden in 2021

Worldwide, the highest incidence and prevalence of AF were observed in the 70–74 age group for both sexes. However, when considering age-specific incidence rates, both sexes exhibited peak values in the 95+ age group (see Supplementary material online, *Figure S5*; *Tables 1* and *2*). Mortality due to AF peaked in the 75–79 age group for both sexes. In contrast, the global number of DALYs peaked in the 85–89 age group for males and the 80–84 age group for females. Nonetheless, the age-specific rates of mortality and DALYs for both sexes still peaked in the 95+ age group (see Supplementary material online, *Figure S5*; *Tables 3* and *4*). Notably, the global number of deaths and DALYs attributed to AF was lower among males compared with females across all age groups. However, other indicators, such as those depicted in Supplementary material online, *Figure S6* and *Tables 1–4*, exhibited greater values in males compared with females across the entire age range.

In 2021, the high SDI region had higher incidence, prevalence, mortality, and DALYs than other regions, regardless of ASR or absolute cases. As SDI decreased, ASRs declined gradually. However, middle SDI countries had higher absolute case numbers than high–middle SDI countries (see Supplementary material online, *Figure S7*; *Tables 1–4*). Across 204 countries, a positive correlation was found between SDI and ASRs of incidence, prevalence, mortality, and DALYs. Some countries showed significantly higher ASRs than expected based on their SDI values (see Supplementary material online, *Figure S8*).

Among 54 GBD regions, Asia, Basic Health System, and Advanced Health System had the highest incidence cases, with similar trends for prevalence, mortality, and DALYs (see Supplementary material online, *Figure S9*; *Tables 1–4*). However, ASRs varied. High-income North America, North America, and the Region of the Americas had the highest incidence ASRs. Australasia, the Region of the Americas, and America led in prevalence ASRs. Australasia, Western Europe, and Commonwealth high-income regions had the highest death ASRs. Andean Latin America, Central Sub-Saharan Africa, and World Bank Lower Middle Income regions showed the highest DALYs ASRs (*Figure S9*; *Tables 1–4*).

**Figure 4:**
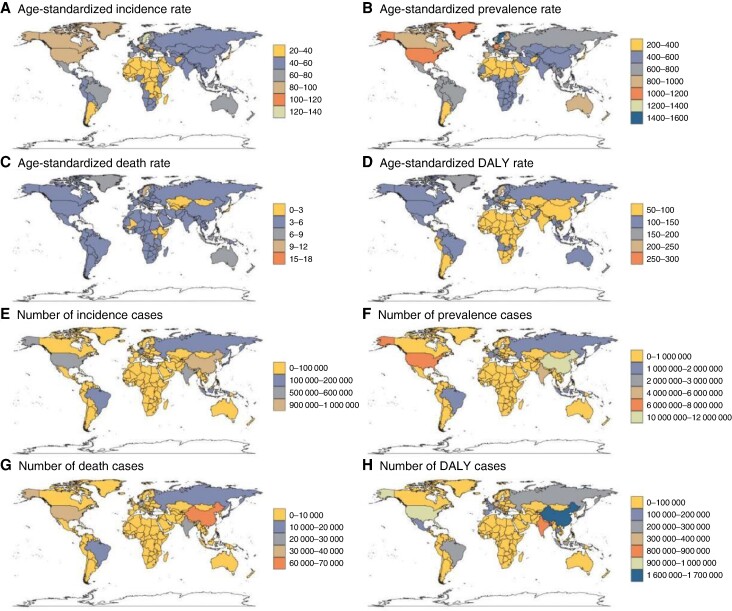
Age-standardized rate and numbers of incidence, prevalence, deaths, and DALYs of AF/AFL in 204 countries and territories in 2021, AF, atrial fibrillation; AFL, atrial flutter; DAYLs, disability-adjusted life years.

Globally, China, India, and the USA have the highest incidence, prevalence, and DALYs. However, for mortality, China, India, and Germany have the most deaths. In terms of ASRs, Sweden, Austria, and Germany lead in incidence and prevalence, while Montenegro, Nauru, and Sweden have the highest death and DALYs ASRs (*Figure 4*; Supplementary material online, *Tables S1*–*S4*).

The EAPC was significantly associated with the ASRs of incidence, prevalence, and deaths, but not with the ASR of DALYs. Specifically, when the ASRs surpassed 75/100 000 for incidence, 800/100 000 for prevalence, and 5/100 000 for deaths, a positive correlation with EAPC was observed. However, the EAPC of deaths and DALYs in middle HDI regions was higher, forming an inverted U-shape pattern. Conversely, the EAPC for incidence and prevalence showed a slight downward trend with increasing HDI. Overall, higher HDI countries tend to experience a decline in AF/AFL-related ASRs (see Supplementary material online, *Figure S10*).

### Attributable burden of atrial fibrillation to risk factors

The GBD 2021 classifies risk factors into three groups: environmental/occupational, behavioural, and metabolic. Among them, metabolic risks contribute the most to attributable risks for ASRs, deaths, and DALYs in 1990 and 2021, followed by behavioural risks and then environmental/occupational risks (see Supplementary material online, *Figure S11*). Subgroup analysis shows that high systolic blood pressure is the leading risk factor for ASR, DALYs, and deaths in both years. In regions with higher SDI, high BMI is the second most prevalent risk factor after high systolic blood pressure (see Supplementary material online, *Figure S12* and *Table S5*). In lower SDI regions, other environmental risks rank second after high systolic blood pressure. Among subgroups, smoking had the most significant impact on deaths and age-standardized death rates (ASDRs) in 2021, accounting for 35% and 34.4%, respectively. The disparity with high-sodium diet and lead exposure was minor. Similarly, smoking contributed most to DALYs and age-standardized DALYs rates, standing out at 43.9% and 43.3%. In high–middle and middle SDI regions, a high-sodium diet contributed marginally more to AF/AFL deaths than smoking in 2021 (see Supplementary material online, *Figure S13*).

## Discussion

This study offers a comprehensive epidemiological analysis of AF/AFL's global incidence, prevalence, mortality, and DALYs. Our results show AF/AFL is a major global health issue, with 1.37-fold prevalence and 1.24-fold incidence increases over 31 years. In 2021, there were 4.48 million new cases, 52.55 million prevalent cases, 338 947 deaths, and 8 358 894 DALYs. Despite declining ASRs, absolute cases doubled due to population growth, aging, improved diagnostics, and increased awareness. Notably, DALYs and deaths are increasing faster than prevalent and incident cases, and the age-standardized mortality rate is the only upward-trending indicator. This underscores the need for early detection and urgent development of more effective AF/AFL management strategies.

Our findings align with the GBD 2019 study, indicating a higher burden of DALYs and deaths due to AF/AFL among females compared with males in 2021. Conversely, the incidence and prevalence trends differ. Women experience higher morbidity and complications related to AF/AFL,^19–21^ likely due to underutilization of rhythm control strategies and less oral anticoagulant treatment.^22,23^ This contributes to poor prognosis in the female population. An analysis of AF-related mortality in Europe from 2008 to 2019 revealed a higher total number of deaths in women despite a greater increase in age-standardized mortality for men. This suggests potential inequalities in medical care or biological differences between genders.^24^ Hence, prioritizing rhythm control techniques and complication management for female AF/AFL patients is crucial.

The long-standing Framingham Heart Study emphasizes aging as the paramount risk for AF, surpassing other factors.^25^ Aging is linked to oxidative stress, mitochondrial dysfunction, cardiomyocyte hypertrophy, and ion channel inactivation, fostering atrial structural and electrical remodelling, heightening AF risk.^26,27^ Our study reveals a steep rise in AF/AFL incidence, prevalence, deaths, and DALYs among the elderly, with an increasing trend in ASDR and DALYs. This underscores the urgency for improved AF/AFL management in the elderly.

Analogous to the GBD 2019 analysis of AF/AFL, considerable variations in the ASRs and case numbers of incidence, prevalence, deaths, and DALYs were observed across different SDI quintiles. The SDI, comprising years of education, per capita income, and total fertility rate, significantly impacts these metrics and their temporal changes in AF/AFL. Consequently, each region must devise tailored solutions grounded in its unique socio-economic context. Regions with high SDI exhibited higher ASIRs and case numbers, whereas low SDI regions had lower rates and case numbers. This suggests that a higher percentage of individuals in low SDI locations may have undetected AF/AFL. However, in high SDI regions, a greater number of AF/AFL patients, particularly those with paroxysmal or asymptomatic AF/AFL, are being identified at an early stage with high accuracy. The EAPC values for age-standardized incidence, death, and DALY rates in low SDI areas are positive, indicating an increasing trend during the study period. Conversely, high SDI areas showed a decreasing trend, despite having higher incidence and death cases. This suggests that the overall medical treatment effect for AF/AFL is superior in high SDI regions. In general, the burden of AF/AFL was higher in high SDI regions than in low SDI regions, which is similar to the findings of a study examining the impact of socio-economic factors on trends in AF incidence and mortality in Europe from 1990 to 2017.^28^ The observed phenomenon might stem from disparities in the application of therapeutic strategies, encompassing the utilization of novel oral anticoagulants, left atrial appendage closure procedures, and catheter-based or cryogenic balloon ablation techniques. These interventions are not uniformly accessible across diverse socio-economic and geographical landscapes. While significant progress has been made in addressing the AF/AFL burden in high SDI areas in recent years, there has been a notable oversight in managing AF/AFL in low SDI areas. We speculate that because of the better medical conditions in high SDI areas, AF/AFL patients can survive long enough and may develop more comorbidities or worsen their AF/AFL condition. Hence, there is a pressing need to bolster efforts towards the prevention, diagnosis, and treatment of AF/AFL in low SDI regions in the future.

It is worth noting that in some studies, because the death data used come from relevant publicly accessible World Health Organization databases, there are certain differences from our research. For example, in the above-mentioned analysis study on the trend of AF-related mortality in Europe from 2008 to 2019,^24^ the study believed that any European region has a certain increase in the age-standardized mortality rate of AF and the main increase is concentrated in Eastern European countries. Our study believes that the occurrence and development of national age-standardized mortality rates in the European region are inconsistent and the increase mainly occurs in economically developed areas in Western Europe. The reason for this difference is that the death data in the World Health Organization database and the GBD database have different sources and different adjustment methods.^24^

The current study reveals substantial geographic heterogeneity in AF burden. High-income North America showed the highest ASIR, while Australasia led in ASDR and ASPR. Across 204 countries, the ASIR, ASDR, and age-standardized DALY rate positively correlated with SDI, indicating a heavier AF burden in higher SDI countries. This aligns with prior research.^29–31^ Country-level variations in AF burden may be due to disparities in aetiology, detection, ethnicity, health resources, and treatment. Better medical treatment and public awareness in high SDI regions likely contribute to increased AF identification and reporting. However, data collection limitations in regions without robust medical systems may underestimate the overall AF burden. Among AF patients, a significant subpopulation comprises asymptomatic individuals. Research suggests that ∼15–50% of clinical AF patients exhibit asymptomatic manifestations, varying with the study population, AF type (paroxysmal, persistent, or permanent), and detection method.^32,33^ Notably, fewer asymptomatic AF patients are women, likely due to women's tendency to promptly seek medical attention for elevated heart rates.^34^ While rhythm control strategies, such as antiarrhythmic drugs, catheter ablation, and electrical cardioversion, are predominantly used in symptomatic AF patients, their utilization in asymptomatic patients is less frequent. However, asymptomatic AF patients often face a higher stroke risk due to inadequate evaluation of anticoagulant use.^35^ This has sparked controversy regarding the relationship between asymptomatic AF and prognosis, as previously, asymptomatic AF was considered benign, with symptom control being the primary treatment goal.^36^ Recent studies, including Boriani *et al*.'s work,^37^ have demonstrated that asymptomatic AF patients exhibit a two-fold higher 1-year mortality compared with symptomatic patients. Additionally, a Chinese cross-sectional study found asymptomatic AF patients to have a higher incidence of clinical events leading to hospitalization.^38^ Further clinical investigations are imperative to explore the characteristics of this subpopulation and devise effective management strategies.

Literature reviews suggest that managing modifiable factors like hypertension, obesity, smoking, and excessive alcohol consumption can prevent AF/AFL and improve prognosis.^39,40^ This study analysed modifiable risk factors contributing to AF/AFL cases and rates, including high systolic blood pressure, BMI, alcohol, smoking, dietary risks (high sodium), and environmental risks (lead exposure). High systolic blood pressure is the primary risk factor, with a 10 mmHg increase elevating AF risk by 19%.^41^ Effective blood pressure control significantly reduces AF/AFL incidence.^42,43^ The second most significant risk factor is a high BMI, with each unit increase associated with a 4–5% rise in AF risk.^44^ This aligns with the Western diet of high calories, fat, and sugar. Smoking's attributable share in AF/AFL is declining due to tobacco control and health promotion.^45^ Lead exposure, although rarely linked to AF directly, contributes to hypertension and cardiovascular disease, both risk factors for AF/AFL.^46^ Managing risk factors like smoking cessation, alcohol abstinence, low-salt/fat diet, exercise, and healthy lifestyle practices can reduce AF/AFL incidence. However, tailored prevention strategies are needed considering the varying risk factors across different socio-economic levels.

In assessing AF risk factors, the impact of the COVID-19 pandemic, which has had worldwide prevalence in recent years, must be considered. A clinical study found a significant 14.8% occurrence of AF in COVID-19 patients, accompanied by a heightened risk of mortality, ischaemic stroke, and cardiac arrest. This indicates that not only is AF more prevalent in COVID-19 patients but it also independently correlates with a higher mortality rate after adjusting for confounders.^47^ A survey conducted by the Italian Association of Arrhythmology and Cardiac Pacing revealed that during the COVID-19 pandemic in Italy, radiofrequency ablation surgeries for arrhythmias decreased by 70% during the first wave (March to May 2020), with incomplete recovery in subsequent periods compared with other surgeries. This suggests that inadequate AF evaluation and treatment during the pandemic contributed to the global AF mortality rate during this time.^48^ A nationwide US study of patients under remote monitoring during the early COVID-19 pandemic (21 January to 29 April 2020) found a significant association between COVID-19 outbreaks and AF prevalence. States with higher COVID-19 infection rates had a more significant rise in AF, likely due to social unrest affecting medication adherence, dietary and behavioural patterns, and negative emotions.^49^ Meta-analysis revealed common risk factors between AF and COVID-19, suggesting a close link between AF and infectious diseases due to oxidative stress, inflammatory mediators, and haemodynamic disorders.^50^ Notably, even recovered COVID-19 patients showed a higher incidence of AF compared with uninfected patients during the same follow-up period, emphasizing the need for suspicion of AF among recovered COVID-19 patients with relevant symptoms.^51^

Beyond COVID-19 and lung inflammation, numerous other risk factors, including cardiomyopathy, obstructive sleep apnoea syndrome, coronary artery disease, and lung disease, contribute to the incidence and mortality of AF, yet are not fully captured in the GBD database. Cardiomyopathy, particularly hypertrophic cardiomyopathy, is a prominent example. This condition often leads to AF due to increased left atrial pressure and atrial myopathy resulting from unexplained left ventricular hypertrophy and outflow tract obstruction.^52^ The prevalence of AF in hypertrophic cardiomyopathy patients, when age-matched, is significantly higher (20–30%) with an annual incidence of 2–4%, far exceeding the general population rates.^53^ Moreover, patients with hypertrophic cardiomyopathy have a lower tolerance to AF, and those with AF face a notably higher risk of embolic events, aberrant ICD discharges, and all-cause mortality.^54^ The interplay between coronary heart disease (CHD) and AF is intricate, as the two conditions can coexist. Atrial fibrillation can exacerbate inflammatory responses, hastening atherosclerosis, while CHD heightens the risk of AF by enhancing focal ectopic activity in the left atrium and altering micro-re-entry patterns.^55^ Prior studies reveal that CHD prevalence in AF patients ranges from 18% to 46.5%, acting as an independent risk factor for AF development. Moreover, AF frequently coincides with non-ST-segment elevation myocardial infarction (NSTEMI), with AF patients exhibiting a 39% higher risk of NSTEMI and double the likelihood of cardiovascular events or mortality compared with non-AF patients.^56,57^

Global treatment strategies have shifted, and AF mortality and rehospitalization are increasingly influenced by non-cardiovascular causes.^58^ Obstructive sleep apnoea syndrome contributes to higher recurrence of AF post-treatment and reduces radiofrequency ablation success, impacting mortality.^59^ A study of over 4000 AF patients in five Asian countries revealed that despite good anticoagulant use, most deaths were due to non-cardiovascular diseases (72.3%). Multivariate analysis identified obstructive pulmonary disease as an independent predictor of mortality and the primary cause of re-admission.^60^ Similarly, data from the German Atrial Fibrillation Network highlight chronic obstructive lung disease as a contributor to higher mortality rates among AF patients.^61^

Heart failure, a terminal cardiovascular disease outcome, profoundly influences AF epidemiology. A Danish study showed that heart failure preceding AF led to a higher mortality risk than the reverse or concurrent conditions, likely due to the irreversibility of pre-existing chronic disease.^62^ A US study spanning 1999–2020 revealed a 286.4% increase in age-adjusted heart failure mortality among AF patients, with men and younger individuals (<65 years) experiencing a steeper rise. This trend is attributed to the rising prevalence of comorbidities like diabetes. Geographically, Whites and those in the Midwestern USA have higher heart failure-related mortality rates. The peak in heart failure mortality among AF patients between 2011 and 2012 is likely due to improved AF awareness and diagnostic accuracy following US guidelines issued in 2011. Anticoagulant choice in AF patients with heart failure affects mortality, as heart failure heightens anticoagulation risks and AF mortality, even with new oral anticoagulants.^63–65^

While the current findings are significant, several limitations must be acknowledged. Firstly, data bias from confounding factors is a concern, particularly in countries with incomplete death certification systems, where verbal autopsy data are often utilized despite its limitations. Additionally, despite our efforts to introduce a risk-confounding star rating system in the GBD assessment, uncertainties in measurements may still be overlooked or misunderstood. The diverse data sources with potential biases and missing data for specific locations and years further complicate the GBD analysis. In some cases, the data patterns or relative risk functions may differ from expert consensus. The GBD follows a rule-based evidence synthesis approach, which can lead to discrepancies with other assessments that rely more on expert opinion. Finally, there are two main points of the limitations for the linear regression model including under-fitting problems and sensitive to outliers.

Furthermore, the GBD studies have yet to incorporate other risk factors, such as diabetes, physical inactivity, and hyperthyroidism, into their analyses. Lastly, it is noteworthy that there exist different types of AF/AFL, including paroxysmal, persistent, and permanent AF/AFL, yet the GBD database does not recognize this complexity and merely identifies whether a population falls under the AF/AFL classification.

## Conclusion

Our study highlights the varying patterns of AF/AFL prevalence from 1990 to 2021 across gender, SDI quintiles, and regions. Despite progress in prevention and treatment in higher SDI areas, AF/AFL prevalence is rising in lower SDI regions. Given its preventable and curable nature, urgent adoption of cost-effective techniques targeting modifiable risk factors is crucial in high- or increasing-prevalence areas.

## Supplementary Material

euae195_Supplementary_Data

## Data Availability

All data generated or analysed during this study are included in this published article. The data sets generated during and/or analyses during the current study are available in the Global Burden of Disease.
