# Supplementary material for: Global burden of atrial fibrillation/atrial flutter and its attributable risk factors from 1990 to 2021
Source: Europace. 2024 Jul 10;26(7):euae195. doi: 10.1093/europace/euae195 (PMC11287210; doi:10.1093/europace/euae195)
Supplement: euae195_Supplementary_Data [file euae195_supplementary_data.zip › Table S4.docx]

Table S4 The DALYs cases and age-standardized DALYs of AF/AFL in 1990 and 2021, and its temporal trends from 1990 to 2021, by 204 countries and territories

| **Characteristics** | **Number of DALYs cases in 1990** | **ASDAR per 100,000**  **(95% UI)** | **Number of DALYs cases in 2021** | **ASDAR per 100,000**  **(95% UI)** | **1990-2021EAPC**  **(95% CI)** |
| --- | --- | --- | --- | --- | --- |
| Country |  |  |  |  |  |
| Afghanistan | 3394 (2188-4977) | 63.05 (40.48-92.63) | 5074 (3663-6761) | 72.08 (51.55-95.15) | 0.46 (0.43-0.48) |
| Albania | 1700 (1354-2122) | 97.6 (78.23-120.32) | 4399 (3431-5468) | 103.15 (81.18-126.41) | 0.39 (0.29-0.48) |
| Algeria | 6619 (5035-8537) | 90.02 (68.59-112.91) | 23845 (18698-29189) | 95.93 (76.67-116.45) | 0.63 (0.48-0.77) |
| American Samoa | 23 (18-27) | 133.26 (107.73-163.29) | 58 (45-71) | 143.68 (111.47-176.76) | 0.34 (0.31-0.37) |
| Andorra | 64 (49-85) | 125.54 (95.34-164.86) | 170 (130-217) | 102.38 (77.85-131.72) | -0.6 (-0.69--0.52) |
| Angola | 2447 (1758-3251) | 89.65 (65.44-120.75) | 8368 (6207-11093) | 102.72 (76.04-135.77) | 0.37 (0.32-0.42) |
| Antigua and Barbuda | 80 (70-95) | 139.73 (120.93-164.23) | 121 (101-147) | 132.09 (111.79-158.06) | -0.17 (-0.31--0.04) |
| Argentina | 24020 (19576-29910) | 82.39 (67.9-101.12) | 40198 (34929-46587) | 69.17 (59.96-80.23) | -0.1 (-0.31-0.12) |
| Armenia | 1451 (1074-1905) | 60.37 (45.72-79.07) | 3355 (2607-4231) | 76.87 (59.76-96.05) | 0.98 (0.81-1.15) |
| Australia | 28469 (24181-33071) | 152.15 (129.99-175.93) | 73571 (60143-89381) | 146.76 (119.81-179.53) | 0 (-0.09-0.1) |
| Austria | 14654 (12661-16940) | 119.39 (103.2-137.72) | 37096 (30927-43902) | 175.92 (146.38-208.58) | 1.46 (1.25-1.68) |
| Azerbaijan | 2729 (1994-3648) | 62.51 (46.28-83.71) | 5902 (4361-7951) | 67.92 (51.38-90.14) | 0.43 (0.35-0.51) |
| Bahamas | 177 (148-215) | 130.83 (110.46-157.54) | 469 (380-561) | 132.64 (108.37-157.69) | 0.02 (-0.1-0.13) |
| Bahrain | 101 (65-128) | 113.06 (66.97-143.09) | 442 (316-569) | 94.91 (57.64-122.79) | -0.72 (-1--0.45) |
| Bangladesh | 29954 (20406-40049) | 78.66 (52.88-105.79) | 109146 (82747-147679) | 97.3 (74.5-129.97) | 0.64 (0.46-0.82) |
| Barbados | 376 (312-456) | 123.26 (102.83-148.15) | 641 (513-774) | 123.06 (98.96-148.54) | 0.03 (-0.06-0.12) |
| Belarus | 11328 (9217-14087) | 91.19 (74.49-113.12) | 16740 (13493-20892) | 101.79 (82.13-126.55) | 0.24 (0.17-0.31) |
| Belgium | 17560 (14240-21830) | 111.27 (90.86-138.05) | 27975 (22943-33735) | 103.49 (84.22-126.61) | -0.11 (-0.24-0.03) |
| Belize | 96 (76-122) | 105.76 (83.07-134.19) | 297 (239-365) | 113.27 (91.6-138.44) | 0.11 (-0.13-0.35) |
| Benin | 1095 (824-1396) | 66.58 (50.37-84.27) | 2817 (2218-3600) | 73.63 (57.93-92.8) | 0.36 (0.32-0.39) |
| Bermuda | 76 (60-92) | 136.3 (109.71-164.1) | 160 (130-197) | 105.58 (84.94-130.79) | -0.93 (-0.99--0.87) |
| Bhutan | 123 (86-167) | 71.66 (49.36-97.91) | 503 (379-649) | 93.85 (70.8-121.02) | 0.95 (0.92-0.98) |
| Bolivia (Plurinational State of) | 3127 (2335-4175) | 121.39 (91.49-162.71) | 9440 (7098-11874) | 123.22 (93.91-154.08) | 0.1 (0.07-0.12) |
| Bosnia and Herzegovina | 3085 (2409-3907) | 89.03 (70.69-112.39) | 6410 (5131-7828) | 99.83 (80.18-121.35) | 0.46 (0.4-0.52) |
| Botswana | 310 (233-407) | 76.84 (58.18-100.28) | 862 (665-1099) | 77.73 (60.71-99.9) | 0.18 (0.05-0.32) |
| Brazil | 88346 (69433-110571) | 122.91 (99.74-151.06) | 297032 (239336-367908) | 122.58 (99.23-151.64) | -0.05 (-0.1--0.01) |
| Brunei Darussalam | 124 (102-149) | 144.66 (117.27-173.57) | 328 (271-388) | 122.03 (102.46-143.98) | -0.2 (-0.36--0.05) |
| Bulgaria | 12305 (10093-15114) | 118.85 (99.82-142.79) | 19898 (16801-23531) | 132.45 (112.78-156.09) | 0.39 (0.29-0.48) |
| Burkina Faso | 2340 (1612-3065) | 75.92 (51.64-100.43) | 6312 (4577-8276) | 93.72 (69-121.53) | 0.91 (0.82-1) |
| Burundi | 1371 (900-2021) | 71.61 (46.75-109.02) | 2555 (1694-3792) | 69.88 (45.58-107.87) | -0.26 (-0.38--0.15) |
| Cabo Verde | 175 (134-220) | 73.39 (56.36-91.97) | 387 (300-476) | 93.89 (72.94-115.1) | 0.69 (0.54-0.85) |
| Cambodia | 2927 (2223-3785) | 86.33 (66.07-113.06) | 9138 (7031-11349) | 98.48 (76.53-122.26) | 0.42 (0.35-0.49) |
| Cameroon | 2795 (2110-3447) | 90.76 (68.25-111.88) | 8308 (6392-10485) | 96.96 (75.89-120.43) | 0.13 (0.08-0.17) |
| Canada | 49511 (38763-61653) | 152.55 (120.12-189.47) | 99213 (77674-125422) | 125.65 (97.53-160.69) | -0.66 (-0.75--0.56) |
| Central African Republic | 741 (498-1034) | 93.67 (63.8-132.54) | 1396 (964-1962) | 94.52 (64.64-133.89) | 0.02 (0-0.04) |
| Chad | 1481 (1037-2001) | 64.7 (45.18-87.18) | 3120 (2274-4079) | 76.68 (56.2-97.97) | 0.55 (0.51-0.58) |
| Chile | 7594 (6160-9340) | 84.57 (69.33-103.12) | 22386 (18415-26886) | 85.2 (70.05-102.61) | 0.6 (0.29-0.91) |
| China | 508610 (395853-638618) | 93.28 (75.14-115.5) | 1653117 (1303681-2056459) | 89.76 (72.13-109.67) | -0.21 (-0.31--0.11) |
| Colombia | 15779 (12835-19518) | 107.08 (88.03-131.81) | 57513 (46273-72041) | 102.82 (82.58-129.17) | -0.26 (-0.32--0.21) |
| Comoros | 118 (79-159) | 79.03 (53.09-107.56) | 303 (209-425) | 76.29 (51.65-110.47) | -0.23 (-0.32--0.14) |
| Congo | 920 (695-1253) | 120.05 (93.69-164.53) | 2231 (1708-2802) | 118.63 (92.06-145.96) | -0.14 (-0.22--0.06) |
| Cook Islands | 15 (12-18) | 143.94 (115.36-176.95) | 33 (25-43) | 133.97 (101.01-170.52) | -0.19 (-0.24--0.15) |
| Costa Rica | 1827 (1467-2302) | 112.76 (90.97-140.74) | 6111 (4936-7505) | 109.7 (88.42-135.24) | -0.19 (-0.27--0.11) |
| Croatia | 3763 (3147-4496) | 69.49 (58.62-82.02) | 7519 (6521-8661) | 76.37 (65.94-88.41) | 0.63 (0.34-0.91) |
| Cuba | 11364 (9070-13947) | 118.49 (95.6-143.76) | 23660 (19350-29217) | 113.08 (92.16-140.13) | -0.13 (-0.19--0.08) |
| Cyprus | 1241 (932-1550) | 217.88 (160.27-274.78) | 2333 (1967-2795) | 129.79 (108.76-154.1) | -1.71 (-1.91--1.5) |
| Czechia | 14372 (11698-17635) | 105.91 (86.52-128.9) | 31633 (25775-37952) | 135.11 (109.98-161.89) | 0.99 (0.76-1.22) |
| Côte d'Ivoire | 1990 (1499-2529) | 82.32 (63.19-102.24) | 6579 (5111-8188) | 89.33 (71.39-107.83) | 0.2 (0.14-0.25) |
| Democratic People's Republic of Korea | 12101 (9159-15414) | 103.92 (79.23-132.66) | 29340 (22444-38029) | 103.98 (80.31-138.02) | 0.17 (0.08-0.27) |
| Democratic Republic of the Congo | 9495 (6434-13068) | 87.89 (60.01-121.48) | 24604 (16933-35653) | 94.61 (66.05-140.02) | 0.22 (0.09-0.36) |
| Denmark | 10814 (8775-13295) | 125.94 (101.96-153.71) | 18911 (15746-22897) | 145.11 (120.42-176.86) | 0.31 (0-0.61) |
| Djibouti | 76 (57-100) | 81.99 (62.73-107.18) | 368 (267-489) | 83.68 (61.71-110.9) | 0 (-0.06-0.05) |
| Dominica | 84 (68-102) | 147.85 (120.39-180) | 115 (95-138) | 150.75 (124.68-179.75) | 0.03 (-0.02-0.08) |
| Dominican Republic | 3597 (2866-4515) | 122.8 (100.09-151.2) | 11054 (8859-13676) | 114.8 (92.06-141.9) | -0.01 (-0.16-0.14) |
| Ecuador | 4975 (4021-5988) | 109.78 (90.42-130.68) | 14843 (11786-18496) | 96.93 (77.68-119.73) | -0.38 (-0.44--0.31) |
| Egypt | 12709 (10081-15899) | 76.43 (61.03-96.35) | 30353 (24425-37293) | 75.86 (62.38-91.21) | 0.09 (0.02-0.16) |
| El Salvador | 3201 (2604-3900) | 113.81 (92.47-138.42) | 8071 (6523-9719) | 119.8 (96.6-144.65) | 0.14 (0.08-0.21) |
| Equatorial Guinea | 137 (97-196) | 95.27 (68.76-137.32) | 407 (299-528) | 111.1 (83.53-142.52) | 0.52 (0.45-0.58) |
| Eritrea | 599 (414-827) | 77.05 (53.62-107.15) | 1612 (1063-2379) | 84.53 (54.92-128.65) | 0.23 (0.19-0.26) |
| Estonia | 1863 (1545-2308) | 94.23 (78.75-115.45) | 3559 (3013-4202) | 114.15 (94.72-136.36) | 0.55 (0.49-0.62) |
| Eswatini | 180 (139-236) | 84.13 (64.91-109.9) | 372 (283-474) | 88.58 (67.65-110.66) | 0.47 (0.23-0.71) |
| Ethiopia | 10134 (6772-13928) | 69.44 (45.56-97.24) | 25179 (17422-35300) | 70.26 (48.29-98.85) | -0.03 (-0.11-0.05) |
| Fiji | 344 (282-419) | 124.74 (103.61-151.09) | 843 (664-1017) | 144.64 (115.55-171.88) | 0.4 (0.31-0.5) |
| Finland | 13810 (11123-16890) | 190.5 (153.58-231.88) | 18394 (14929-21967) | 127.35 (103.37-152.38) | -1.47 (-1.6--1.33) |
| France | 113184 (92060-139793) | 128.27 (104.29-157.92) | 182910 (150493-221951) | 107.36 (86.51-132.29) | -0.6 (-0.65--0.55) |
| Gabon | 575 (454-736) | 122.35 (97.53-157.84) | 993 (774-1212) | 129.32 (101.95-154.99) | 0.07 (-0.02-0.15) |
| Gambia | 189 (142-244) | 77.86 (58.66-99.21) | 680 (527-843) | 93.4 (73.11-114.03) | 0.56 (0.52-0.6) |
| Georgia | 5209 (3807-6550) | 89.4 (64.79-113.19) | 7760 (6545-9213) | 122.39 (102.9-145.33) | 1 (0.55-1.45) |
| Germany | 201327 (163031-246392) | 152.56 (123.99-185.96) | 397403 (335970-463533) | 176.87 (148.23-208.48) | 0.72 (0.56-0.89) |
| Ghana | 3553 (2795-4415) | 84.22 (68.07-101.15) | 9818 (7715-12324) | 84.84 (67.39-103.92) | -0.22 (-0.32--0.12) |
| Greece | 16059 (13186-19814) | 109.08 (90.27-133.03) | 30788 (25091-37704) | 105.1 (84.64-130.31) | -0.32 (-0.46--0.19) |
| Greenland | 56 (46-68) | 224.14 (184.04-269.42) | 104 (81-130) | 181.89 (144.48-226.12) | -0.47 (-0.56--0.37) |
| Grenada | 98 (82-119) | 121.66 (100.98-147.69) | 135 (113-159) | 141.52 (120.78-164.76) | 0.4 (0.2-0.61) |
| Guam | 74 (61-89) | 141.86 (120.45-168.14) | 195 (152-244) | 90.63 (70.16-113.59) | -0.95 (-1.22--0.68) |
| Guatemala | 2706 (2179-3315) | 111.72 (93.41-136.28) | 9969 (8055-12254) | 102.34 (83.66-124.24) | -0.28 (-0.37--0.2) |
| Guinea | 1908 (1367-2508) | 72.06 (51.92-95.94) | 3582 (2749-4497) | 82.81 (63.8-103.74) | 0.46 (0.42-0.5) |
| Guinea-Bissau | 229 (171-306) | 85.55 (65.06-112.72) | 417 (314-533) | 94.52 (72.48-119.86) | 0.33 (0.31-0.36) |
| Guyana | 371 (302-451) | 116.75 (96.07-140.9) | 681 (543-834) | 127.56 (103.37-152.5) | 0.32 (0.17-0.48) |
| Haiti | 3107 (2285-4348) | 130.3 (96.61-182.94) | 6840 (4956-9180) | 127.46 (93.23-171.69) | -0.02 (-0.05-0.01) |
| Honduras | 1806 (1390-2322) | 107.39 (82.98-138.54) | 7556 (5799-9174) | 148.88 (115.58-180.09) | 1.12 (0.96-1.28) |
| Hungary | 15494 (12523-19139) | 109.79 (90.18-134.09) | 19640 (15966-23931) | 92.84 (75.06-114.33) | -0.44 (-0.5--0.37) |
| Iceland | 388 (324-467) | 128.16 (106.36-154.7) | 945 (793-1109) | 144.46 (120.93-170.09) | 0.53 (0.43-0.63) |
| India | 239570 (175322-323221) | 69.36 (51.38-93.59) | 855145 (645560-1094554) | 86.05 (65.71-108.52) | 0.82 (0.7-0.94) |
| Indonesia | 75340 (58204-96155) | 101.59 (77.89-129.42) | 223354 (178284-274232) | 133.13 (106.77-160.61) | 0.88 (0.82-0.94) |
| Iran (Islamic Republic of) | 12116 (9430-15214) | 70.54 (55.4-88.33) | 47525 (37528-58271) | 72.39 (57.36-88.32) | 0.02 (-0.03-0.07) |
| Iraq | 5572 (4348-6978) | 76.45 (59.52-96.57) | 16445 (13020-19938) | 93.63 (74.24-114) | 0.36 (0.24-0.47) |
| Ireland | 5569 (4632-6722) | 138.95 (116.12-166.07) | 9551 (7848-11621) | 113.62 (93.02-138.37) | -0.75 (-0.89--0.6) |
| Israel | 6749 (5501-8272) | 145.13 (119.45-175.94) | 19691 (15654-24214) | 148.81 (117.35-184.36) | 0.36 (0.17-0.55) |
| Italy | 106933 (81969-136680) | 120.11 (93.1-152.35) | 206967 (163080-257741) | 116.35 (91.14-146.4) | -0.08 (-0.19-0.03) |
| Jamaica | 2149 (1777-2606) | 116.99 (96.82-142.47) | 4111 (3349-4961) | 123.13 (99.37-149.66) | 0.18 (0.07-0.29) |
| Japan | 132020 (106096-163379) | 81.38 (66.07-100.34) | 270641 (220302-330702) | 61.78 (50.37-76.42) | -1.25 (-1.53--0.97) |
| Jordan | 658 (526-813) | 68.66 (55.41-84.59) | 3377 (2602-4223) | 63.06 (49.36-78.25) | -0.38 (-0.54--0.23) |
| Kazakhstan | 8359 (6442-11000) | 74.49 (57.9-97.3) | 12990 (10082-16723) | 85.35 (68.14-108.69) | 0.22 (0.11-0.33) |
| Kenya | 4433 (3231-5797) | 67.09 (48.23-86.25) | 13715 (9944-18038) | 80.37 (57.75-106.07) | 0.67 (0.61-0.73) |
| Kiribati | 27 (22-34) | 93.66 (74.45-116.98) | 56 (44-70) | 102.38 (80.22-126) | 0.24 (0.19-0.29) |
| Kuwait | 272 (217-339) | 62.26 (49.55-76.91) | 1525 (1212-1889) | 66.57 (52.84-82.17) | 0.4 (0.08-0.72) |
| Kyrgyzstan | 1740 (1344-2258) | 64.88 (50.53-83.45) | 2816 (2187-3622) | 69.06 (54.42-88.3) | 0.22 (0.13-0.31) |
| Lao People's Democratic Republic | 1536 (1129-2018) | 102.38 (75.79-136.34) | 3842 (2968-4839) | 111.23 (87.57-138.44) | 0.23 (0.21-0.25) |
| Latvia | 3115 (2573-3792) | 88.84 (73.85-107.54) | 4759 (4088-5510) | 107.11 (90.35-125.03) | 0.84 (0.72-0.96) |
| Lebanon | 1614 (1080-2271) | 92.94 (60.68-132.36) | 4546 (3720-5509) | 68.9 (56.31-84.18) | -1.13 (-1.25--1.02) |
| Lesotho | 451 (340-597) | 63.7 (47.34-85.86) | 699 (534-890) | 83.87 (64.5-105.66) | 1.43 (1.19-1.67) |
| Liberia | 690 (498-882) | 80.39 (58.15-101.4) | 1248 (940-1587) | 85.7 (65.43-107.19) | 0.22 (0.19-0.26) |
| Libya | 978 (741-1255) | 60.2 (45.09-77.15) | 2950 (2127-3911) | 70.05 (50.35-92.29) | 0.8 (0.67-0.92) |
| Lithuania | 4073 (3334-5057) | 91.65 (75.55-113.69) | 6940 (5789-8373) | 107.61 (88.1-131.18) | 0.55 (0.48-0.62) |
| Luxembourg | 775 (667-905) | 143.61 (124.34-166.52) | 1569 (1347-1837) | 133.18 (114.42-156.51) | -0.02 (-0.1-0.07) |
| Madagascar | 4041 (2847-5160) | 101.25 (70.8-129.17) | 8193 (5940-10725) | 104.41 (74.57-139) | 0.1 (0.01-0.18) |
| Malawi | 1878 (1295-2561) | 64.69 (43.61-87.19) | 4339 (3177-5754) | 75.66 (55.07-100.82) | 0.45 (0.41-0.5) |
| Malaysia | 7513 (5778-9559) | 92.23 (71.37-117.01) | 26502 (20978-32395) | 112.56 (91.2-135.64) | 0.75 (0.58-0.92) |
| Maldives | 58 (42-77) | 97.45 (71.84-126.11) | 261 (204-337) | 93.33 (73.33-118.42) | -0.31 (-0.39--0.24) |
| Mali | 1741 (1280-2332) | 63.27 (46.3-84.06) | 4155 (3145-5466) | 66.21 (50.52-84.94) | 0.22 (0.14-0.29) |
| Malta | 487 (399-589) | 121.54 (100.54-145.77) | 1180 (1005-1388) | 107.08 (90.69-126.34) | -0.16 (-0.3--0.03) |
| Marshall Islands | 19 (15-23) | 145.78 (115.76-183.43) | 41 (30-53) | 156.76 (122.5-196.88) | 0.18 (0.11-0.24) |
| Mauritania | 682 (546-834) | 89.92 (72.58-109.98) | 1595 (1232-1996) | 95.91 (73.63-118.22) | 0.07 (-0.01-0.14) |
| Mauritius | 654 (537-792) | 116.21 (97.68-138.52) | 1849 (1526-2231) | 109.11 (90.73-131.02) | -0.37 (-0.49--0.24) |
| Mexico | 42540 (34462-52381) | 124.51 (103.13-149.87) | 139470 (110869-170421) | 119.77 (96.17-145.26) | -0.08 (-0.11--0.04) |
| Micronesia (Federated States of) | 65 (50-82) | 166.28 (124.82-215.25) | 95 (74-118) | 173.09 (134.52-216.49) | 0.11 (0.09-0.13) |
| Monaco | 93 (73-117) | 117.26 (90.98-147.88) | 125 (97-156) | 109.17 (84.56-138.67) | -0.24 (-0.33--0.15) |
| Mongolia | 639 (483-839) | 69.37 (53.04-90.7) | 1288 (954-1703) | 69.41 (52.42-89.83) | -0.18 (-0.24--0.11) |
| Montenegro | 1184 (1023-1423) | 204.4 (176.69-246.84) | 2331 (1944-2768) | 266.09 (221.18-320.05) | 1.04 (0.81-1.28) |
| Morocco | 7964 (5788-10135) | 67.34 (48.44-86.56) | 23355 (18287-29438) | 83.58 (65.59-104.3) | 0.74 (0.67-0.82) |
| Mozambique | 3346 (2241-4552) | 75 (50.57-101.97) | 7582 (5257-10573) | 92.79 (65.44-130.27) | 0.92 (0.84-1) |
| Myanmar | 17968 (13420-23816) | 103.21 (77.79-135.89) | 43946 (34393-56178) | 111.54 (88.01-140.63) | 0.12 (0.06-0.17) |
| Namibia | 362 (278-466) | 75.68 (58.72-97.54) | 897 (677-1147) | 84.98 (63.14-107.72) | 0.33 (0.21-0.44) |
| Nauru | 6 (5-8) | 179.67 (145.62-221.17) | 9 (7-11) | 204.45 (146.73-299.14) | 0.42 (0.37-0.47) |
| Nepal | 4603 (3202-6368) | 66.88 (45.87-93.49) | 16079 (11993-21120) | 87.28 (65.75-116.28) | 0.94 (0.82-1.07) |
| Netherlands | 30426 (26007-35157) | 148.71 (126.96-172.08) | 50673 (42989-59870) | 130.44 (110.01-154.87) | -0.69 (-0.81--0.58) |
| New Zealand | 6138 (5194-7226) | 161.54 (137.35-188.98) | 14013 (11814-16401) | 154.18 (130.39-181.27) | 0 (-0.12-0.12) |
| Nicaragua | 1252 (974-1576) | 95.1 (74.75-119.04) | 4233 (3296-5278) | 97.61 (76.83-120.84) | 0.22 (0.11-0.34) |
| Niger | 1154 (795-1596) | 62.61 (41.76-88.15) | 3721 (2518-5115) | 66.3 (43.73-90.92) | 0.19 (0.16-0.23) |
| Nigeria | 29033 (22706-36923) | 90.67 (71.86-115.03) | 56183 (42281-70658) | 87.08 (65.99-108.4) | -0.3 (-0.38--0.22) |
| Niue | 4 (3-4) | 151.06 (125.32-182.25) | 3 (3-4) | 155.44 (124.6-184.55) | -0.01 (-0.04-0.03) |
| North Macedonia | 1732 (1418-2143) | 106.33 (88.49-129.52) | 3664 (2801-4933) | 136.26 (107.56-174.16) | 0.72 (0.41-1.03) |
| Northern Mariana Islands | 18 (14-22) | 144.3 (118.96-173.08) | 64 (53-78) | 158.63 (132.79-185.55) | 0.29 (0.16-0.41) |
| Norway | 10706 (8932-12818) | 143.49 (119.37-171.91) | 14671 (12145-17639) | 128.56 (105.7-156.78) | -0.44 (-0.59--0.3) |
| Oman | 413 (310-526) | 80 (60.86-102.78) | 1237 (991-1518) | 90.96 (74.65-109.22) | 0.73 (0.51-0.96) |
| Pakistan | 36868 (26780-49762) | 79.54 (57.9-106.28) | 90387 (69208-116432) | 101.51 (79.65-131.68) | 0.7 (0.56-0.84) |
| Palau | 9 (7-11) | 114.57 (91.97-138.85) | 19 (15-25) | 113.24 (92.02-140.01) | 0.04 (0-0.08) |
| Palestine | 583 (451-731) | 87.07 (67.42-108.73) | 1522 (1249-1838) | 89.76 (74.27-107.6) | -0.01 (-0.14-0.11) |
| Panama | 1430 (1151-1761) | 105.41 (85.8-129.16) | 5027 (3957-6123) | 110.42 (86.72-134.83) | 0.18 (0.14-0.22) |
| Papua New Guinea | 1362 (950-1811) | 97.03 (69.37-127.75) | 4124 (2976-5545) | 102.24 (75.12-137.62) | 0.16 (0.13-0.18) |
| Paraguay | 2431 (1947-3092) | 120.41 (97.06-152.77) | 7067 (5557-8778) | 130.91 (103.06-161.94) | 0.4 (0.32-0.48) |
| Peru | 11138 (8738-13510) | 103.1 (81.39-125.11) | 32300 (24716-40452) | 97.33 (74.34-122.44) | -0.29 (-0.44--0.13) |
| Philippines | 23120 (18497-29118) | 111.79 (90.94-136.5) | 72144 (56995-88259) | 110.96 (89.17-135.18) | 0.12 (0.06-0.18) |
| Poland | 57914 (49713-67500) | 141.14 (122.41-163.83) | 99702 (80721-122377) | 130.17 (104.84-160.59) | -0.34 (-0.7-0.02) |
| Portugal | 15111 (11965-19103) | 116.12 (93.14-144.2) | 28100 (23004-34125) | 96.53 (78.19-118.23) | -0.67 (-0.75--0.59) |
| Puerto Rico | 4226 (3445-5170) | 126.54 (104.91-151.88) | 8769 (7003-10860) | 104.23 (82.57-131.44) | -0.73 (-0.79--0.66) |
| Qatar | 62 (41-78) | 116.22 (65.7-150.08) | 408 (298-555) | 78.49 (45.53-103.69) | -1.66 (-2.1--1.22) |
| Republic of Korea | 22988 (17736-30412) | 93.85 (73.56-124.39) | 89265 (71029-110072) | 96.31 (76.81-117.99) | 0.39 (0.24-0.53) |
| Republic of Moldova | 3481 (2822-4378) | 94.5 (78.13-116.9) | 6007 (4883-7306) | 99.18 (80.48-120.79) | 0.08 (-0.01-0.16) |
| Romania | 22242 (17716-28195) | 90.76 (73.88-112.51) | 31380 (26640-36872) | 77.45 (65.23-91.39) | -0.79 (-0.95--0.62) |
| Russian Federation | 156916 (126821-199504) | 97.18 (79.68-121.88) | 271806 (222263-328016) | 111.42 (91.12-134.81) | 0.43 (0.33-0.54) |
| Rwanda | 1848 (1199-2515) | 86.99 (54.74-118.73) | 3524 (2244-5177) | 73.71 (45.42-110.15) | -0.98 (-1.19--0.77) |
| Saint Kitts and Nevis | 55 (46-65) | 158.08 (133.38-183.44) | 77 (63-92) | 145.54 (123.59-168.74) | -0.12 (-0.24-0) |
| Saint Lucia | 125 (106-146) | 175.93 (153.53-200.83) | 316 (255-375) | 137.1 (111.28-162.68) | -1.24 (-1.43--1.04) |
| Saint Vincent and the Grenadines | 97 (84-115) | 150.93 (131.3-175.74) | 182 (152-215) | 141.74 (120.9-165.99) | -0.17 (-0.31--0.03) |
| Samoa | 99 (76-125) | 143.82 (110.5-180.37) | 180 (143-224) | 146.13 (115.9-180.16) | 0.03 (-0.02-0.08) |
| San Marino | 50 (40-62) | 133.98 (106.29-165.98) | 84 (64-111) | 92.22 (69.55-122.85) | -0.86 (-1.03--0.69) |
| Sao Tome and Principe | 38 (30-52) | 71.04 (54.61-98.35) | 74 (59-90) | 91.18 (73.63-109.63) | 0.94 (0.9-0.99) |
| Saudi Arabia | 2831 (2245-3620) | 64.02 (50.22-81.43) | 9049 (7157-11409) | 72.24 (58.16-88.58) | 0.35 (0.29-0.42) |
| Senegal | 1915 (1430-2460) | 78.78 (58.9-101.14) | 5350 (4182-6681) | 92.09 (72.76-112.97) | 0.41 (0.37-0.45) |
| Serbia | 10432 (8568-12759) | 120.07 (99.86-144.1) | 16205 (13504-19228) | 92.99 (77.52-110.36) | -1.12 (-1.35--0.89) |
| Seychelles | 62 (51-76) | 112.57 (91.59-137.49) | 113 (90-136) | 114.22 (91.68-137.86) | 0.16 (0.05-0.26) |
| Sierra Leone | 1267 (923-1667) | 76.13 (54.99-99.73) | 2275 (1713-2918) | 80.38 (61.47-101.1) | 0.18 (0.16-0.2) |
| Singapore | 1383 (1082-1754) | 69.05 (54.34-86.24) | 4278 (3235-5584) | 50.52 (38.39-65.59) | -1.26 (-1.4--1.12) |
| Slovakia | 8402 (6989-10006) | 144.92 (121.43-171.5) | 14612 (11797-17705) | 150.56 (121.36-182.31) | 0.18 (0.08-0.28) |
| Slovenia | 2338 (1905-2882) | 95.87 (78.17-117.88) | 4654 (3899-5468) | 92.57 (76.97-109.58) | 0.02 (-0.2-0.25) |
| Solomon Islands | 102 (69-139) | 103.32 (67.79-140.33) | 306 (217-408) | 115.15 (78.89-160.17) | 0.34 (0.31-0.36) |
| Somalia | 1182 (742-1682) | 70.06 (43.56-99.59) | 2767 (1735-4169) | 63.59 (39.51-97.45) | -0.27 (-0.32--0.22) |
| South Africa | 13583 (10577-17397) | 76.09 (59.69-97.65) | 34786 (28411-42506) | 91.41 (75.11-110.29) | 0.58 (0.39-0.77) |
| South Sudan | 1617 (1007-2226) | 77.27 (48.54-105.52) | 2148 (1433-2959) | 73.44 (48.25-101.88) | -0.21 (-0.31--0.12) |
| Spain | 71587 (57640-88764) | 132 (107.21-161.79) | 140604 (117419-165622) | 119.53 (98.17-143.01) | -0.26 (-0.35--0.18) |
| Sri Lanka | 7446 (5696-9311) | 90.36 (70.8-111.36) | 22556 (16762-28901) | 95.58 (71.97-120.57) | 0.48 (0.35-0.61) |
| Sudan | 4646 (3340-6250) | 64.76 (46.08-88.58) | 10967 (8324-13689) | 72.54 (56.08-90.42) | 0.34 (0.28-0.4) |
| Suriname | 291 (241-350) | 127.17 (105.45-152.1) | 706 (544-876) | 119.94 (93.43-148.75) | -0.06 (-0.15-0.03) |
| Sweden | 22758 (17956-28457) | 137.78 (108.56-173.28) | 57180 (45730-70747) | 222 (175.69-277.17) | 1.61 (1.46-1.77) |
| Switzerland | 7852 (6377-9611) | 70.15 (56.77-85.88) | 14918 (12437-17545) | 69.34 (57.59-82.94) | 0.37 (0.17-0.57) |
| Syrian Arab Republic | 3247 (2558-4087) | 79.86 (62.62-101.42) | 8108 (6087-10069) | 85.65 (63.76-105.23) | -0.02 (-0.14-0.09) |
| Taiwan (Province of China) | 13126 (10402-16164) | 112.17 (92.95-134.45) | 41012 (33444-50586) | 92.14 (74.48-113.99) | -0.83 (-1.02--0.63) |
| Tajikistan | 1358 (1009-1881) | 55.03 (41.21-75.82) | 2547 (1826-3444) | 54.26 (39.71-72.32) | -0.11 (-0.22-0) |
| Thailand | 30152 (23731-38013) | 112.77 (89.31-141.37) | 110567 (87502-136071) | 101.05 (80.12-124.39) | -0.68 (-0.8--0.56) |
| Timor-Leste | 179 (131-233) | 90.69 (66.63-119.68) | 692 (531-906) | 102.36 (80.67-131.25) | 0.46 (0.39-0.53) |
| Togo | 674 (522-859) | 78.55 (61.25-99.57) | 2198 (1650-2761) | 89.23 (69.02-110.26) | 0.38 (0.35-0.4) |
| Tokelau | 2 (1-2) | 152.92 (124.85-190.22) | 2 (2-3) | 149.97 (112.24-200.51) | -0.06 (-0.09--0.03) |
| Tonga | 51 (39-67) | 109.91 (83.21-144.28) | 91 (71-117) | 119.97 (93.23-153.34) | 0.3 (0.23-0.37) |
| Trinidad and Tobago | 1001 (827-1210) | 147.3 (125.02-173.65) | 2386 (1898-2915) | 128.6 (102.59-156.53) | -0.43 (-0.49--0.37) |
| Tunisia | 2561 (1967-3177) | 70.85 (55.59-87.76) | 9283 (6895-12247) | 81.82 (60.46-107.55) | 0.36 (0.32-0.41) |
| Turkmenistan | 1138 (887-1460) | 70.46 (55.65-90.88) | 2975 (2299-3738) | 86.5 (67.67-106.69) | 0.53 (0.44-0.61) |
| Tuvalu | 8 (6-10) | 149.09 (117.45-187.98) | 13 (10-16) | 150.94 (121.58-183.48) | 0.01 (-0.02-0.03) |
| Türkiye | 19964 (15940-24779) | 72.06 (57.9-89.75) | 55942 (46418-65880) | 67.92 (56.42-80.43) | -0.11 (-0.46-0.25) |
| Uganda | 3639 (2371-5067) | 70.74 (45.45-99.84) | 8476 (5794-12027) | 73.52 (49.36-105.55) | -0.01 (-0.07-0.04) |
| Ukraine | 61515 (49362-75818) | 92.65 (75.62-113.39) | 78770 (61400-97874) | 98.56 (76.69-121.96) | 0.04 (-0.07-0.14) |
| United Arab Emirates | 232 (171-301) | 79.54 (60.3-104.87) | 1763 (1273-2342) | 85.82 (66.1-105.16) | 1.96 (1.34-2.58) |
| United Kingdom | 110739 (93505-131324) | 117 (99.11-138.75) | 176779 (150586-207501) | 119.73 (101.41-141.22) | 0.08 (-0.03-0.19) |
| United Republic of Tanzania | 6566 (4530-8842) | 79.46 (53.67-107.3) | 16779 (11752-23254) | 82.33 (57.78-113.53) | -0.06 (-0.23-0.12) |
| United States of America | 393871 (306983-495874) | 118.44 (92.41-149.14) | 916645 (768611-1080294) | 146.72 (122.77-172.78) | 0.68 (0.64-0.72) |
| United States Virgin Islands | 96 (77-118) | 149.87 (122.66-179.17) | 201 (151-252) | 113.8 (87.47-142) | -0.79 (-0.92--0.67) |
| Uruguay | 2984 (2412-3683) | 77.36 (62.87-95.1) | 5256 (4397-6336) | 82.04 (68.63-99.71) | 0.24 (0.18-0.3) |
| Uzbekistan | 5343 (3749-7549) | 50.19 (35.2-71.03) | 12966 (9657-17427) | 58.96 (44.73-77.9) | 0.59 (0.52-0.65) |
| Vanuatu | 58 (41-76) | 120.37 (87.79-158.55) | 175 (135-222) | 127.94 (98.97-160.53) | 0.14 (0.11-0.18) |
| Venezuela (Bolivarian Republic of) | 9691 (7702-11944) | 116.92 (94.53-143.09) | 31981 (25013-39311) | 114.29 (89.6-140.04) | -0.16 (-0.23--0.08) |
| Viet Nam | 33051 (25239-41932) | 95.92 (73.96-120.29) | 97149 (74777-122074) | 118.87 (91.48-147.64) | 0.74 (0.69-0.78) |
| Yemen | 2246 (1544-3172) | 65.51 (44.69-94.69) | 7685 (5625-9928) | 75.17 (55.62-97.35) | 0.41 (0.38-0.44) |
| Zambia | 1828 (1391-2272) | 86.65 (65.76-107.38) | 5743 (3778-9002) | 112.52 (76.94-168.22) | 0.93 (0.72-1.14) |
| Zimbabwe | 2159 (1653-2691) | 72.21 (55.8-88.6) | 4171 (3086-5215) | 82.48 (61.95-102) | 0.67 (0.46-0.87) |

ASDAR =Age-standardized DALYs rate, CI=Confidence interval, EAPC =Estimated annual percentage change, UI =Uncertainty interval
