# Supplementary material for: Global burden of atrial fibrillation/atrial flutter and its attributable risk factors from 1990 to 2021
Source: Europace. 2024 Jul 10;26(7):euae195. doi: 10.1093/europace/euae195 (PMC11287210; doi:10.1093/europace/euae195)
Supplement: euae195_Supplementary_Data [file euae195_supplementary_data.zip › Table S5.docx]

Table S5 The top5 risk factors of AF/AFL in 1990 and 2021, by SDI quintile

| **metric** | **location** | **risk factors** | **percent** | **year** |
| --- | --- | --- | --- | --- |
| Deaths number | Global | Tobacco | 0.0829685 | 1990 |
| Deaths number | Global | High systolic blood pressure | 0.6411136 | 1990 |
| Deaths number | Global | High body-mass index | 0.0995371 | 1990 |
| Deaths number | Global | Dietary risks | 0.0582761 | 1990 |
| Deaths number | Global | Other environmental risks | 0.0431578 | 1990 |
| Deaths number | Global | Alcohol use | 0.0749469 | 1990 |
| Deaths number | High SDI | Tobacco | 0.0728539 | 1990 |
| Deaths number | High SDI | High systolic blood pressure | 0.6402132 | 1990 |
| Deaths number | High SDI | High body-mass index | 0.1219382 | 1990 |
| Deaths number | High SDI | Dietary risks | 0.0405638 | 1990 |
| Deaths number | High SDI | Other environmental risks | 0.0269384 | 1990 |
| Deaths number | High SDI | Alcohol use | 0.0974926 | 1990 |
| Deaths number | High-middle SDI | Tobacco | 0.0737049 | 1990 |
| Deaths number | High-middle SDI | High systolic blood pressure | 0.6356146 | 1990 |
| Deaths number | High-middle SDI | High body-mass index | 0.1174022 | 1990 |
| Deaths number | High-middle SDI | Dietary risks | 0.0631542 | 1990 |
| Deaths number | High-middle SDI | Other environmental risks | 0.036135 | 1990 |
| Deaths number | High-middle SDI | Alcohol use | 0.0739891 | 1990 |
| Deaths number | Low SDI | Tobacco | 0.0852976 | 1990 |
| Deaths number | Low SDI | High systolic blood pressure | 0.6878616 | 1990 |
| Deaths number | Low SDI | High body-mass index | 0.0214255 | 1990 |
| Deaths number | Low SDI | Dietary risks | 0.0597907 | 1990 |
| Deaths number | Low SDI | Other environmental risks | 0.1061809 | 1990 |
| Deaths number | Low SDI | Alcohol use | 0.0394438 | 1990 |
| Deaths number | Low-middle SDI | Tobacco | 0.1111581 | 1990 |
| Deaths number | Low-middle SDI | High systolic blood pressure | 0.6645879 | 1990 |
| Deaths number | Low-middle SDI | High body-mass index | 0.0440614 | 1990 |
| Deaths number | Low-middle SDI | Dietary risks | 0.0615304 | 1990 |
| Deaths number | Low-middle SDI | Other environmental risks | 0.094024 | 1990 |
| Deaths number | Low-middle SDI | Alcohol use | 0.0246383 | 1990 |
| Deaths number | Middle SDI | Tobacco | 0.1125619 | 1990 |
| Deaths number | Middle SDI | High systolic blood pressure | 0.6344789 | 1990 |
| Deaths number | Middle SDI | High body-mass index | 0.045533 | 1990 |
| Deaths number | Middle SDI | Dietary risks | 0.0995604 | 1990 |
| Deaths number | Middle SDI | Other environmental risks | 0.0672151 | 1990 |
| Deaths number | Middle SDI | Alcohol use | 0.0406508 | 1990 |
| Deaths number | Global | Tobacco | 0.0584978 | 2021 |
| Deaths number | Global | High systolic blood pressure | 0.6042369 | 2021 |
| Deaths number | Global | High body-mass index | 0.1591273 | 2021 |
| Deaths number | Global | Dietary risks | 0.0556745 | 2021 |
| Deaths number | Global | Other environmental risks | 0.052892 | 2021 |
| Deaths number | Global | Alcohol use | 0.0695715 | 2021 |
| Deaths number | High SDI | Tobacco | 0.0486772 | 2021 |
| Deaths number | High SDI | High systolic blood pressure | 0.5750167 | 2021 |
| Deaths number | High SDI | High body-mass index | 0.1982399 | 2021 |
| Deaths number | High SDI | Dietary risks | 0.0437671 | 2021 |
| Deaths number | High SDI | Other environmental risks | 0.0306782 | 2021 |
| Deaths number | High SDI | Alcohol use | 0.1036209 | 2021 |
| Deaths number | High-middle SDI | Tobacco | 0.0573876 | 2021 |
| Deaths number | High-middle SDI | High systolic blood pressure | 0.5964423 | 2021 |
| Deaths number | High-middle SDI | High body-mass index | 0.1747795 | 2021 |
| Deaths number | High-middle SDI | Dietary risks | 0.0604358 | 2021 |
| Deaths number | High-middle SDI | Other environmental risks | 0.0473766 | 2021 |
| Deaths number | High-middle SDI | Alcohol use | 0.0635782 | 2021 |
| Deaths number | Low SDI | Tobacco | 0.0629138 | 2021 |
| Deaths number | Low SDI | High systolic blood pressure | 0.6731066 | 2021 |
| Deaths number | Low SDI | High body-mass index | 0.0583744 | 2021 |
| Deaths number | Low SDI | Dietary risks | 0.0489518 | 2021 |
| Deaths number | Low SDI | Other environmental risks | 0.1151293 | 2021 |
| Deaths number | Low SDI | Alcohol use | 0.0415241 | 2021 |
| Deaths number | Low-middle SDI | Tobacco | 0.0770292 | 2021 |
| Deaths number | Low-middle SDI | High systolic blood pressure | 0.6482517 | 2021 |
| Deaths number | Low-middle SDI | High body-mass index | 0.0962546 | 2021 |
| Deaths number | Low-middle SDI | Dietary risks | 0.0526302 | 2021 |
| Deaths number | Low-middle SDI | Other environmental risks | 0.0972271 | 2021 |
| Deaths number | Low-middle SDI | Alcohol use | 0.0286072 | 2021 |
| Deaths number | Middle SDI | Tobacco | 0.0665688 | 2021 |
| Deaths number | Middle SDI | High systolic blood pressure | 0.6316722 | 2021 |
| Deaths number | Middle SDI | High body-mass index | 0.1199445 | 2021 |
| Deaths number | Middle SDI | Dietary risks | 0.0720627 | 2021 |
| Deaths number | Middle SDI | Other environmental risks | 0.0672415 | 2021 |
| Deaths number | Middle SDI | Alcohol use | 0.0425103 | 2021 |
| Deaths rate | Global | Tobacco | 0.0737528 | 1990 |
| Deaths rate | Global | High systolic blood pressure | 0.6538475 | 1990 |
| Deaths rate | Global | High body-mass index | 0.1013336 | 1990 |
| Deaths rate | Global | Dietary risks | 0.0556342 | 1990 |
| Deaths rate | Global | Other environmental risks | 0.0427224 | 1990 |
| Deaths rate | Global | Alcohol use | 0.0727095 | 1990 |
| Deaths rate | High SDI | Tobacco | 0.0713062 | 1990 |
| Deaths rate | High SDI | High systolic blood pressure | 0.6420024 | 1990 |
| Deaths rate | High SDI | High body-mass index | 0.1228316 | 1990 |
| Deaths rate | High SDI | Dietary risks | 0.0401842 | 1990 |
| Deaths rate | High SDI | Other environmental risks | 0.0272525 | 1990 |
| Deaths rate | High SDI | Alcohol use | 0.0964231 | 1990 |
| Deaths rate | High-middle SDI | Tobacco | 0.0644087 | 1990 |
| Deaths rate | High-middle SDI | High systolic blood pressure | 0.6513884 | 1990 |
| Deaths rate | High-middle SDI | High body-mass index | 0.1174437 | 1990 |
| Deaths rate | High-middle SDI | Dietary risks | 0.0597276 | 1990 |
| Deaths rate | High-middle SDI | Other environmental risks | 0.0366954 | 1990 |
| Deaths rate | High-middle SDI | Alcohol use | 0.0703362 | 1990 |
| Deaths rate | Low SDI | Tobacco | 0.0717843 | 1990 |
| Deaths rate | Low SDI | High systolic blood pressure | 0.707389 | 1990 |
| Deaths rate | Low SDI | High body-mass index | 0.0184239 | 1990 |
| Deaths rate | Low SDI | Dietary risks | 0.05882 | 1990 |
| Deaths rate | Low SDI | Other environmental risks | 0.1085615 | 1990 |
| Deaths rate | Low SDI | Alcohol use | 0.0350214 | 1990 |
| Deaths rate | Low-middle SDI | Tobacco | 0.0973042 | 1990 |
| Deaths rate | Low-middle SDI | High systolic blood pressure | 0.679847 | 1990 |
| Deaths rate | Low-middle SDI | High body-mass index | 0.0438378 | 1990 |
| Deaths rate | Low-middle SDI | Dietary risks | 0.0611783 | 1990 |
| Deaths rate | Low-middle SDI | Other environmental risks | 0.0948794 | 1990 |
| Deaths rate | Low-middle SDI | Alcohol use | 0.0229533 | 1990 |
| Deaths rate | Middle SDI | Tobacco | 0.0967187 | 1990 |
| Deaths rate | Middle SDI | High systolic blood pressure | 0.6610373 | 1990 |
| Deaths rate | Middle SDI | High body-mass index | 0.0446593 | 1990 |
| Deaths rate | Middle SDI | Dietary risks | 0.093889 | 1990 |
| Deaths rate | Middle SDI | Other environmental risks | 0.0678235 | 1990 |
| Deaths rate | Middle SDI | Alcohol use | 0.0358722 | 1990 |
| Deaths rate | Global | Tobacco | 0.0566422 | 2021 |
| Deaths rate | Global | High systolic blood pressure | 0.6068194 | 2021 |
| Deaths rate | Global | High body-mass index | 0.159201 | 2021 |
| Deaths rate | Global | Dietary risks | 0.0551883 | 2021 |
| Deaths rate | Global | Other environmental risks | 0.0530293 | 2021 |
| Deaths rate | Global | Alcohol use | 0.0691199 | 2021 |
| Deaths rate | High SDI | Tobacco | 0.0531969 | 2021 |
| Deaths rate | High SDI | High systolic blood pressure | 0.5670027 | 2021 |
| Deaths rate | High SDI | High body-mass index | 0.1996348 | 2021 |
| Deaths rate | High SDI | Dietary risks | 0.0441845 | 2021 |
| Deaths rate | High SDI | Other environmental risks | 0.0300334 | 2021 |
| Deaths rate | High SDI | Alcohol use | 0.1059477 | 2021 |
| Deaths rate | High-middle SDI | Tobacco | 0.0558829 | 2021 |
| Deaths rate | High-middle SDI | High systolic blood pressure | 0.5981514 | 2021 |
| Deaths rate | High-middle SDI | High body-mass index | 0.1750884 | 2021 |
| Deaths rate | High-middle SDI | Dietary risks | 0.0598192 | 2021 |
| Deaths rate | High-middle SDI | Other environmental risks | 0.0479688 | 2021 |
| Deaths rate | High-middle SDI | Alcohol use | 0.0630893 | 2021 |
| Deaths rate | Low SDI | Tobacco | 0.054768 | 2021 |
| Deaths rate | Low SDI | High systolic blood pressure | 0.6824234 | 2021 |
| Deaths rate | Low SDI | High body-mass index | 0.0538798 | 2021 |
| Deaths rate | Low SDI | Dietary risks | 0.0496037 | 2021 |
| Deaths rate | Low SDI | Other environmental risks | 0.1210323 | 2021 |
| Deaths rate | Low SDI | Alcohol use | 0.0382929 | 2021 |
| Deaths rate | Low-middle SDI | Tobacco | 0.0711268 | 2021 |
| Deaths rate | Low-middle SDI | High systolic blood pressure | 0.6543364 | 2021 |
| Deaths rate | Low-middle SDI | High body-mass index | 0.095248 | 2021 |
| Deaths rate | Low-middle SDI | Dietary risks | 0.0526176 | 2021 |
| Deaths rate | Low-middle SDI | Other environmental risks | 0.1002276 | 2021 |
| Deaths rate | Low-middle SDI | Alcohol use | 0.0264436 | 2021 |
| Deaths rate | Middle SDI | Tobacco | 0.0617488 | 2021 |
| Deaths rate | Middle SDI | High systolic blood pressure | 0.6400949 | 2021 |
| Deaths rate | Middle SDI | High body-mass index | 0.1183309 | 2021 |
| Deaths rate | Middle SDI | Dietary risks | 0.0707126 | 2021 |
| Deaths rate | Middle SDI | Other environmental risks | 0.0688543 | 2021 |
| Deaths rate | Middle SDI | Alcohol use | 0.0402585 | 2021 |
| DALYs number | Global | Tobacco | 0.1219993 | 1990 |
| DALYs number | Global | High systolic blood pressure | 0.5852948 | 1990 |
| DALYs number | Global | High body-mass index | 0.0976293 | 1990 |
| DALYs number | Global | Dietary risks | 0.0636849 | 1990 |
| DALYs number | Global | Other environmental risks | 0.0445434 | 1990 |
| DALYs number | Global | Alcohol use | 0.0868483 | 1990 |
| DALYs number | High SDI | Tobacco | 0.1065571 | 1990 |
| DALYs number | High SDI | High systolic blood pressure | 0.5897003 | 1990 |
| DALYs number | High SDI | High body-mass index | 0.1241438 | 1990 |
| DALYs number | High SDI | Dietary risks | 0.041565 | 1990 |
| DALYs number | High SDI | Other environmental risks | 0.0263252 | 1990 |
| DALYs number | High SDI | Alcohol use | 0.1117087 | 1990 |
| DALYs number | High-middle SDI | Tobacco | 0.1141603 | 1990 |
| DALYs number | High-middle SDI | High systolic blood pressure | 0.5716569 | 1990 |
| DALYs number | High-middle SDI | High body-mass index | 0.1152323 | 1990 |
| DALYs number | High-middle SDI | Dietary risks | 0.0692535 | 1990 |
| DALYs number | High-middle SDI | Other environmental risks | 0.0352476 | 1990 |
| DALYs number | High-middle SDI | Alcohol use | 0.0944493 | 1990 |
| DALYs number | Low SDI | Tobacco | 0.1133927 | 1990 |
| DALYs number | Low SDI | High systolic blood pressure | 0.6551582 | 1990 |
| DALYs number | Low SDI | High body-mass index | 0.0244082 | 1990 |
| DALYs number | Low SDI | Dietary risks | 0.0581074 | 1990 |
| DALYs number | Low SDI | Other environmental risks | 0.1067194 | 1990 |
| DALYs number | Low SDI | Alcohol use | 0.0422142 | 1990 |
| DALYs number | Low-middle SDI | Tobacco | 0.149857 | 1990 |
| DALYs number | Low-middle SDI | High systolic blood pressure | 0.6200406 | 1990 |
| DALYs number | Low-middle SDI | High body-mass index | 0.0421339 | 1990 |
| DALYs number | Low-middle SDI | Dietary risks | 0.0639823 | 1990 |
| DALYs number | Low-middle SDI | Other environmental risks | 0.0940476 | 1990 |
| DALYs number | Low-middle SDI | Alcohol use | 0.0299385 | 1990 |
| DALYs number | Middle SDI | Tobacco | 0.1560862 | 1990 |
| DALYs number | Middle SDI | High systolic blood pressure | 0.5664887 | 1990 |
| DALYs number | Middle SDI | High body-mass index | 0.0504173 | 1990 |
| DALYs number | Middle SDI | Dietary risks | 0.1074497 | 1990 |
| DALYs number | Middle SDI | Other environmental risks | 0.0655253 | 1990 |
| DALYs number | Middle SDI | Alcohol use | 0.0540328 | 1990 |
| DALYs number | Global | Tobacco | 0.0879776 | 2021 |
| DALYs number | Global | High systolic blood pressure | 0.5583572 | 2021 |
| DALYs number | Global | High body-mass index | 0.1608899 | 2021 |
| DALYs number | Global | Dietary risks | 0.062719 | 2021 |
| DALYs number | Global | Other environmental risks | 0.04952 | 2021 |
| DALYs number | Global | Alcohol use | 0.0805363 | 2021 |
| DALYs number | High SDI | Tobacco | 0.074064 | 2021 |
| DALYs number | High SDI | High systolic blood pressure | 0.525289 | 2021 |
| DALYs number | High SDI | High body-mass index | 0.2090193 | 2021 |
| DALYs number | High SDI | Dietary risks | 0.0463122 | 2021 |
| DALYs number | High SDI | Other environmental risks | 0.0277396 | 2021 |
| DALYs number | High SDI | Alcohol use | 0.1175759 | 2021 |
| DALYs number | High-middle SDI | Tobacco | 0.0928344 | 2021 |
| DALYs number | High-middle SDI | High systolic blood pressure | 0.5438263 | 2021 |
| DALYs number | High-middle SDI | High body-mass index | 0.170257 | 2021 |
| DALYs number | High-middle SDI | Dietary risks | 0.0714249 | 2021 |
| DALYs number | High-middle SDI | Other environmental risks | 0.0416661 | 2021 |
| DALYs number | High-middle SDI | Alcohol use | 0.0799912 | 2021 |
| DALYs number | Low SDI | Tobacco | 0.0800241 | 2021 |
| DALYs number | Low SDI | High systolic blood pressure | 0.6479644 | 2021 |
| DALYs number | Low SDI | High body-mass index | 0.0680756 | 2021 |
| DALYs number | Low SDI | Dietary risks | 0.0489898 | 2021 |
| DALYs number | Low SDI | Other environmental risks | 0.1061234 | 2021 |
| DALYs number | Low SDI | Alcohol use | 0.0488227 | 2021 |
| DALYs number | Low-middle SDI | Tobacco | 0.0987329 | 2021 |
| DALYs number | Low-middle SDI | High systolic blood pressure | 0.6176013 | 2021 |
| DALYs number | Low-middle SDI | High body-mass index | 0.1038328 | 2021 |
| DALYs number | Low-middle SDI | Dietary risks | 0.054328 | 2021 |
| DALYs number | Low-middle SDI | Other environmental risks | 0.0890862 | 2021 |
| DALYs number | Low-middle SDI | Alcohol use | 0.0364188 | 2021 |
| DALYs number | Middle SDI | Tobacco | 0.097789 | 2021 |
| DALYs number | Middle SDI | High systolic blood pressure | 0.5768086 | 2021 |
| DALYs number | Middle SDI | High body-mass index | 0.1268022 | 2021 |
| DALYs number | Middle SDI | Dietary risks | 0.0817196 | 2021 |
| DALYs number | Middle SDI | Other environmental risks | 0.0601474 | 2021 |
| DALYs number | Middle SDI | Alcohol use | 0.0567331 | 2021 |
| DALYs rate | Global | Tobacco | 0.1096634 | 1990 |
| DALYs rate | Global | High systolic blood pressure | 0.6029051 | 1990 |
| DALYs rate | Global | High body-mass index | 0.0985855 | 1990 |
| DALYs rate | Global | Dietary risks | 0.0612416 | 1990 |
| DALYs rate | Global | Other environmental risks | 0.0441207 | 1990 |
| DALYs rate | Global | Alcohol use | 0.0834838 | 1990 |
| DALYs rate | High SDI | Tobacco | 0.1083756 | 1990 |
| DALYs rate | High SDI | High systolic blood pressure | 0.5871678 | 1990 |
| DALYs rate | High SDI | High body-mass index | 0.1243849 | 1990 |
| DALYs rate | High SDI | Dietary risks | 0.0415593 | 1990 |
| DALYs rate | High SDI | Other environmental risks | 0.0264377 | 1990 |
| DALYs rate | High SDI | Alcohol use | 0.1120748 | 1990 |
| DALYs rate | High-middle SDI | Tobacco | 0.1027079 | 1990 |
| DALYs rate | High-middle SDI | High systolic blood pressure | 0.5903567 | 1990 |
| DALYs rate | High-middle SDI | High body-mass index | 0.1152566 | 1990 |
| DALYs rate | High-middle SDI | Dietary risks | 0.0663625 | 1990 |
| DALYs rate | High-middle SDI | Other environmental risks | 0.0356411 | 1990 |
| DALYs rate | High-middle SDI | Alcohol use | 0.0896753 | 1990 |
| DALYs rate | Low SDI | Tobacco | 0.0963163 | 1990 |
| DALYs rate | Low SDI | High systolic blood pressure | 0.6784407 | 1990 |
| DALYs rate | Low SDI | High body-mass index | 0.0206953 | 1990 |
| DALYs rate | Low SDI | Dietary risks | 0.0582208 | 1990 |
| DALYs rate | Low SDI | Other environmental risks | 0.1086242 | 1990 |
| DALYs rate | Low SDI | Alcohol use | 0.0377027 | 1990 |
| DALYs rate | Low-middle SDI | Tobacco | 0.1310884 | 1990 |
| DALYs rate | Low-middle SDI | High systolic blood pressure | 0.6434357 | 1990 |
| DALYs rate | Low-middle SDI | High body-mass index | 0.0405672 | 1990 |
| DALYs rate | Low-middle SDI | Dietary risks | 0.0629272 | 1990 |
| DALYs rate | Low-middle SDI | Other environmental risks | 0.0951431 | 1990 |
| DALYs rate | Low-middle SDI | Alcohol use | 0.0268384 | 1990 |
| DALYs rate | Middle SDI | Tobacco | 0.133324 | 1990 |
| DALYs rate | Middle SDI | High systolic blood pressure | 0.6031401 | 1990 |
| DALYs rate | Middle SDI | High body-mass index | 0.0478635 | 1990 |
| DALYs rate | Middle SDI | Dietary risks | 0.1021828 | 1990 |
| DALYs rate | Middle SDI | Other environmental risks | 0.0662942 | 1990 |
| DALYs rate | Middle SDI | Alcohol use | 0.0471954 | 1990 |
| DALYs rate | Global | Tobacco | 0.0852206 | 2021 |
| DALYs rate | Global | High systolic blood pressure | 0.5627309 | 2021 |
| DALYs rate | Global | High body-mass index | 0.1606464 | 2021 |
| DALYs rate | Global | Dietary risks | 0.0619179 | 2021 |
| DALYs rate | Global | Other environmental risks | 0.0498207 | 2021 |
| DALYs rate | Global | Alcohol use | 0.0796635 | 2021 |
| DALYs rate | High SDI | Tobacco | 0.0840069 | 2021 |
| DALYs rate | High SDI | High systolic blood pressure | 0.5092167 | 2021 |
| DALYs rate | High SDI | High body-mass index | 0.2113184 | 2021 |
| DALYs rate | High SDI | Dietary risks | 0.0468015 | 2021 |
| DALYs rate | High SDI | Other environmental risks | 0.0266932 | 2021 |
| DALYs rate | High SDI | Alcohol use | 0.1219633 | 2021 |
| DALYs rate | High-middle SDI | Tobacco | 0.0917986 | 2021 |
| DALYs rate | High-middle SDI | High systolic blood pressure | 0.5458019 | 2021 |
| DALYs rate | High-middle SDI | High body-mass index | 0.1702783 | 2021 |
| DALYs rate | High-middle SDI | Dietary risks | 0.070595 | 2021 |
| DALYs rate | High-middle SDI | Other environmental risks | 0.0420456 | 2021 |
| DALYs rate | High-middle SDI | Alcohol use | 0.0794806 | 2021 |
| DALYs rate | Low SDI | Tobacco | 0.0690565 | 2021 |
| DALYs rate | Low SDI | High systolic blood pressure | 0.6629312 | 2021 |
| DALYs rate | Low SDI | High body-mass index | 0.0610148 | 2021 |
| DALYs rate | Low SDI | Dietary risks | 0.0497714 | 2021 |
| DALYs rate | Low SDI | Other environmental risks | 0.1130988 | 2021 |
| DALYs rate | Low SDI | Alcohol use | 0.0441273 | 2021 |
| DALYs rate | Low-middle SDI | Tobacco | 0.0900299 | 2021 |
| DALYs rate | Low-middle SDI | High systolic blood pressure | 0.6297746 | 2021 |
| DALYs rate | Low-middle SDI | High body-mass index | 0.1003083 | 2021 |
| DALYs rate | Low-middle SDI | Dietary risks | 0.0538196 | 2021 |
| DALYs rate | Low-middle SDI | Other environmental risks | 0.0928027 | 2021 |
| DALYs rate | Low-middle SDI | Alcohol use | 0.0332648 | 2021 |
| DALYs rate | Middle SDI | Tobacco | 0.0898324 | 2021 |
| DALYs rate | Middle SDI | High systolic blood pressure | 0.5910588 | 2021 |
| DALYs rate | Middle SDI | High body-mass index | 0.1244518 | 2021 |
| DALYs rate | Middle SDI | Dietary risks | 0.0792107 | 2021 |
| DALYs rate | Middle SDI | Other environmental risks | 0.0622202 | 2021 |
| DALYs rate | Middle SDI | Alcohol use | 0.0532261 | 2021 |

DALYs=disability-adjusted life-years, SDI=socio-demographic Index
