# Supplementary material for: Global burden of atrial fibrillation/atrial flutter and its attributable risk factors from 1990 to 2021
Source: Europace. 2024 Jul 10;26(7):euae195. doi: 10.1093/europace/euae195 (PMC11287210; doi:10.1093/europace/euae195)
Supplement: euae195_Supplementary_Data [file euae195_supplementary_data.zip › Supplementary Information and Data.docx]

**Supplementary Information and Data**

**Global burden of atrial fibrillation/atrial flutter and its attributable risk factors from 1990 to 2021**

**Supplementary Figures:**

**Figure S1** Age-standardized rate and numbers of incidence, prevalence, deaths, and DALYs change curves for AF/AFL patients by ages from 1990 to 2021 (A: Age-standardized rate, B: Number of cases), AF=atrial fibrillation, AFL=Atrial flutter, DAYLs=disability-adjusted life-years

**Figure S2** Age-standardized rate and numbers of incidence, prevalence, deaths, and DALYs change curves for AF/AFL patients by sex from 1990 to 2021 (A: Age-standardized rate, B: Number of cases), AF=atrial fibrillation, AFL=Atrial flutter, DAYLs=disability-adjusted life-years

**Figure S3** Age-standardized rate and numbers of incidence, prevalence, deaths, and DALYs change curves for AF/AFL patients by SDI from 1990 to 2021 (A: Age-standardized rate, B: Number of cases), AF=atrial fibrillation, AFL=Atrial flutter, DAYLs=disability-adjusted life-years, SDI=socio-demographic index

**Figure S4** The clusters of countries and territories in terms of the temporal trends related EAPC of incidence of AF/AFL, AF=atrial fibrillation, AFL=Atrial flutter, EAPC= estimated annual percentage change

**Figure S5** Age-standardized rate and numbers of incidence, prevalence, deaths, and DALYs of AF/AFL by age, 2021, AF=atrial fibrillation, AFL=Atrial flutter, DAYLs=disability-adjusted life-years

**Figure S6** Age-standardized rate and numbers of incidence, prevalence, deaths, and DALYs of AF/AFL by sex, 2021, AF=atrial fibrillation, AFL=Atrial flutter, DAYLs=disability-adjusted life-years

**Figure S7** Age-standardized rate and numbers of incidence, prevalence, deaths, and DALYs of AF/AFL by SDI, 2021, AF=atrial fibrillation, AFL=Atrial flutter, DAYLs=disability-adjusted life-years, SDI=socio-demographic index

**Figure S8** Age-standardized incidence, prevalence, deaths, and DALYs rates for AF/AFL for 204 countries and territories (1,2) by SDI, 1990–2021 Expected values based on SDI and disease rates in all locations are shown as the black line, AF=atrial fibrillation, AFL=Atrial flutter, DAYLs=disability-adjusted life-years, SDI=socio-demographic index

**Figure S9** Age-standardized rate and numbers of incidence, prevalence, deaths, and DALYs of AF/AFL by all GBD regions, 2021, AF=atrial fibrillation, AFL=Atrial flutter, DAYLs=disability-adjusted life-years

**Figure S10** The correlation between EAPC and AF/AFL ASR in 2021 (A) and HDI in 2021 (B). The circles represent countries that were available on HDI data. The size of circle is increased with the cases of AF/AFL. The ρ indices and p-values presented in (A) and (B) were derived from Pearson correlation analysis. AF=atrial fibrillation, AFL=Atrial flutter, EAPC=estimated annual percentage change, HDI=human development index

**Figure S11** Proportion of age-standardized rate and numbers of cases of deaths and DALYs due to AF/AFL attributable to three main categories risk factors for Global Burden of Disease regions by SDI, both sexes, 2021, AF=atrial fibrillation, AFL=Atrial flutter, DAYLs=disability-adjusted life-years, SDI=socio-demographic index

**Figure S12** Proportion of age-standardized rate and numbers of cases of deaths and DALYs due to AF/AFL attributable to five subcategories risk factors for Global Burden of Disease regions by SDI, both sexes, 2021, AF=atrial fibrillation, AFL=Atrial flutter, DAYLs=disability-adjusted life-years, SDI=socio-demographic index


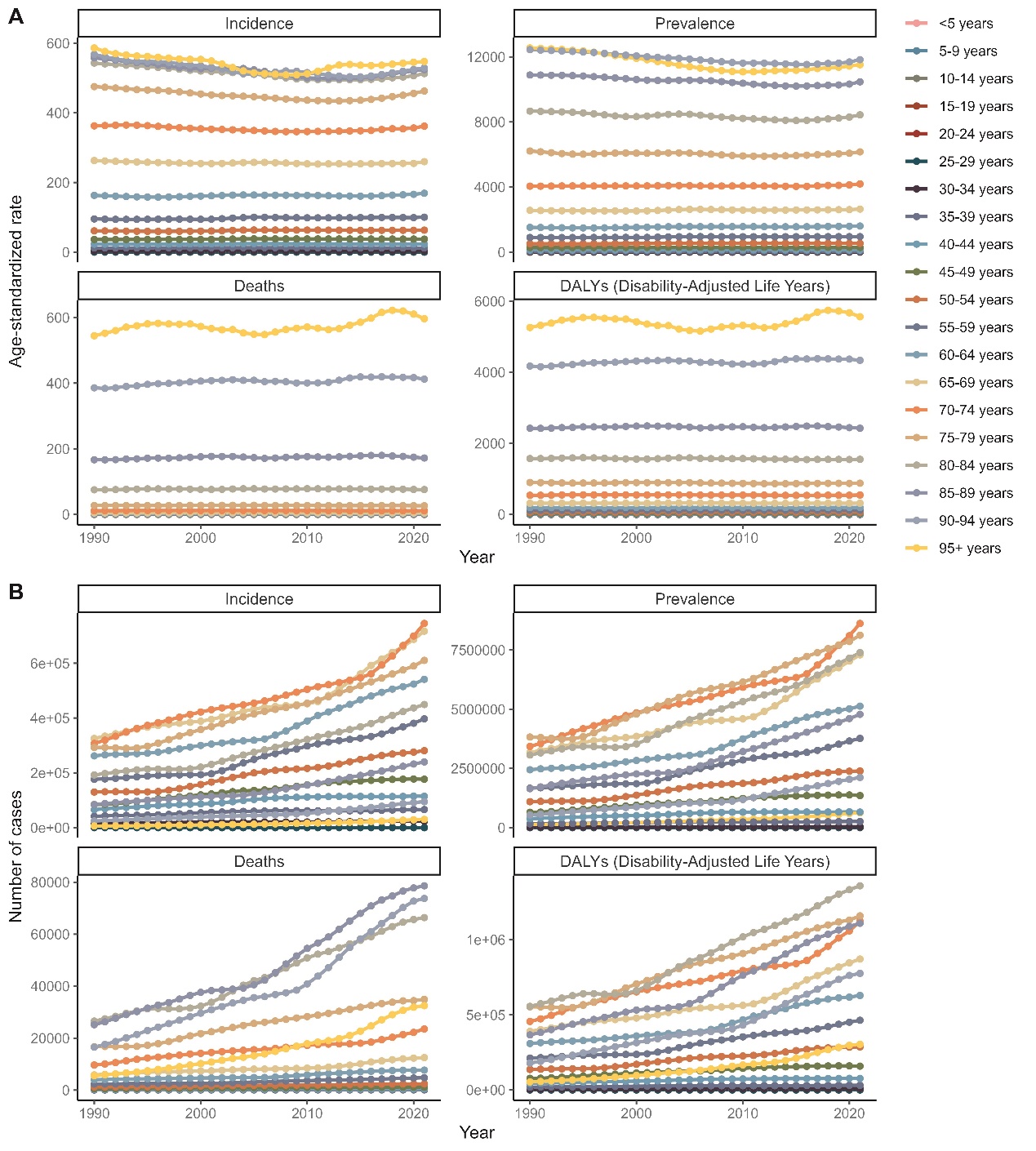


**Figure S1** Age-standardized rate and numbers of incidence, prevalence, deaths, and DALYs change curves for AF/AFL patients by ages from 1990 to 2021 (A: Age-standardized rate, B: Number of cases), AF=atrial fibrillation, AFL=Atrial flutter, DAYLs=disability-adjusted life-years


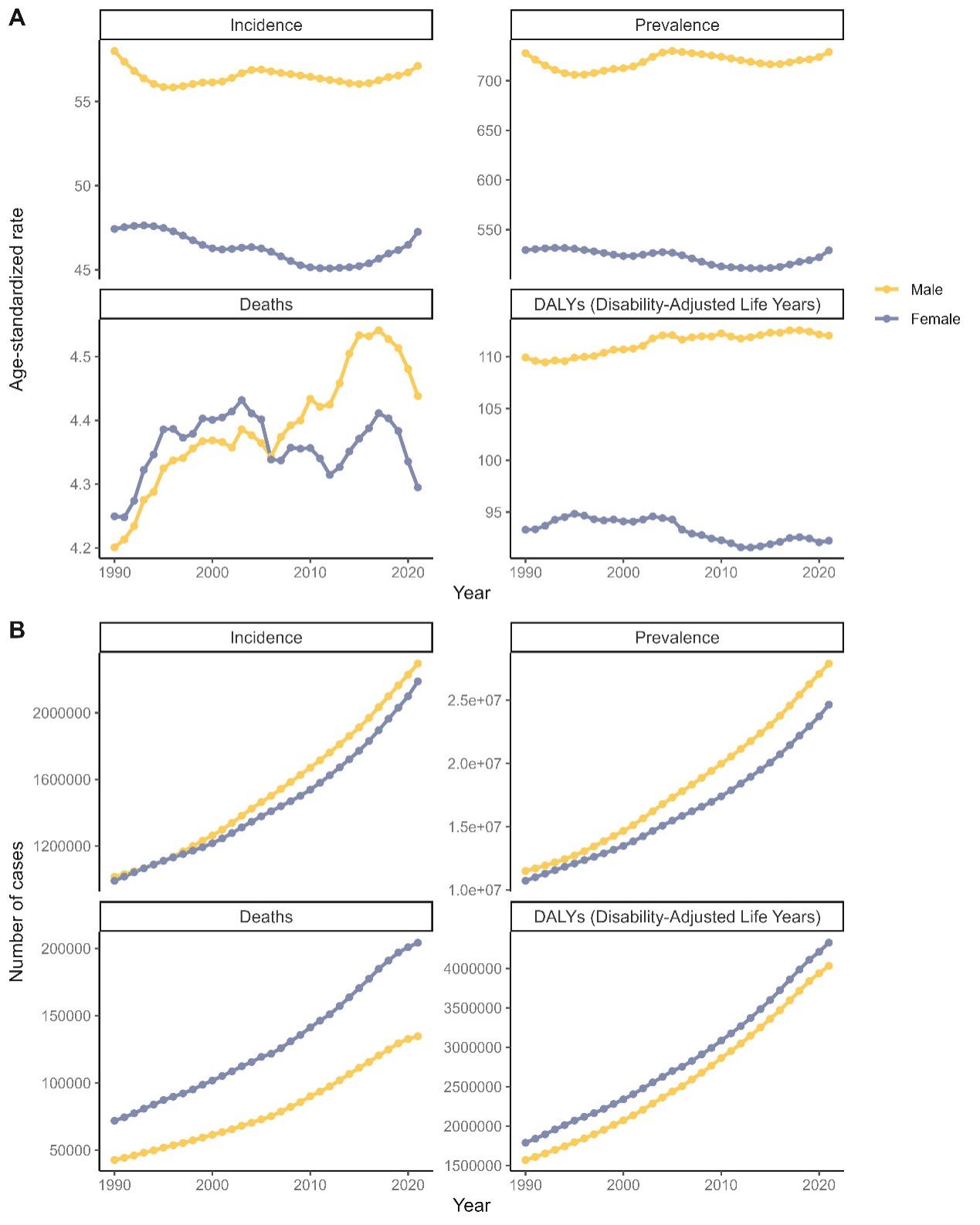


**Figure S2** Age-standardized rate and numbers of incidence, prevalence, deaths, and DALYs change curves for AF/AFL patients by sex from 1990 to 2021 (A: Age-standardized rate, B: Number of cases), AF=atrial fibrillation, AFL=Atrial flutter, DAYLs=disability-adjusted life-years


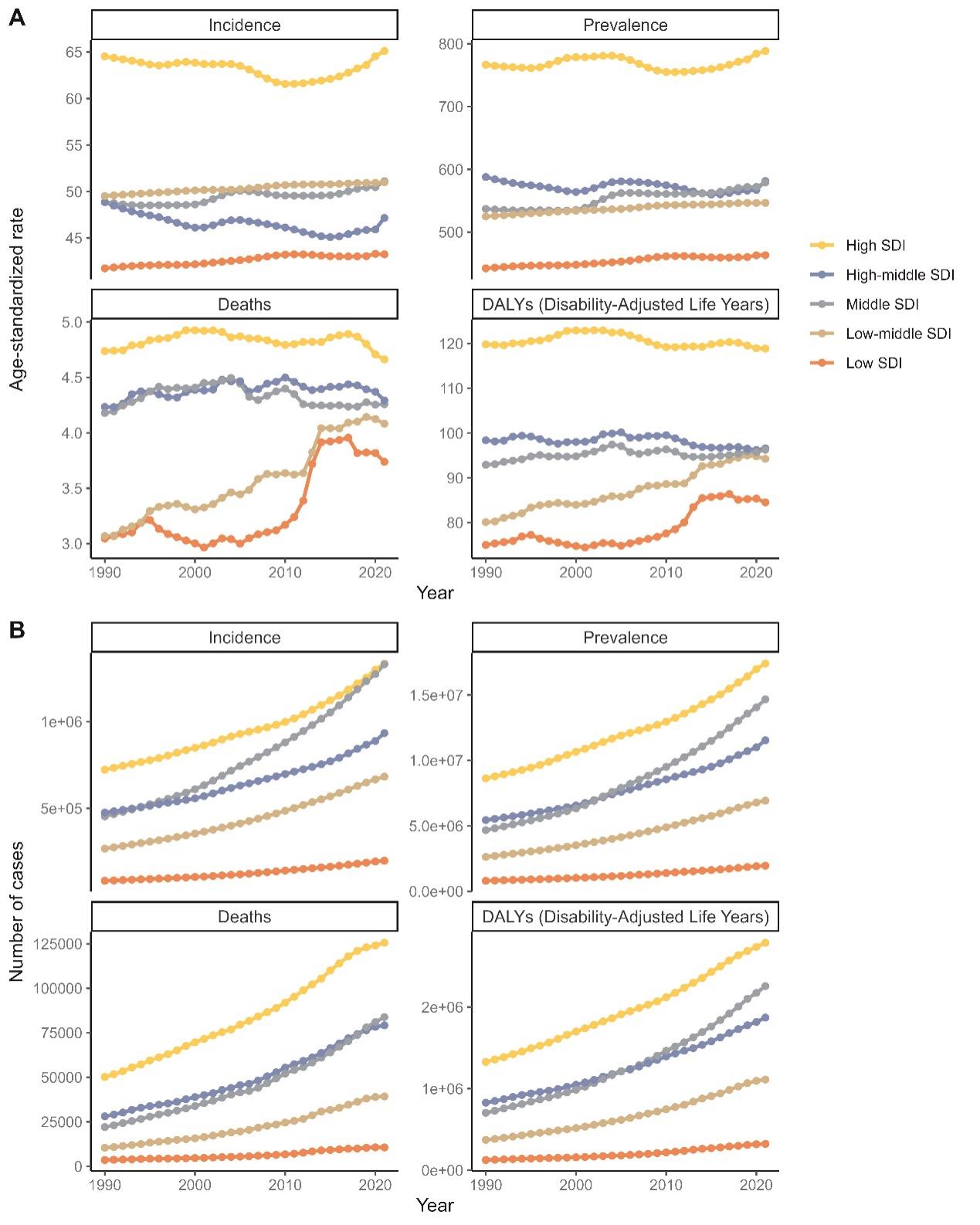


**Figure S3** Age-standardized rate and numbers of incidence, prevalence, deaths, and DALYs change curves for AF/AFL patients by SDI from 1990 to 2021 (A: Age-standardized rate, B: Number of cases), AF=atrial fibrillation, AFL=Atrial flutter, DAYLs=disability-adjusted life-years, SDI=socio-demographic index


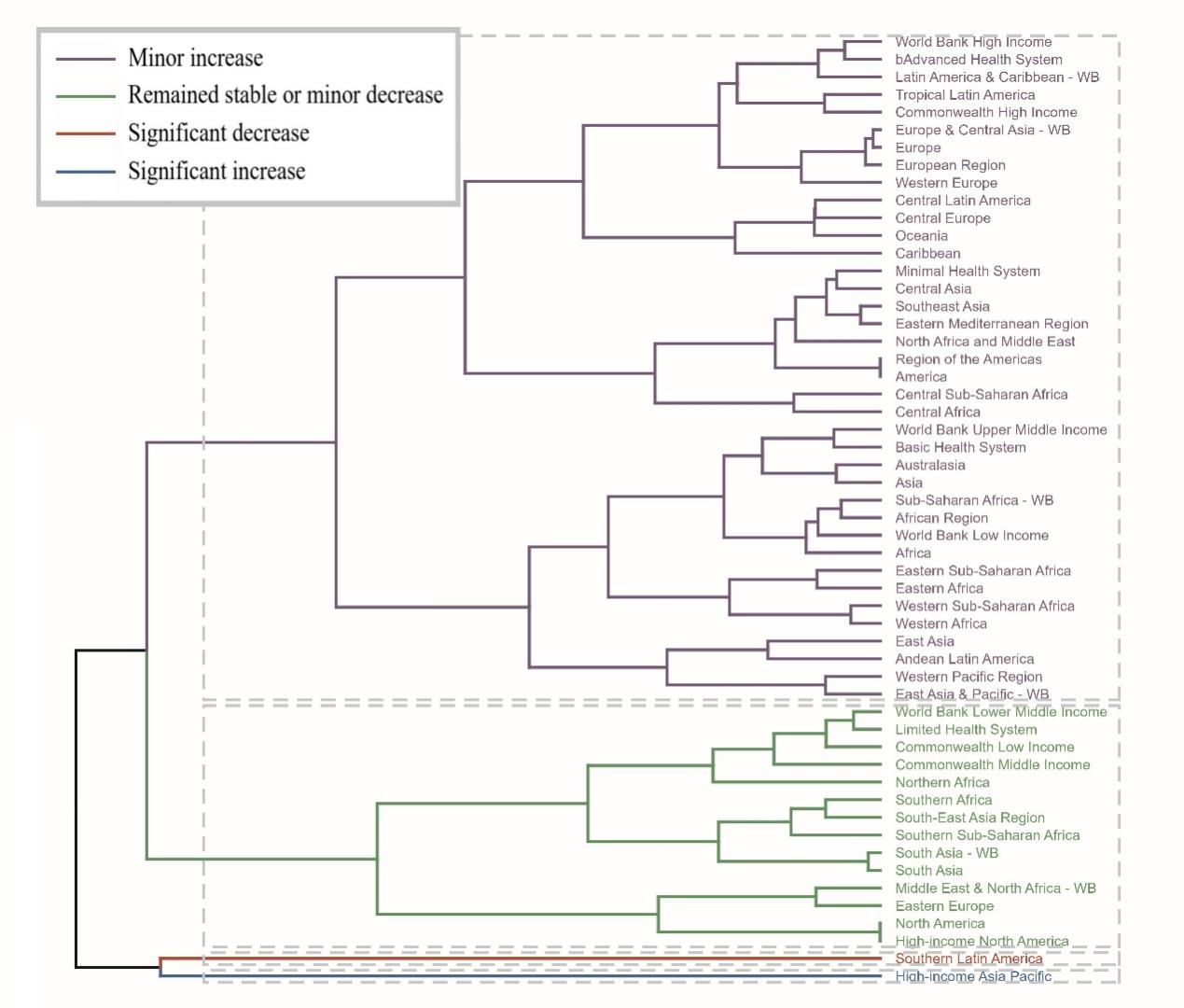


**Figure S4** The clusters of countries and territories in terms of the temporal trends related EAPC of incidence of AF/AFL, AF=atrial fibrillation, AFL=Atrial flutter, EAPC= estimated annual percentage change


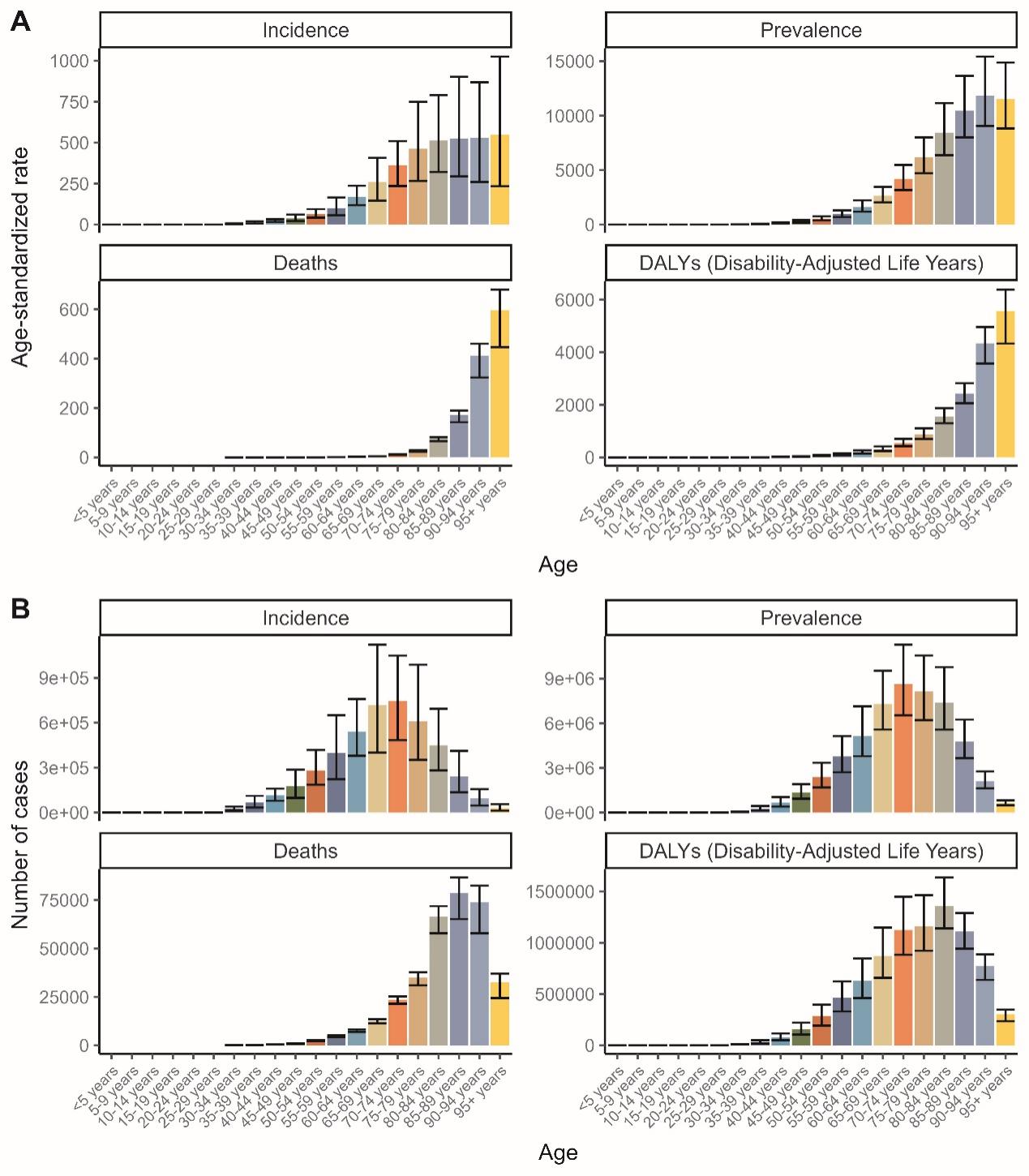


**Figure S5** Age-standardized rate and numbers of incidence, prevalence, deaths, and DALYs of AF/AFL by age, 2021, AF=atrial fibrillation, AFL=Atrial flutter, DAYLs=disability-adjusted life-years


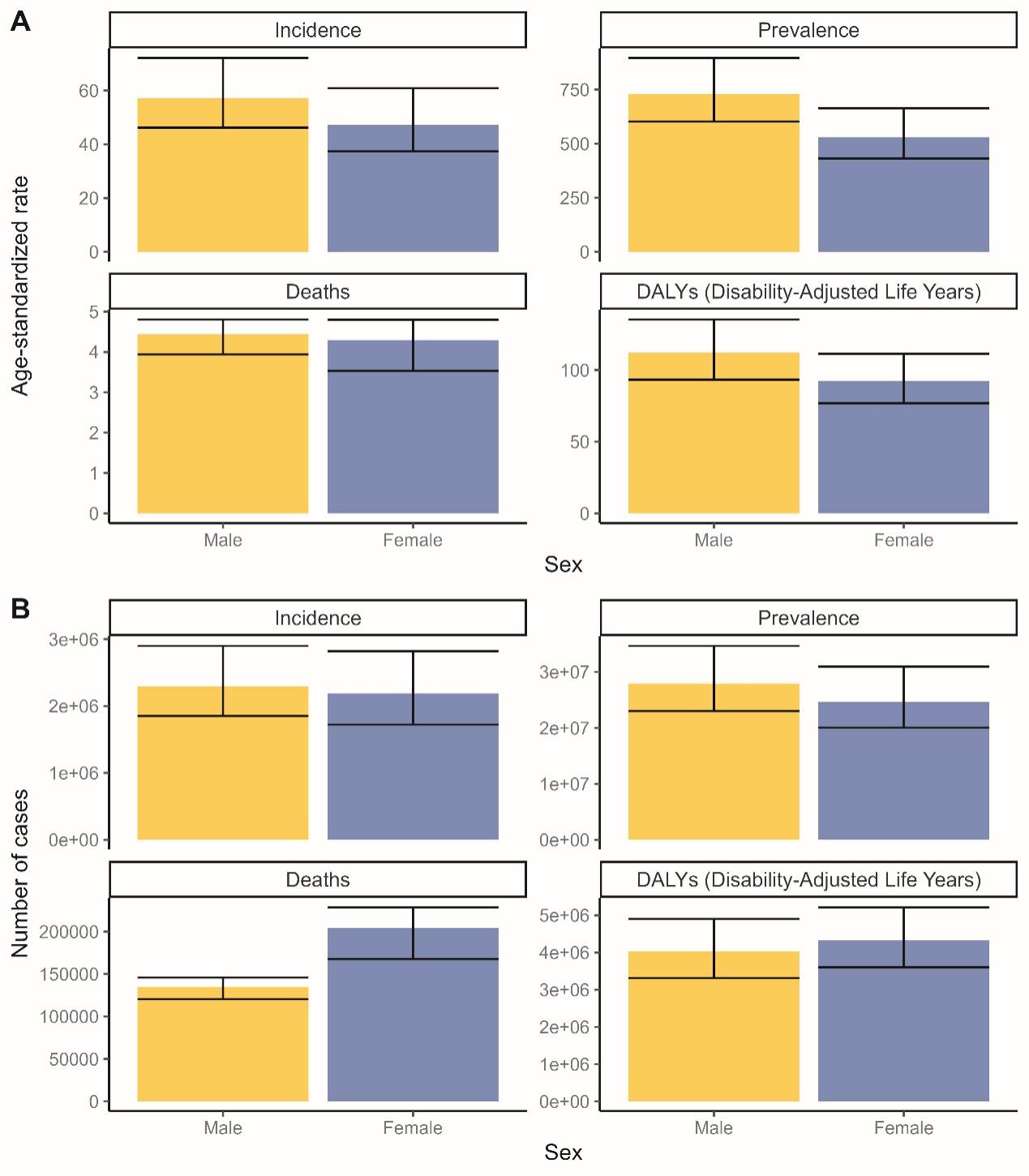


**Figure S6** Age-standardized rate and numbers of incidence, prevalence, deaths, and DALYs of AF/AFL by sex, 2021, AF=atrial fibrillation, AFL=Atrial flutter, DAYLs=disability-adjusted life-years


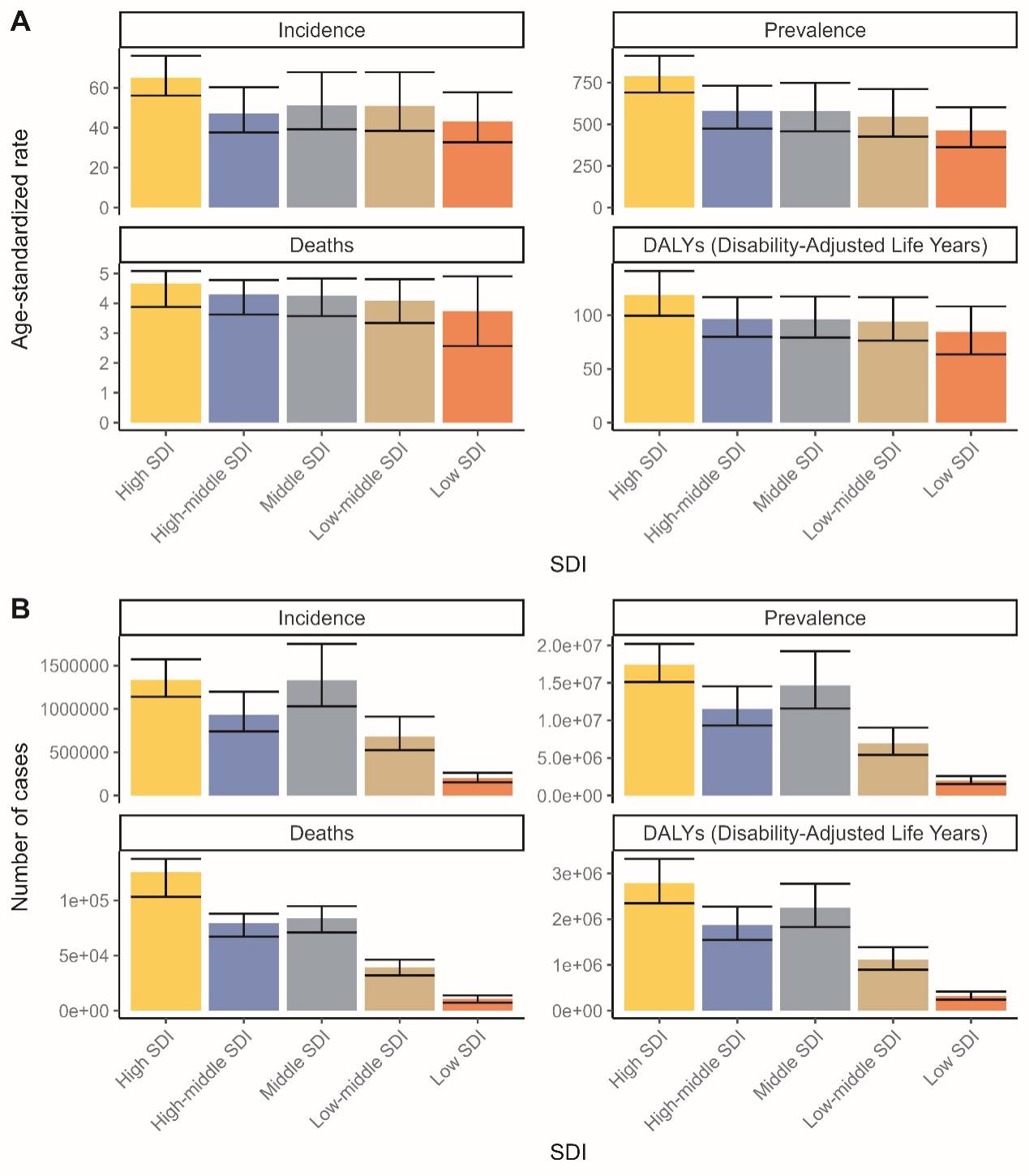


**Figure S7** Age-standardized rate and numbers of incidence, prevalence, deaths, and DALYs of AF/AFL by SDI, 2021, AF=atrial fibrillation, AFL=Atrial flutter, DAYLs=disability-adjusted life-years, SDI=socio-demographic index


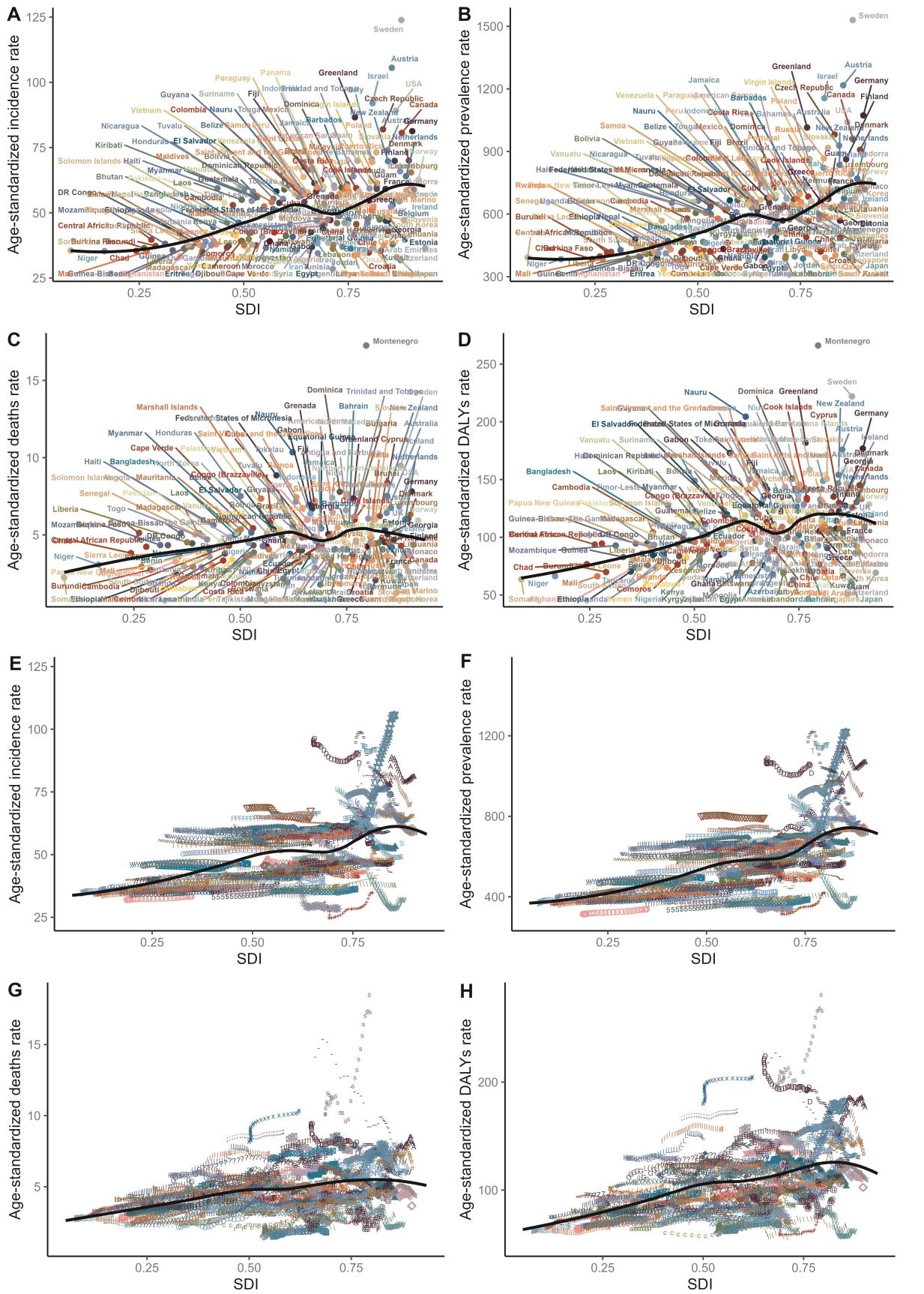


**Figure S8** Age-standardized incidence, prevalence, deaths, and DALYs rates for AF/AFL for 204 countries and territories (1,2) by SDI, 1990–2021 Expected values based on SDI and disease rates in all locations are shown as the black line, AF=atrial fibrillation, AFL=Atrial flutter, DAYLs=disability-adjusted life-years, SDI=socio-demographic index


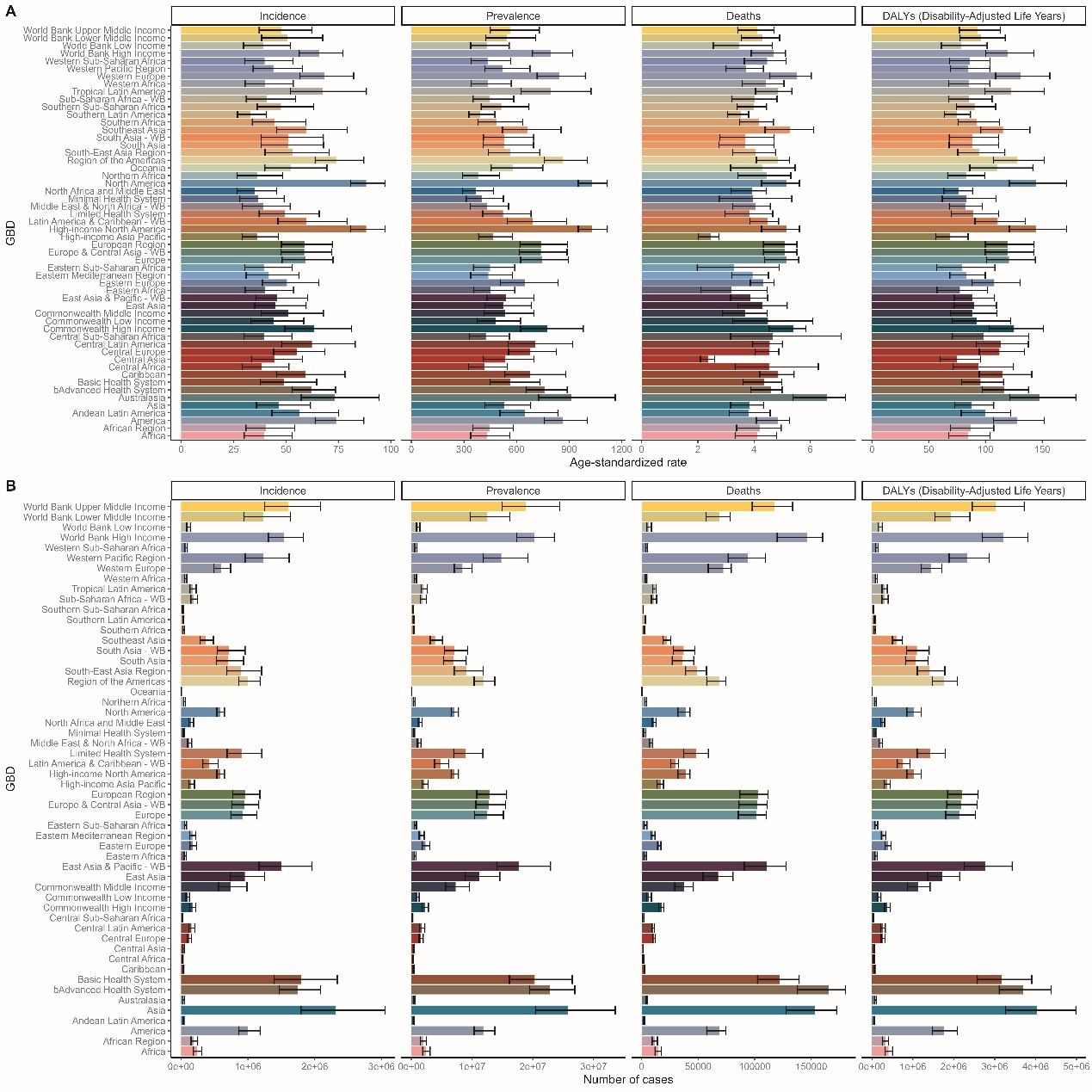


**Figure S9** Age-standardized rate and numbers of incidence, prevalence, deaths, and DALYs of AF/AFL by all GBD regions, 2021, AF=atrial fibrillation, AFL=Atrial flutter, DAYLs=disability-adjusted life-years


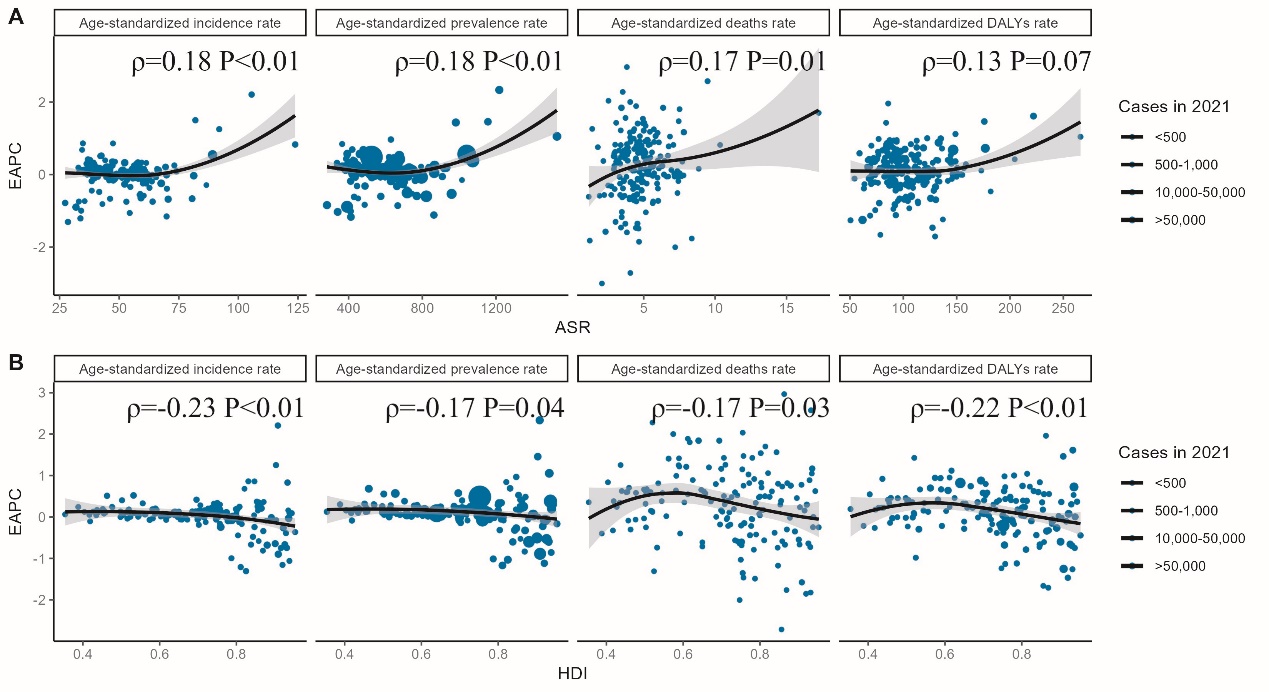


**Figure S10** The correlation between EAPC and AF/AFL ASR in 2021 (A) and HDI in 2021 (B). The circles represent countries that were available on HDI data. The size of circle is increased with the cases of AF/AFL. The ρ indices and p-values presented in (A) and (B) were derived from Pearson correlation analysis. AF=atrial fibrillation, AFL=Atrial flutter, EAPC=estimated annual percentage change, HDI=human development index


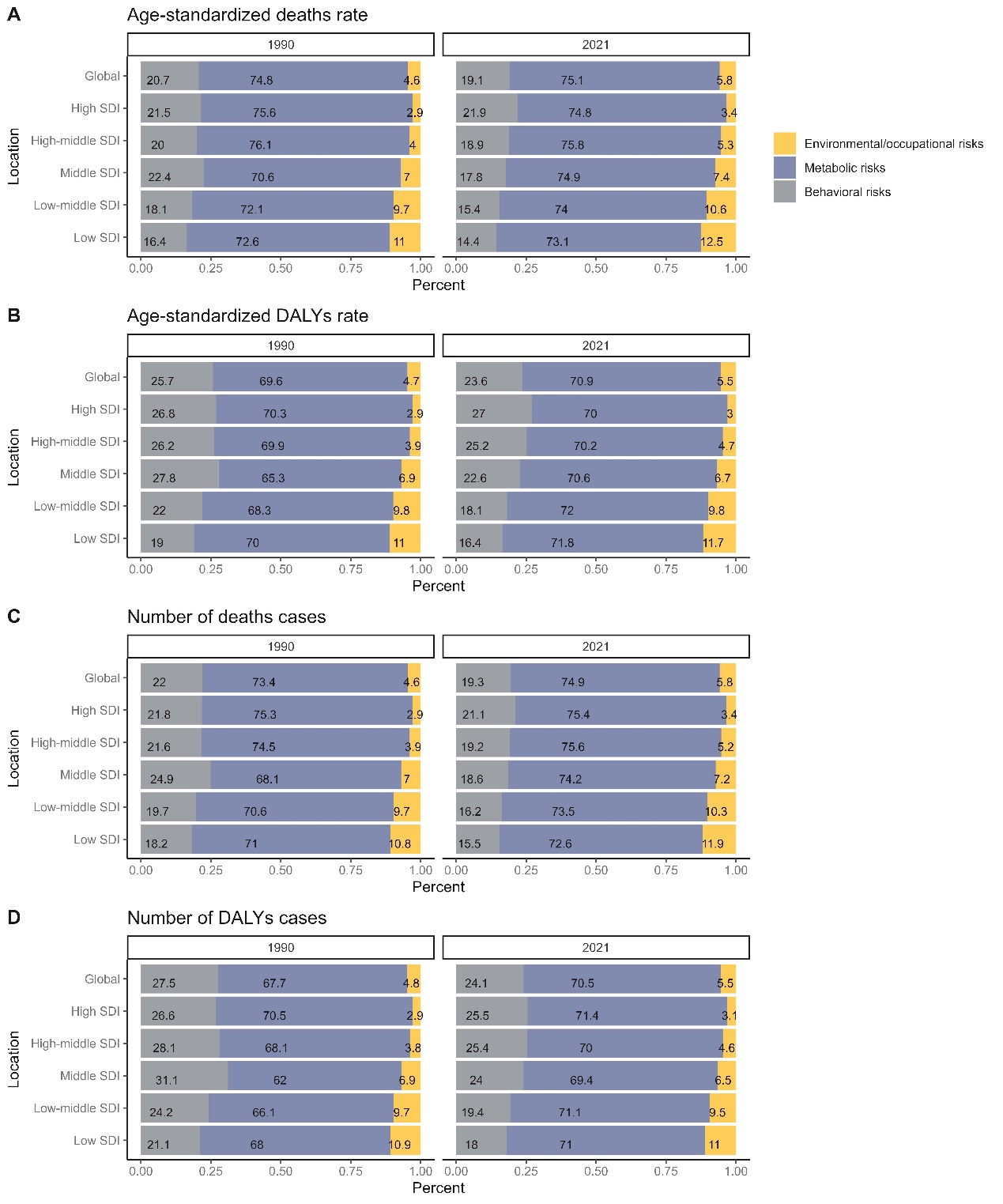


**Figure S11** Proportion of age-standardized rate and numbers of cases of deaths and DALYs due to AF/AFL attributable to three main categories risk factors for Global Burden of Disease regions by SDI, both sexes, 2021, AF=atrial fibrillation, AFL=Atrial flutter, DAYLs=disability-adjusted life-years, SDI=socio-demographic index


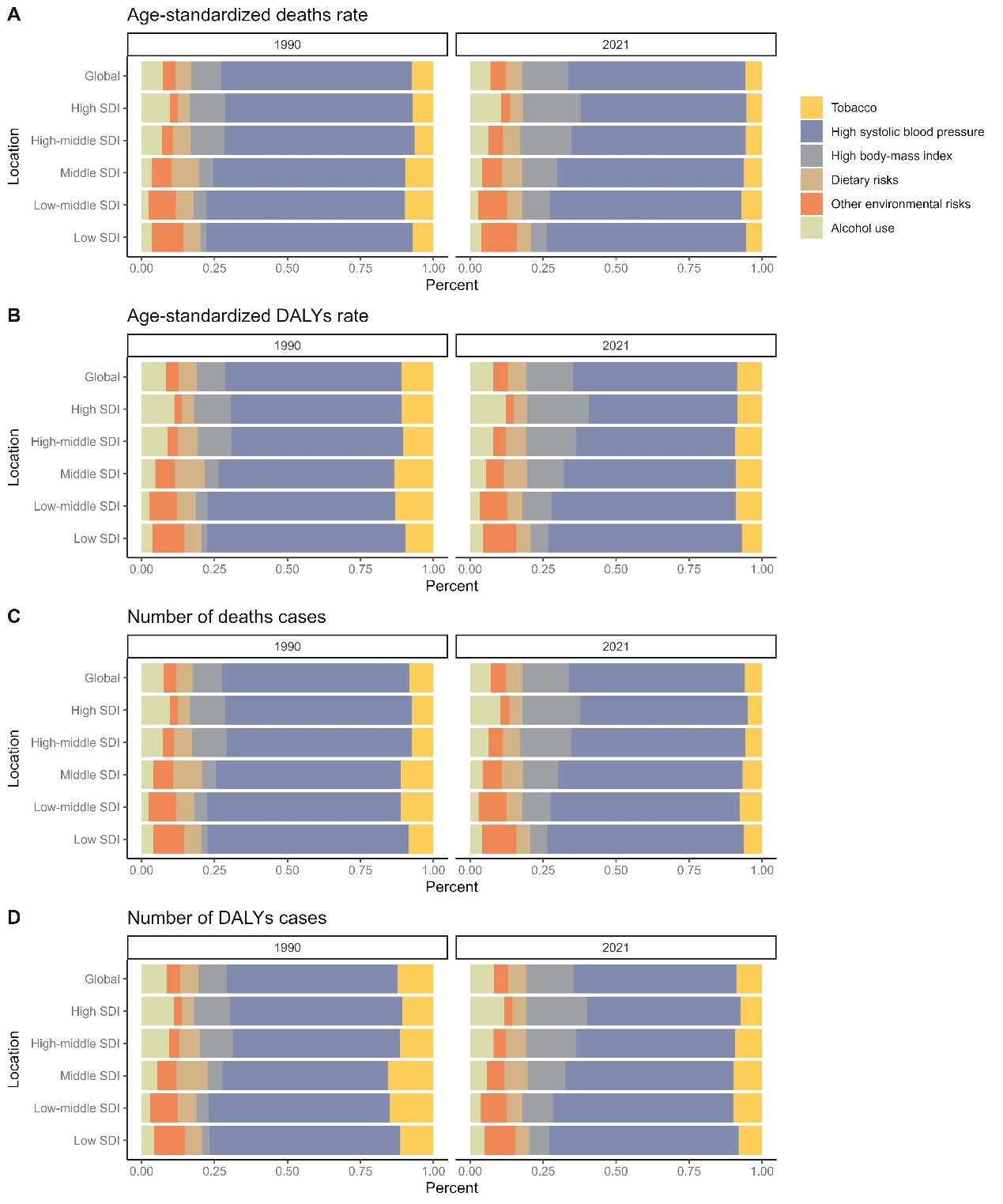


**Figure S12** Proportion of age-standardized rate and numbers of cases of deaths and DALYs due to AF/AFL attributable to five subcategories risk factors for Global Burden of Disease regions by SDI, both sexes, 2021, AF=atrial fibrillation, AFL=Atrial flutter, DAYLs=disability-adjusted life-years, SDI=socio-demographic index


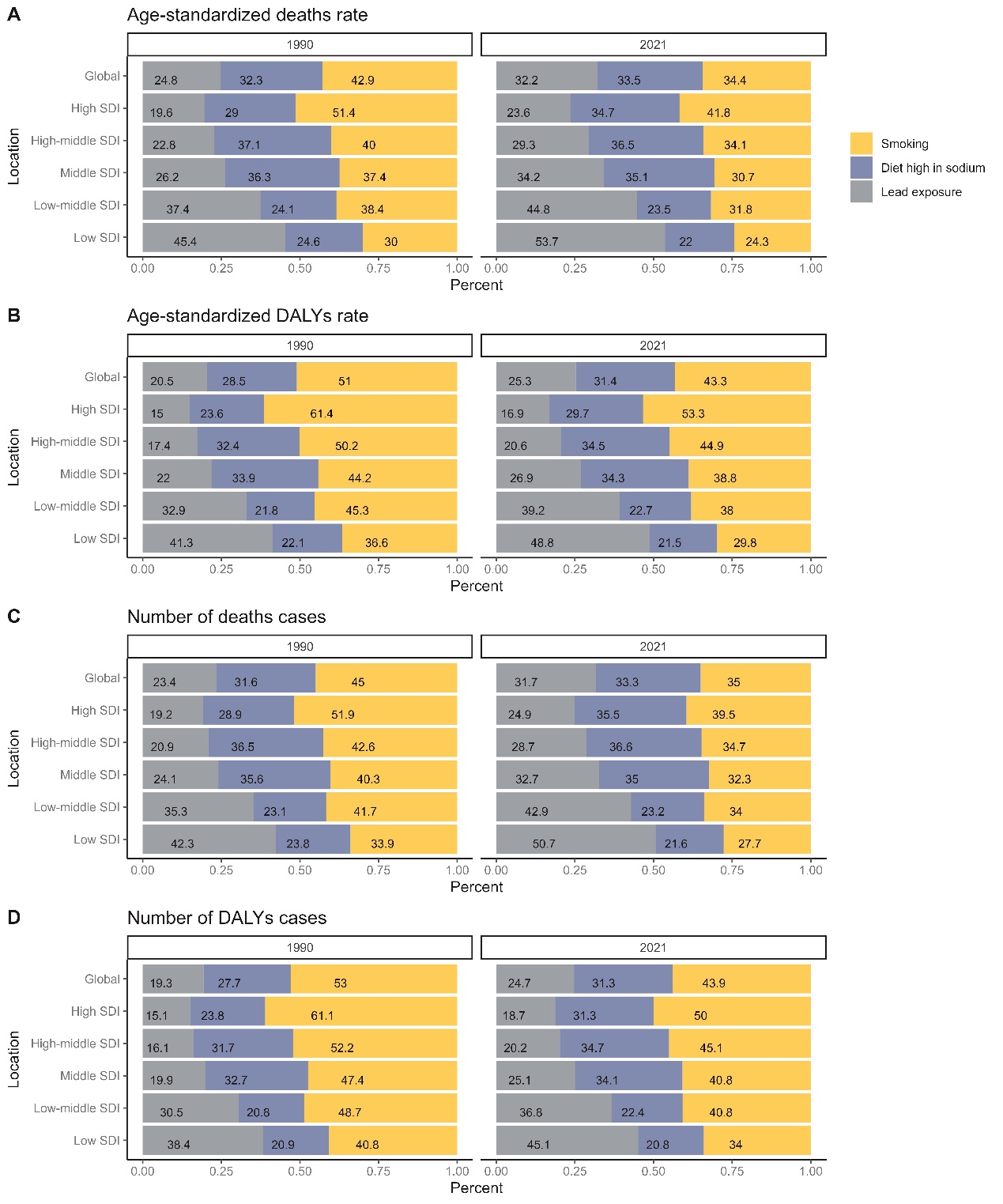


**Figure S13** Proportion of age-standardized rate and numbers of cases of deaths and DALYs due to AF/AFL attributable to three subset risk factors for Global Burden of Disease regions by SDI, both sexes, 2021, AF=atrial fibrillation, AFL=Atrial flutter, DAYLs=disability-adjusted life-years, SDI=socio-demographic index
