# Supplementary material for: Global burden of atrial fibrillation/atrial flutter and its attributable risk factors from 1990 to 2021
Source: Europace. 2024 Jul 10;26(7):euae195. doi: 10.1093/europace/euae195 (PMC11287210; doi:10.1093/europace/euae195)
Supplement: euae195_Supplementary_Data [file euae195_supplementary_data.zip › Table S1.docx]

Table S1 The incidence cases and age-standardized incidence of AF/AFL in 1990 and 2021, and its temporal trends from 1990 to 2021, by 204 countries and territories

| **Characteristics** | **Number of incidence cases in 1990** | **ASIR per 100,000**  **(95% UI)** | **Number of incidence cases in 2021** | **ASIR per 100,000**  **(95% UI)** | **1990-2021EAPC**  **(95% CI)** |
| --- | --- | --- | --- | --- | --- |
| Country |  |  |  |  |  |
| Afghanistan | 1906 (1415-2571) | 31.95 (23.7-42.95) | 2735 (2056-3602) | 33.33 (24.48-44.48) | 0.13 (0.11-0.15) |
| Albania | 995 (742-1295) | 47.94 (35.37-62.61) | 2070 (1507-2764) | 46.19 (34.59-60.4) | -0.1 (-0.13--0.07) |
| Algeria | 3727 (2696-5043) | 34.09 (24.78-46.4) | 11749 (8706-15697) | 35.37 (25.91-47.68) | 0.05 (0.02-0.07) |
| American Samoa | 11 (9-15) | 56.33 (42.71-73.79) | 27 (21-36) | 59.56 (45.61-79.26) | 0.15 (0.12-0.18) |
| Andorra | 41 (30-54) | 69.12 (51.57-91.7) | 91 (68-119) | 60.36 (45.23-78.41) | -0.58 (-0.64--0.52) |
| Angola | 1311 (1002-1729) | 39.83 (30.05-53.22) | 4344 (3391-5660) | 41.84 (31.79-55.87) | 0.18 (0.17-0.2) |
| Antigua and Barbuda | 31 (23-42) | 57.22 (43.72-75.88) | 60 (46-80) | 57.46 (43.94-75.87) | 0 (-0.01-0.01) |
| Argentina | 13363 (10066-17929) | 41.84 (31.73-55.61) | 16008 (13581-19205) | 28.5 (24.36-34.01) | -1.31 (-1.62--0.99) |
| Armenia | 1140 (872-1502) | 43.01 (32.14-56.2) | 2013 (1479-2662) | 45.65 (34.23-60.63) | 0.21 (0.19-0.23) |
| Australia | 14443 (13099-15906) | 73.34 (66.69-80.69) | 32531 (24219-42746) | 74.05 (56.85-96.39) | 0.09 (0.04-0.15) |
| Austria | 7033 (6199-8047) | 57.77 (51.28-65.36) | 18808 (17782-19775) | 105.58 (100.49-110.74) | 2.21 (2.07-2.34) |
| Azerbaijan | 2079 (1577-2727) | 43.33 (32.63-56.59) | 4617 (3523-6113) | 44.9 (33.78-58.97) | 0.14 (0.12-0.16) |
| Bahamas | 92 (70-121) | 61.88 (46.8-81.61) | 234 (181-309) | 59.29 (45.09-78.28) | -0.13 (-0.14--0.11) |
| Bahrain | 53 (41-70) | 35.35 (26.17-47.72) | 297 (229-382) | 36.29 (26.61-48.17) | 0.04 (0.01-0.07) |
| Bangladesh | 19440 (14830-25449) | 45.54 (33.71-59.79) | 60982 (45614-80831) | 46.19 (34.26-61.31) | 0.06 (0.01-0.12) |
| Barbados | 178 (128-242) | 59.42 (45.08-79.16) | 316 (237-425) | 60.95 (46.79-81.94) | 0.1 (0.08-0.12) |
| Belarus | 5642 (4239-7454) | 43.56 (32.82-56.35) | 7544 (5606-9888) | 47.24 (35.87-61.53) | 0.25 (0.21-0.29) |
| Belgium | 9867 (7416-13339) | 62.56 (47.73-83.44) | 11602 (8964-15260) | 53.01 (41.85-67.78) | -0.75 (-0.88--0.62) |
| Belize | 55 (42-73) | 59.88 (45.68-79.39) | 175 (136-229) | 60.74 (45.89-80.02) | 0.06 (0.04-0.08) |
| Benin | 657 (505-859) | 35.02 (26.78-45.86) | 1743 (1366-2273) | 36.28 (27.75-48.29) | 0.12 (0.11-0.13) |
| Bermuda | 38 (29-51) | 62.17 (47.44-82.32) | 82 (62-110) | 60.11 (45.87-79.58) | -0.12 (-0.14--0.1) |
| Bhutan | 94 (71-123) | 45.6 (33.8-60.36) | 269 (202-360) | 46.27 (34.64-61.83) | 0.04 (0.02-0.07) |
| Bolivia (Plurinational State of) | 1648 (1256-2170) | 55.11 (41.58-73.43) | 4880 (3734-6425) | 55.4 (42.03-74.02) | 0.05 (0.03-0.08) |
| Bosnia and Herzegovina | 1861 (1414-2479) | 45.79 (34.25-60.36) | 2895 (2119-3855) | 46.03 (34.49-60.17) | 0.03 (0-0.06) |
| Botswana | 201 (153-267) | 40.76 (30.74-54.2) | 581 (449-758) | 42.95 (32.63-57.14) | 0.16 (0.15-0.17) |
| Brazil | 58962 (45460-76802) | 68.76 (53.03-90.37) | 169133 (130789-219703) | 67.63 (52.17-88.49) | -0.24 (-0.29--0.19) |
| Brunei Darussalam | 48 (38-61) | 43.19 (33.56-56.47) | 130 (101-166) | 35.52 (27.3-45.61) | -0.73 (-0.8--0.67) |
| Bulgaria | 6135 (4417-8233) | 47.44 (34.84-62.38) | 6484 (4590-8604) | 45.66 (33.96-60.04) | -0.05 (-0.09--0.01) |
| Burkina Faso | 1373 (1046-1825) | 35.03 (26.64-46.61) | 3131 (2428-4105) | 36.46 (27.89-48.51) | 0.12 (0.1-0.13) |
| Burundi | 791 (607-1035) | 36.9 (28.28-48.88) | 1690 (1320-2214) | 37.32 (28.58-49.92) | 0.05 (0.04-0.06) |
| Cabo Verde | 83 (62-114) | 36.05 (27.37-48.38) | 163 (127-214) | 37.51 (28.45-50.47) | 0.13 (0.12-0.14) |
| Cambodia | 2096 (1615-2727) | 53.26 (40.7-70.29) | 5973 (4647-7812) | 52.94 (40.15-69.72) | -0.03 (-0.05--0.02) |
| Cameroon | 1413 (1076-1853) | 35.24 (26.59-47.05) | 4541 (3527-5945) | 39.23 (29.67-51.86) | 0.33 (0.26-0.4) |
| Canada | 33635 (25923-42484) | 100.87 (78.41-126.21) | 58043 (42013-77003) | 80.6 (59.82-106.65) | -0.67 (-0.78--0.55) |
| Central African Republic | 380 (287-504) | 39.89 (30.12-53.39) | 737 (569-976) | 39.69 (30.18-52.71) | -0.02 (-0.02--0.01) |
| Chad | 905 (680-1215) | 34.2 (26.02-45.78) | 1862 (1437-2449) | 35.4 (26.67-47.34) | 0.12 (0.11-0.13) |
| Chile | 4534 (3449-6005) | 46.21 (35.02-61.54) | 10713 (8108-14239) | 41.63 (31.85-55.05) | -0.48 (-0.53--0.43) |
| China | 306585 (234243-404868) | 42.63 (32.4-56.46) | 916180 (707384-1201381) | 44.92 (34.96-59.42) | 0.16 (0.08-0.25) |
| Colombia | 9701 (7498-12735) | 57.96 (44.04-77.15) | 31451 (24378-41668) | 56.71 (43.35-75.87) | -0.05 (-0.07--0.03) |
| Comoros | 63 (49-82) | 35.58 (26.93-47.29) | 170 (132-226) | 36.7 (28.01-49.08) | 0.09 (0.08-0.1) |
| Congo | 379 (286-501) | 40.82 (30.79-54.27) | 1025 (797-1332) | 42.47 (32.26-56.38) | 0.14 (0.13-0.15) |
| Cook Islands | 6 (5-8) | 53.77 (40.76-71.21) | 15 (12-20) | 59.44 (45.14-78.03) | 0.33 (0.3-0.36) |
| Costa Rica | 1106 (845-1463) | 64.47 (48.81-86.04) | 3442 (2653-4536) | 62.53 (48-82.88) | -0.12 (-0.13--0.11) |
| Croatia | 1919 (1584-2351) | 30.99 (25.33-38.17) | 3045 (2682-3466) | 34.78 (30.93-39.18) | 0.86 (0.6-1.12) |
| Cuba | 6120 (4594-8183) | 59.93 (45.39-79.13) | 11429 (8751-15178) | 58.1 (44.65-76.07) | -0.1 (-0.12--0.08) |
| Cyprus | 477 (347-625) | 55.82 (42-72.7) | 928 (720-1218) | 42.95 (34.02-55.26) | -0.81 (-0.91--0.72) |
| Czechia | 7616 (5591-10112) | 54.7 (40.9-71.71) | 17905 (14041-20271) | 81.97 (65.98-91.63) | 1.5 (1.07-1.93) |
| Côte d'Ivoire | 1300 (996-1708) | 37.12 (27.91-49.32) | 3940 (3081-5145) | 38.46 (29.28-51.82) | 0.11 (0.1-0.12) |
| Democratic People's Republic of Korea | 7060 (5522-9165) | 49.69 (38.11-65.61) | 15152 (11772-20144) | 48.09 (37.23-63.29) | -0.13 (-0.15--0.12) |
| Democratic Republic of the Congo | 5372 (4076-7087) | 40.23 (30.46-53.18) | 12383 (9617-16381) | 38.47 (28.94-51.58) | -0.18 (-0.19--0.17) |
| Denmark | 5649 (4210-7572) | 68.96 (52.48-89.77) | 7912 (5907-10323) | 70.03 (54.45-89.03) | -0.39 (-0.57--0.21) |
| Djibouti | 43 (34-56) | 34.83 (26.74-46.37) | 224 (175-293) | 37.38 (28.63-49.61) | 0.25 (0.23-0.26) |
| Dominica | 35 (26-47) | 58.83 (45.26-78.42) | 49 (37-65) | 59.66 (45.28-78.68) | 0.06 (0.05-0.08) |
| Dominican Republic | 2106 (1609-2774) | 59.61 (45.77-78.47) | 5823 (4490-7736) | 58.97 (45.15-78.8) | -0.05 (-0.06--0.04) |
| Ecuador | 2792 (2141-3638) | 54.84 (42-72.39) | 8791 (6715-11724) | 54.15 (41.24-71.88) | 0 (-0.02-0.02) |
| Egypt | 7316 (5543-9606) | 33.01 (24.14-44.24) | 19786 (14849-25927) | 36.92 (27.14-49.42) | 0.42 (0.39-0.44) |
| El Salvador | 1707 (1311-2234) | 58.97 (44.52-78.27) | 3647 (2778-4768) | 58.15 (44.23-76.71) | -0.04 (-0.05--0.03) |
| Equatorial Guinea | 66 (50-87) | 39.36 (29.69-53.03) | 205 (159-266) | 44.12 (33.56-58.64) | 0.39 (0.38-0.41) |
| Eritrea | 326 (253-428) | 33.94 (26.03-45.24) | 883 (684-1162) | 35.13 (26.99-47.06) | 0.11 (0.11-0.12) |
| Estonia | 871 (648-1141) | 42.66 (32.14-54.94) | 1149 (859-1498) | 46.91 (35.52-60.22) | 0.36 (0.32-0.4) |
| Eswatini | 107 (82-140) | 41.67 (31.37-55.9) | 217 (167-287) | 43.34 (32.97-57.62) | 0.12 (0.11-0.14) |
| Ethiopia | 6365 (4844-8392) | 37.03 (28.2-49.29) | 17579 (13804-22707) | 42.11 (32.38-55.88) | 0.51 (0.45-0.57) |
| Fiji | 176 (136-229) | 55.98 (42.17-74.75) | 428 (326-563) | 61.36 (46.49-80.16) | 0.32 (0.29-0.35) |
| Finland | 6773 (4959-8593) | 94.77 (70.63-119.71) | 8476 (6391-11009) | 69.89 (55.04-86.68) | -1.16 (-1.25--1.06) |
| France | 57571 (42070-77071) | 68.85 (51.4-90.39) | 78372 (57303-105675) | 59.15 (44.34-78.68) | -0.64 (-0.71--0.57) |
| Gabon | 214 (162-288) | 40.53 (30.35-54.15) | 407 (313-532) | 42.53 (32.55-56.71) | 0.15 (0.15-0.16) |
| Gambia | 116 (89-151) | 36.42 (27.6-48.08) | 342 (265-451) | 37.4 (28.57-50.28) | 0.06 (0.03-0.09) |
| Georgia | 2950 (2187-3910) | 47.31 (35.38-61.93) | 2830 (2114-3690) | 47.21 (35.92-61.51) | -0.06 (-0.08--0.03) |
| Germany | 101466 (75243-135577) | 79.75 (60.18-104.73) | 140086 (122558-158105) | 81.28 (72.34-89.74) | -0.03 (-0.18-0.12) |
| Ghana | 2024 (1557-2654) | 36.14 (27.28-48.06) | 6048 (4667-7938) | 39.08 (29.56-51.95) | 0.17 (0.1-0.24) |
| Greece | 8833 (7049-11378) | 57.29 (45.98-72.87) | 12367 (9012-16893) | 54.67 (41-71.66) | -0.34 (-0.55--0.13) |
| Greenland | 30 (23-39) | 96.28 (72.66-125.85) | 60 (44-79) | 86.59 (65.04-114.49) | -0.29 (-0.37--0.21) |
| Grenada | 43 (32-58) | 58.26 (44.46-77.85) | 65 (49-86) | 58.42 (44.63-77.91) | 0 (-0.04-0.03) |
| Guam | 37 (28-47) | 53.53 (41.02-69.64) | 126 (97-164) | 59.14 (45.26-77.38) | 0.32 (0.29-0.35) |
| Guatemala | 1768 (1358-2343) | 55.48 (41.89-73.71) | 6076 (4623-8090) | 56.47 (42.71-75.76) | 0.13 (0.09-0.17) |
| Guinea | 1068 (809-1412) | 34.48 (26.09-45.94) | 1867 (1435-2453) | 35.62 (26.88-47.94) | 0.11 (0.1-0.12) |
| Guinea-Bissau | 124 (93-164) | 35.6 (26.79-46.54) | 233 (180-308) | 36.54 (27.84-49.11) | 0.08 (0.07-0.09) |
| Guyana | 219 (168-288) | 61.15 (46.34-81.74) | 364 (277-481) | 59.63 (45.02-79.68) | -0.06 (-0.08--0.04) |
| Haiti | 1745 (1328-2272) | 59.81 (45.73-79.63) | 3950 (3038-5216) | 59.42 (44.98-79.53) | 0 (-0.02-0.02) |
| Honduras | 1159 (896-1522) | 60.01 (46.1-79.46) | 3592 (2759-4716) | 58.92 (45.29-77.41) | -0.07 (-0.07--0.06) |
| Hungary | 8383 (6153-11236) | 55.95 (41.54-73.85) | 8870 (6408-11720) | 46.18 (34.66-60.55) | -0.67 (-0.72--0.61) |
| Iceland | 175 (129-230) | 60.61 (45.01-78.8) | 375 (296-464) | 65.91 (52.74-81.71) | 0.16 (0.08-0.24) |
| India | 198419 (151420-263224) | 50.27 (37.44-66.79) | 570121 (430165-762853) | 51.45 (38.36-68.54) | 0.09 (0.08-0.1) |
| Indonesia | 55881 (43453-72892) | 64.43 (49.08-85.9) | 145423 (112648-190123) | 66.36 (50.84-87.95) | 0.12 (0.11-0.13) |
| Iran (Islamic Republic of) | 8136 (6132-10787) | 37.34 (27.62-50.2) | 29418 (22665-38723) | 40.56 (30-54.38) | 0.2 (0.15-0.24) |
| Iraq | 2717 (2025-3570) | 36.58 (26.52-48.72) | 8151 (6210-10703) | 38.36 (28.13-51.24) | 0.13 (0.12-0.15) |
| Ireland | 2713 (1936-3616) | 64.43 (47.57-84.46) | 4292 (3141-5685) | 54.42 (40.84-71.55) | -1.06 (-1.21--0.91) |
| Israel | 3551 (2614-4680) | 70.89 (53.65-92.18) | 11110 (8995-12779) | 91.98 (75.23-105.16) | 1.25 (0.97-1.53) |
| Italy | 68691 (50618-91262) | 76.05 (57.71-99.91) | 99307 (72177-134792) | 70.54 (52.49-92.64) | -0.68 (-0.83--0.53) |
| Jamaica | 1041 (786-1388) | 57.72 (44.41-76.49) | 1851 (1423-2396) | 59.1 (44.9-77.87) | 0.1 (0.07-0.13) |
| Japan | 70805 (54525-93790) | 42.03 (32.59-55.25) | 107285 (77975-144807) | 31.86 (24.84-41.91) | -0.9 (-1.18--0.62) |
| Jordan | 386 (295-505) | 34.36 (25.04-46.19) | 2472 (1878-3282) | 37 (27.48-49.67) | 0.22 (0.21-0.24) |
| Kazakhstan | 5935 (4511-7796) | 48.19 (36.41-63.55) | 8829 (6626-11519) | 48.85 (37.09-63.65) | 0.03 (0-0.06) |
| Kenya | 3129 (2441-4088) | 41.04 (31.44-54.8) | 8973 (7020-11663) | 42.21 (32.33-56.29) | 0.09 (0.08-0.1) |
| Kiribati | 17 (13-23) | 55.34 (41.81-73.56) | 35 (27-46) | 55.96 (42.42-75.07) | 0.03 (0.01-0.05) |
| Kuwait | 188 (147-241) | 35.46 (25.71-47.38) | 1013 (804-1301) | 37.87 (27.94-50.89) | 0.19 (0.13-0.24) |
| Kyrgyzstan | 1203 (909-1595) | 41.46 (31-54.33) | 1957 (1491-2572) | 41.13 (31.43-53.83) | -0.06 (-0.07--0.05) |
| Lao People's Democratic Republic | 1000 (757-1324) | 55.76 (42.56-74.12) | 2333 (1816-3027) | 54.97 (41.92-72.84) | -0.05 (-0.07--0.03) |
| Latvia | 1446 (1077-1905) | 40.6 (30.59-52.86) | 1701 (1429-1990) | 47.4 (40.34-54.56) | 0.86 (0.67-1.05) |
| Lebanon | 650 (494-855) | 33.33 (24.8-44.87) | 2244 (1665-3001) | 36.25 (26.97-48.31) | 0.3 (0.29-0.31) |
| Lesotho | 300 (227-402) | 38.22 (28.75-50.95) | 387 (293-514) | 39.92 (30.15-53.63) | 0.13 (0.12-0.13) |
| Liberia | 392 (295-518) | 36.68 (27.93-49.2) | 726 (570-941) | 37.02 (27.98-49.14) | 0.04 (0.03-0.05) |
| Libya | 614 (465-806) | 35.68 (26.09-47.9) | 1777 (1361-2315) | 37.69 (27.8-50.68) | 0.15 (0.13-0.17) |
| Lithuania | 1975 (1485-2581) | 43.97 (33.46-57.27) | 2548 (1918-3354) | 48.23 (36.45-62.39) | 0.35 (0.31-0.4) |
| Luxembourg | 380 (310-475) | 67.74 (56.09-83.07) | 681 (580-770) | 65.48 (56.11-73.74) | -0.08 (-0.11--0.05) |
| Madagascar | 1630 (1260-2145) | 35.21 (27.02-47.17) | 3804 (2959-5017) | 37.01 (28.34-48.94) | 0.18 (0.16-0.19) |
| Malawi | 1267 (975-1639) | 36.49 (27.89-48.03) | 2719 (2109-3516) | 39.29 (30.1-52.01) | 0.23 (0.21-0.26) |
| Malaysia | 5041 (3955-6559) | 57.12 (43.61-75.3) | 15962 (12194-20998) | 58.73 (44.38-78.66) | 0.2 (0.16-0.24) |
| Maldives | 42 (32-55) | 53.66 (40.67-71.29) | 181 (145-233) | 54.1 (41.34-71.72) | -0.01 (-0.03-0) |
| Mali | 1193 (910-1586) | 33.84 (25.66-45.78) | 2815 (2156-3713) | 35.17 (26.68-47.05) | 0.13 (0.12-0.14) |
| Malta | 251 (181-332) | 57.92 (42.47-76.67) | 512 (438-589) | 51.15 (44.57-58) | -0.09 (-0.38-0.2) |
| Marshall Islands | 7 (6-10) | 51.39 (39.1-67.49) | 16 (12-21) | 52.48 (40.09-69.43) | 0.05 (0.03-0.07) |
| Mauritania | 330 (249-441) | 35.53 (27.05-47.76) | 767 (589-1011) | 37.53 (28.39-50.3) | 0.18 (0.17-0.2) |
| Mauritius | 377 (291-492) | 56.64 (43.25-75.1) | 1022 (779-1353) | 56.83 (43.14-75.26) | -0.1 (-0.15--0.05) |
| Mexico | 26349 (20418-35014) | 65.18 (50.08-86.54) | 82432 (63607-109361) | 66.4 (50.87-88.29) | 0.11 (0.09-0.12) |
| Micronesia (Federated States of) | 24 (18-31) | 54.03 (41.13-71.19) | 35 (27-46) | 53.81 (41.12-71.15) | -0.02 (-0.03--0.01) |
| Monaco | 47 (34-63) | 66.75 (49.53-87.83) | 55 (40-74) | 58.82 (44.19-76.65) | -0.53 (-0.59--0.47) |
| Mongolia | 450 (338-593) | 44.09 (33.03-57.99) | 1034 (782-1341) | 45.64 (34.27-60.09) | 0.1 (0.09-0.12) |
| Montenegro | 313 (235-408) | 50.48 (37.49-65.94) | 484 (353-637) | 47.55 (35.29-62.08) | -0.17 (-0.2--0.14) |
| Morocco | 4690 (3492-6273) | 35.71 (26-48) | 11738 (8720-15856) | 36.59 (26.88-49.55) | 0 (-0.03-0.03) |
| Mozambique | 1958 (1514-2574) | 35.96 (27.52-48.05) | 3914 (3015-5091) | 38.13 (28.99-50.32) | 0.17 (0.15-0.19) |
| Myanmar | 11700 (8930-15322) | 57.5 (43.4-76.58) | 25401 (19548-33339) | 55.75 (42.05-74.46) | -0.1 (-0.11--0.1) |
| Namibia | 233 (174-308) | 40.47 (30.73-53.77) | 515 (390-681) | 40.81 (31.04-55.47) | 0 (-0.01-0.01) |
| Nauru | 2 (2-3) | 57.79 (43.81-77.26) | 3 (2-4) | 60.59 (45.99-80.04) | 0.09 (0.06-0.11) |
| Nepal | 3700 (2805-4891) | 45.84 (34.18-61) | 9675 (7232-12862) | 44.58 (33.43-59.27) | -0.07 (-0.13--0.01) |
| Netherlands | 14708 (13196-16558) | 72.21 (64.26-80.62) | 22455 (18709-27075) | 64.02 (54.48-75.58) | -0.75 (-0.89--0.6) |
| New Zealand | 2848 (2164-3675) | 71.08 (54.87-91.02) | 5817 (4707-7311) | 69.78 (57.18-86.67) | -0.03 (-0.11-0.06) |
| Nicaragua | 866 (669-1143) | 59.26 (44.92-78.79) | 2773 (2128-3642) | 58.13 (44.01-77.39) | -0.06 (-0.08--0.05) |
| Niger | 846 (649-1123) | 34.61 (26.15-46.54) | 2620 (1999-3479) | 35.28 (26.73-47.18) | 0.06 (0.05-0.08) |
| Nigeria | 15157 (11648-20152) | 37.27 (28.27-50.01) | 35723 (28008-46500) | 42.52 (32.42-56.76) | 0.52 (0.47-0.57) |
| Niue | 1 (1-2) | 56.96 (43.31-75.64) | 1 (1-2) | 60.47 (46.2-79.8) | 0.16 (0.13-0.19) |
| North Macedonia | 914 (683-1207) | 48.59 (36.06-64.08) | 1603 (1158-2145) | 45.77 (33.88-60.43) | -0.16 (-0.2--0.13) |
| Northern Mariana Islands | 9 (7-11) | 56.57 (43.15-75.34) | 28 (21-37) | 58.21 (44.24-76.14) | 0.07 (0.05-0.1) |
| Norway | 4758 (3431-6405) | 69.29 (52.99-90.88) | 6082 (4530-8012) | 62.72 (47.43-82.17) | -0.37 (-0.4--0.33) |
| Oman | 176 (134-227) | 28.99 (21.37-38.68) | 643 (504-829) | 34.43 (25.27-46.32) | 0.68 (0.61-0.75) |
| Pakistan | 27497 (20887-36777) | 53.26 (39.62-71.06) | 57874 (44289-75861) | 53.85 (40.3-71.6) | 0.07 (0.06-0.08) |
| Palau | 5 (4-6) | 53.39 (40.57-71.18) | 12 (9-16) | 56.92 (43.46-75.08) | 0.19 (0.16-0.22) |
| Palestine | 252 (189-338) | 31.95 (23.55-42.92) | 749 (570-982) | 33.18 (24.4-44.66) | 0.07 (0.06-0.09) |
| Panama | 870 (667-1153) | 59.85 (45.7-79.52) | 2656 (2054-3532) | 59.92 (46.23-80.07) | 0.03 (0.01-0.05) |
| Papua New Guinea | 751 (581-992) | 49.6 (37.99-65.71) | 2219 (1744-2890) | 49.67 (38.29-65.85) | 0.01 (-0.01-0.02) |
| Paraguay | 1475 (1135-1909) | 67.17 (52.06-88.01) | 3697 (2878-4841) | 63.72 (49.18-84.12) | -0.19 (-0.22--0.16) |
| Peru | 6218 (4868-8165) | 54.04 (41.36-71.29) | 19333 (15032-25246) | 57.88 (44.6-76.58) | 0.34 (0.28-0.39) |
| Philippines | 15964 (12390-20941) | 59.63 (45.31-79.4) | 45632 (35428-59598) | 60.3 (45.81-80.23) | 0.01 (0-0.02) |
| Poland | 23417 (17441-30849) | 53.07 (40-69.57) | 50025 (36265-66677) | 69.46 (51.87-90.62) | 0.21 (-0.05-0.46) |
| Portugal | 9401 (6872-12569) | 65.52 (49.07-86.59) | 14593 (11898-18076) | 59.99 (49.76-72.76) | -0.24 (-0.32--0.16) |
| Puerto Rico | 2259 (1719-3011) | 62.11 (47.9-82.27) | 4460 (3243-5952) | 61.38 (45.95-80.92) | -0.04 (-0.06--0.02) |
| Qatar | 36 (29-46) | 35.2 (25.6-46.99) | 366 (283-474) | 37.6 (27.56-50.65) | 0.15 (0.12-0.19) |
| Republic of Korea | 14766 (11525-18924) | 49.39 (38.37-64.76) | 42194 (34817-52133) | 47.21 (39.34-57.49) | -0.15 (-0.32-0.02) |
| Republic of Moldova | 1925 (1459-2517) | 43.01 (32.7-55.93) | 2822 (2114-3649) | 47.44 (36.08-60.91) | 0.36 (0.35-0.38) |
| Romania | 13355 (9778-17808) | 47.05 (34.77-61.61) | 12515 (10602-15109) | 33.74 (29.07-40.1) | -1.21 (-1.47--0.95) |
| Russian Federation | 86356 (65246-113303) | 47.79 (36.77-62.21) | 125499 (94653-165367) | 52.32 (40.22-68.2) | 0.33 (0.25-0.4) |
| Rwanda | 949 (737-1237) | 37.59 (28.8-49.6) | 2248 (1751-2936) | 37.96 (29.04-50) | 0 (-0.01-0.01) |
| Saint Kitts and Nevis | 23 (16-31) | 59.61 (45.54-78.91) | 39 (29-51) | 58.19 (44.27-77.42) | -0.11 (-0.14--0.08) |
| Saint Lucia | 54 (40-72) | 62.01 (47.82-82.34) | 143 (109-187) | 59.78 (45.19-77.74) | -0.07 (-0.09--0.04) |
| Saint Vincent and the Grenadines | 40 (30-54) | 57.27 (43.51-76.79) | 84 (64-112) | 59.11 (44.99-78.29) | 0.1 (0.09-0.11) |
| Samoa | 43 (34-57) | 56.53 (43.2-75.2) | 77 (60-102) | 57.07 (43.2-75.97) | 0 (-0.02-0.02) |
| San Marino | 24 (18-32) | 67.3 (50.42-87.53) | 41 (30-54) | 58.5 (43.3-76.78) | -0.56 (-0.6--0.51) |
| Sao Tome and Principe | 22 (17-30) | 36.16 (27.55-48.63) | 41 (32-53) | 38.74 (29.34-52.48) | 0.21 (0.18-0.24) |
| Saudi Arabia | 1686 (1284-2223) | 32.66 (24.17-44.12) | 6096 (4757-7921) | 36.92 (27.2-49.85) | 0.37 (0.36-0.38) |
| Senegal | 1107 (845-1455) | 37.2 (27.94-49.35) | 2744 (2111-3606) | 37.78 (28.58-50.72) | 0.06 (0.04-0.09) |
| Serbia | 5307 (3932-7011) | 45.39 (33.82-59.77) | 5688 (4597-7029) | 34.05 (28.15-41.75) | -1 (-1.25--0.74) |
| Seychelles | 32 (24-43) | 57.65 (43.89-76.07) | 64 (50-83) | 57.24 (43.64-74.83) | -0.05 (-0.07--0.04) |
| Sierra Leone | 785 (593-1049) | 40.49 (31.02-53.67) | 1403 (1091-1862) | 39.73 (30.33-53.31) | -0.11 (-0.13--0.08) |
| Singapore | 899 (704-1154) | 39.69 (30.61-52.17) | 2861 (2242-3726) | 33.07 (25.69-42.97) | -0.74 (-0.85--0.64) |
| Slovakia | 3990 (3313-4629) | 65.82 (55.05-75.92) | 7231 (5730-8496) | 73.15 (58.9-85.48) | 0.25 (0.17-0.33) |
| Slovenia | 1229 (920-1632) | 50.16 (37.53-66.24) | 2024 (1731-2316) | 48.42 (42.05-54.65) | -0.09 (-0.14--0.04) |
| Solomon Islands | 59 (45-78) | 51.19 (39.03-68.33) | 160 (124-212) | 51.92 (39.63-69.56) | 0.03 (0.02-0.04) |
| Somalia | 726 (578-945) | 34.14 (26.01-45.43) | 1951 (1522-2560) | 35.55 (27.45-47.35) | 0.14 (0.13-0.16) |
| South Africa | 9549 (7351-12620) | 49.4 (37.52-66.24) | 21323 (16354-28253) | 48.94 (37.27-64.92) | -0.06 (-0.07--0.05) |
| South Sudan | 828 (632-1090) | 35.02 (26.77-46.61) | 1351 (1062-1777) | 37.43 (28.5-49.65) | 0.24 (0.22-0.26) |
| Spain | 41675 (31727-53592) | 74.66 (57.65-95) | 64456 (56846-73040) | 69.9 (62.22-78.58) | -0.01 (-0.14-0.12) |
| Sri Lanka | 5377 (4145-7100) | 54.08 (41.22-72.82) | 14672 (11154-19567) | 55.75 (42.39-73.31) | 0.1 (0.08-0.11) |
| Sudan | 2800 (2050-3759) | 33.7 (24.56-45.03) | 6614 (4970-8739) | 37.78 (27.49-50.99) | 0.4 (0.39-0.42) |
| Suriname | 146 (114-192) | 59.89 (45.74-79.67) | 361 (277-477) | 58.05 (44.73-77.23) | -0.12 (-0.14--0.11) |
| Sweden | 13182 (9876-17727) | 85.18 (65.88-110.36) | 25710 (18631-33708) | 123.84 (92.51-159.67) | 0.83 (0.7-0.96) |
| Switzerland | 4309 (3281-5690) | 40.72 (31.61-52.93) | 6346 (5485-7579) | 36.45 (31.83-42.53) | 0.09 (-0.28-0.47) |
| Syrian Arab Republic | 1567 (1191-2037) | 34.12 (25.03-45.49) | 4228 (3163-5674) | 35.28 (25.74-47.13) | 0.1 (0.09-0.11) |
| Taiwan (Province of China) | 8121 (6340-10370) | 54.99 (43.02-71.18) | 22565 (17990-28132) | 53.21 (42.42-67.12) | -0.15 (-0.2--0.09) |
| Tajikistan | 1084 (832-1420) | 41.09 (31.09-53.75) | 2333 (1786-3072) | 40.85 (30.53-53.3) | 0.01 (-0.01-0.03) |
| Thailand | 17124 (13433-22527) | 52.67 (40.36-69.23) | 57918 (44292-77052) | 53.17 (40.48-70.77) | 0.04 (0.03-0.06) |
| Timor-Leste | 134 (106-170) | 54.47 (41.71-72.09) | 439 (332-586) | 54.61 (41.79-72.26) | 0.03 (0.02-0.05) |
| Togo | 413 (318-542) | 37.11 (28.07-49.68) | 1305 (1010-1717) | 37.53 (28.56-49.83) | 0.04 (0.02-0.05) |
| Tokelau | 1 (1-1) | 51.88 (39.68-69.51) | 1 (1-1) | 56.13 (42.64-73.71) | 0.25 (0.23-0.27) |
| Tonga | 28 (21-37) | 54.9 (41.6-73.39) | 46 (35-60) | 58.48 (44.53-76.74) | 0.21 (0.18-0.23) |
| Trinidad and Tobago | 524 (395-698) | 63.52 (49.01-84.29) | 1240 (932-1641) | 63.93 (48.81-84.75) | 0 (-0.02-0.02) |
| Tunisia | 1485 (1094-2018) | 32.93 (24.09-44.53) | 4414 (3251-5838) | 34.48 (25.02-45.65) | 0.13 (0.12-0.14) |
| Turkmenistan | 794 (597-1039) | 43.24 (32.26-56.89) | 1872 (1439-2433) | 47.04 (35.47-60.99) | 0.28 (0.27-0.29) |
| Tuvalu | 3 (2-4) | 52.18 (40.21-69.23) | 5 (4-7) | 55.34 (42.18-72.65) | 0.17 (0.15-0.19) |
| Türkiye | 10042 (7802-12979) | 32.02 (24.36-41.7) | 24774 (21786-28278) | 27.35 (24.06-31.31) | -0.78 (-0.89--0.67) |
| Uganda | 2290 (1765-2999) | 38.45 (29.55-51.14) | 5416 (4239-7160) | 38.93 (29.92-52.22) | -0.04 (-0.08-0) |
| Ukraine | 31985 (24201-42017) | 44.68 (34.28-57.82) | 35530 (26656-46895) | 46.19 (35.34-60.16) | 0.15 (0.12-0.18) |
| United Arab Emirates | 155 (120-199) | 37.02 (27.3-49.36) | 1662 (1236-2173) | 39.65 (29.19-52.8) | 0.29 (0.22-0.36) |
| United Kingdom | 48564 (36703-63566) | 53.06 (41.35-68.26) | 65549 (51519-83607) | 52.25 (41.8-65.6) | -0.27 (-0.39--0.14) |
| United Republic of Tanzania | 3448 (2650-4538) | 34.78 (26.8-46.2) | 9526 (7370-12404) | 39.1 (30.07-51.91) | 0.16 (-0.04-0.37) |
| United States of America | 245056 (180693-325021) | 75.22 (57.39-99.34) | 528208 (487191-574743) | 89.18 (82.53-96.66) | 0.54 (0.48-0.61) |
| United States Virgin Islands | 50 (38-66) | 61.81 (46.68-82.23) | 115 (83-157) | 62.03 (47.02-82.25) | 0.01 (0.01-0.02) |
| Uruguay | 1675 (1238-2253) | 42.52 (32-56.67) | 2129 (1563-2898) | 38.49 (28.98-51.8) | -0.44 (-0.49--0.4) |
| Uzbekistan | 4414 (3384-5822) | 39.64 (29.94-52.67) | 10746 (8148-14124) | 41.51 (30.94-54.26) | 0.1 (0.08-0.12) |
| Vanuatu | 32 (25-42) | 58.8 (44.26-77.86) | 93 (72-121) | 59.69 (45.68-78.54) | 0.01 (-0.02-0.03) |
| Venezuela (Bolivarian Republic of) | 6130 (4764-7939) | 65.87 (50.36-86.05) | 18248 (13775-24049) | 61.32 (46.53-81.59) | -0.24 (-0.26--0.23) |
| Viet Nam | 20315 (15605-26791) | 53.94 (41.15-71.13) | 53983 (41974-71154) | 57.26 (43.75-76.67) | 0.29 (0.24-0.34) |
| Yemen | 1282 (964-1685) | 31.04 (22.91-41.47) | 4105 (3061-5427) | 32.9 (24.21-44.14) | 0.19 (0.18-0.2) |
| Zambia | 903 (702-1185) | 34.96 (26.67-46.91) | 2320 (1828-3002) | 35.81 (27.43-47.89) | 0.08 (0.07-0.09) |
| Zimbabwe | 1472 (1118-1937) | 40.11 (30.15-53.61) | 2534 (1926-3337) | 41.28 (31.18-55.1) | 0.09 (0.08-0.11) |

ASIR=Age-standardized incidence rate, CI=Confidence interval, EAPC=Estimated annual percentage change, UI=Uncertainty interval
