# Supplementary material for: Global burden of atrial fibrillation/atrial flutter and its attributable risk factors from 1990 to 2021
Source: Europace. 2024 Jul 10;26(7):euae195. doi: 10.1093/europace/euae195 (PMC11287210; doi:10.1093/europace/euae195)
Supplement: euae195_Supplementary_Data [file euae195_supplementary_data.zip › Table S2.docx]

Table S2 The prevalence cases and age-standardized prevalence of AF/AFL in 1990 and 2021, and its temporal trends from 1990 to 2021, by 204 countries and territories

| **Characteristics** | **Number of prevalence cases in 1990** | **ASPR per 100,000**  **(95% UI)** | **Number of prevalence cases in 2021** | **ASPR per 100,000**  **(95% UI)** | **1990-2021EAPC**  **(95% CI)** |
| --- | --- | --- | --- | --- | --- |
| Country |  |  |  |  |  |
| Afghanistan | 17747 (13622-23160) | 312.82 (240.08-412.25) | 25639 (19701-33184) | 338.76 (257.55-442.8) | 0.26 (0.23-0.29) |
| Albania | 10877 (8359-13976) | 576.46 (437.91-747.53) | 26254 (20002-34169) | 582.74 (445.93-753.86) | 0.07 (0.04-0.1) |
| Algeria | 34903 (26341-46477) | 350.33 (265.16-464.73) | 116519 (89354-153163) | 376.73 (287.03-499.12) | 0.17 (0.14-0.19) |
| American Samoa | 113 (89-149) | 620 (485.43-805.63) | 289 (229-380) | 672.65 (528.47-875.08) | 0.21 (0.18-0.25) |
| Andorra | 462 (354-595) | 835.12 (645.86-1082.98) | 1172 (905-1510) | 743.99 (574.87-956.34) | -0.53 (-0.58--0.47) |
| Angola | 12292 (9545-16176) | 419.99 (324.84-550.58) | 41399 (32326-54332) | 452.08 (354.11-588.63) | 0.27 (0.26-0.28) |
| Antigua and Barbuda | 361 (277-468) | 640.52 (500.21-831.03) | 664 (518-869) | 657.2 (512.46-858.3) | 0.07 (0.05-0.08) |
| Argentina | 144409 (112881-189972) | 462.57 (362.38-606.35) | 194835 (169094-228621) | 340.72 (296.16-399.78) | -1.03 (-1.32--0.74) |
| Armenia | 12536 (9693-16435) | 507.82 (391.11-664.63) | 24452 (18788-32257) | 556.65 (424.75-729.78) | 0.33 (0.31-0.35) |
| Australia | 176034 (164773-187556) | 900.54 (846.03-956.65) | 435826 (338605-563002) | 928.31 (725.71-1201.33) | 0.19 (0.13-0.26) |
| Austria | 80258 (71857-89871) | 643.17 (578.86-714.48) | 241015 (230229-251784) | 1217.22 (1164.84-1272.17) | 2.33 (2.19-2.48) |
| Azerbaijan | 22683 (17563-29382) | 501.7 (385.38-655.28) | 48767 (38083-64570) | 540.88 (415.38-701.51) | 0.29 (0.27-0.31) |
| Bahamas | 978 (767-1269) | 691.07 (536.42-899.86) | 2533 (1987-3335) | 674.57 (529.29-883.91) | -0.06 (-0.08--0.05) |
| Bahrain | 462 (356-611) | 361.4 (274.6-473.49) | 2667 (2055-3468) | 386.71 (293.82-505.55) | 0.18 (0.16-0.21) |
| Bangladesh | 190657 (146465-248663) | 472.02 (362.32-621.61) | 623060 (480696-812961) | 494.7 (380.67-643.93) | 0.17 (0.11-0.23) |
| Barbados | 2085 (1592-2746) | 666.99 (518.95-866.63) | 3693 (2829-4833) | 701.8 (540.68-913.61) | 0.17 (0.15-0.19) |
| Belarus | 71527 (55047-92982) | 556.58 (429.87-719.16) | 101036 (78621-132534) | 620.57 (486.13-809.95) | 0.34 (0.31-0.38) |
| Belgium | 112962 (86002-147149) | 704.67 (544.2-918.24) | 163236 (131345-207711) | 675.51 (553.96-847.21) | -0.22 (-0.3--0.15) |
| Belize | 607 (476-781) | 668.4 (523.01-861.13) | 1881 (1485-2467) | 693.37 (538.53-897.5) | 0.14 (0.12-0.16) |
| Benin | 6795 (5253-8934) | 377.41 (290.58-493.99) | 17562 (13735-23147) | 396.48 (308.75-520.98) | 0.16 (0.15-0.17) |
| Bermuda | 423 (328-552) | 705.31 (550.87-914.8) | 1007 (782-1312) | 700.19 (547.46-909.57) | -0.04 (-0.06--0.03) |
| Bhutan | 864 (668-1134) | 467.22 (356.2-612.99) | 2770 (2170-3630) | 492.91 (384.1-647.49) | 0.18 (0.15-0.2) |
| Bolivia (Plurinational State of) | 16730 (13176-22025) | 595.3 (464.6-774.97) | 51917 (40535-67894) | 622.3 (488.06-809.65) | 0.18 (0.16-0.19) |
| Bosnia and Herzegovina | 20194 (15626-26610) | 551.75 (424.34-722.2) | 36920 (28206-47883) | 574.34 (442.67-741.06) | 0.17 (0.14-0.2) |
| Botswana | 1971 (1525-2589) | 435.97 (337.45-570.77) | 5721 (4459-7586) | 467.19 (359.37-610.96) | 0.21 (0.21-0.22) |
| Brazil | 644967 (507649-839852) | 802.83 (631.35-1041.09) | 1961680 (1546310-2544332) | 795.32 (626.23-1025.71) | -0.07 (-0.09--0.06) |
| Brunei Darussalam | 525 (412-677) | 537.28 (419.06-696.7) | 1479 (1155-1910) | 452.03 (354.84-582.96) | -0.66 (-0.71--0.61) |
| Bulgaria | 70573 (53726-92308) | 580.26 (444.98-749.7) | 86405 (64937-113573) | 564.76 (430.67-735.55) | -0.01 (-0.05-0.03) |
| Burkina Faso | 13882 (10757-18200) | 377.76 (293.77-494.41) | 31626 (24731-41910) | 397.21 (311.38-519) | 0.14 (0.13-0.16) |
| Burundi | 8282 (6487-10976) | 407.99 (320.53-536.99) | 17035 (13278-22301) | 418.87 (326.88-552.33) | 0.1 (0.09-0.11) |
| Cabo Verde | 943 (728-1248) | 397.4 (310.11-520.15) | 1754 (1380-2302) | 419.74 (323.1-551.73) | 0.19 (0.18-0.2) |
| Cambodia | 20822 (16350-27382) | 562.64 (441.69-725.52) | 61483 (48621-81182) | 578.56 (457.17-751.32) | 0.08 (0.06-0.09) |
| Cameroon | 14149 (10972-18719) | 381.73 (293.64-497.91) | 45126 (35031-58573) | 432.9 (335.41-563.68) | 0.38 (0.31-0.45) |
| Canada | 383916 (305778-474738) | 1163.51 (929.48-1432.68) | 731819 (554975-954038) | 958.83 (733.17-1244.27) | -0.53 (-0.65--0.42) |
| Central African Republic | 3545 (2742-4677) | 416.27 (320.82-545.51) | 6761 (5202-8922) | 416.26 (325.14-546.14) | -0.01 (-0.01-0) |
| Chad | 9335 (7171-12424) | 366 (283.48-480.61) | 18475 (14434-24478) | 383.73 (298.18-504.47) | 0.16 (0.16-0.17) |
| Chile | 48779 (37923-63534) | 517.99 (401.73-671.12) | 127594 (98729-165412) | 489.14 (379.61-632.16) | -0.32 (-0.37--0.27) |
| China | 3195309 (2518983-4168290) | 457.72 (358.93-594.96) | 10775721 (8531627-14014036) | 524 (418.15-681.23) | 0.48 (0.38-0.58) |
| Colombia | 101960 (80303-132427) | 648.41 (506.48-845.13) | 361800 (282565-475575) | 650.75 (507.97-853.91) | 0.04 (0.02-0.05) |
| Comoros | 655 (509-868) | 395.77 (309.16-515.75) | 1811 (1415-2366) | 415.57 (326.17-548.36) | 0.15 (0.14-0.16) |
| Congo | 3685 (2852-4857) | 433.69 (337.43-567.64) | 9845 (7730-12833) | 462.37 (360.8-600.06) | 0.22 (0.2-0.23) |
| Cook Islands | 65 (51-87) | 595.69 (462.18-769.16) | 178 (138-232) | 683.51 (533.18-886.86) | 0.44 (0.4-0.48) |
| Costa Rica | 12283 (9603-15941) | 740.27 (578.22-963.56) | 39959 (31002-51910) | 727.25 (561.47-946.4) | -0.07 (-0.08--0.07) |
| Croatia | 22653 (19259-27369) | 388.27 (328.33-464.05) | 39620 (35576-44450) | 420.02 (381.35-470.13) | 0.69 (0.45-0.93) |
| Cuba | 69471 (53452-90480) | 684.27 (530.29-890.3) | 136060 (106604-175458) | 671.42 (527.93-869.79) | -0.06 (-0.08--0.04) |
| Cyprus | 5114 (3908-6514) | 652.09 (507.78-834.3) | 10427 (8434-13170) | 487.17 (395.53-615.62) | -0.83 (-0.98--0.69) |
| Czechia | 93403 (71391-120323) | 665.74 (509.98-854.56) | 228258 (188006-254737) | 981.52 (815.26-1089.62) | 1.44 (1.06-1.81) |
| Côte d'Ivoire | 12409 (9635-16308) | 402.26 (310.63-527.53) | 38549 (30111-50565) | 424.45 (328.78-553.75) | 0.16 (0.15-0.17) |
| Democratic People's Republic of Korea | 73282 (57655-93865) | 534.03 (422.64-695.54) | 166218 (132256-214516) | 532.4 (422.66-687.42) | -0.03 (-0.05--0.02) |
| Democratic Republic of the Congo | 51338 (39752-66862) | 429.1 (331.65-559.18) | 118526 (92188-158139) | 411.45 (320.33-546.46) | -0.18 (-0.2--0.17) |
| Denmark | 69659 (53710-89658) | 817.12 (637.85-1043.61) | 111718 (88772-138463) | 908.22 (736.43-1119.51) | -0.06 (-0.24-0.13) |
| Djibouti | 416 (324-545) | 388.29 (306.04-508.88) | 2213 (1710-2891) | 424.62 (335.81-555.29) | 0.31 (0.3-0.32) |
| Dominica | 389 (299-510) | 650.97 (507.12-849.2) | 544 (418-711) | 668.86 (514.54-861.99) | 0.09 (0.08-0.11) |
| Dominican Republic | 22177 (17125-29173) | 662.46 (509.25-862.76) | 64889 (50825-84248) | 667.32 (520.85-869.63) | 0 (-0.01-0.01) |
| Ecuador | 29616 (23224-38669) | 606.55 (471.16-785.92) | 98459 (77061-128281) | 615.7 (480.28-800.86) | 0.08 (0.07-0.1) |
| Egypt | 67055 (51335-87638) | 333.76 (255.54-434.8) | 184697 (143378-239672) | 393 (297.72-514.22) | 0.58 (0.55-0.6) |
| El Salvador | 18470 (14488-24034) | 654.08 (510.09-850.26) | 42585 (33215-55380) | 665.9 (520.53-865.31) | 0.07 (0.06-0.07) |
| Equatorial Guinea | 634 (492-835) | 413.19 (321.21-541.73) | 1997 (1582-2592) | 487.69 (375.94-633.88) | 0.58 (0.56-0.61) |
| Eritrea | 3091 (2340-4067) | 371.42 (292.85-487.09) | 8704 (6755-11290) | 391.78 (304.41-516.89) | 0.17 (0.16-0.18) |
| Estonia | 11240 (8653-14638) | 544.37 (421.2-706.07) | 17269 (13159-22327) | 619.55 (480.95-800.04) | 0.48 (0.44-0.53) |
| Eswatini | 1042 (813-1372) | 445.49 (345.52-584.17) | 2108 (1632-2790) | 468.81 (364.74-615.22) | 0.16 (0.15-0.17) |
| Ethiopia | 63428 (49352-84190) | 406.05 (319.38-532.83) | 184880 (146237-239761) | 479.99 (377.48-630.3) | 0.68 (0.6-0.77) |
| Fiji | 1737 (1367-2298) | 607.48 (474.79-789.89) | 4434 (3483-5800) | 676.96 (527.93-884.27) | 0.37 (0.35-0.39) |
| Finland | 85278 (64676-106114) | 1168.45 (891.81-1441.77) | 117234 (94965-141038) | 862.54 (725.46-1013.76) | -1.12 (-1.23--1.01) |
| France | 703229 (534466-921039) | 803.34 (620.29-1041.29) | 1081926 (820574-1416350) | 717.98 (554.42-925.96) | -0.5 (-0.57--0.44) |
| Gabon | 2194 (1700-2905) | 432.99 (334.06-567.18) | 4065 (3180-5323) | 465.31 (360.35-610.14) | 0.22 (0.22-0.23) |
| Gambia | 1153 (900-1502) | 394.31 (307.81-516.75) | 3491 (2752-4643) | 411.42 (317.56-536.48) | 0.11 (0.08-0.14) |
| Georgia | 33800 (25849-44479) | 558.89 (425.91-728.4) | 35412 (26991-46236) | 568.73 (436.23-738.62) | 0 (-0.02-0.03) |
| Germany | 1225849 (937079-1593976) | 927.92 (719.81-1195.45) | 2155152 (1896612-2379617) | 1072.86 (943.99-1172.82) | 0.38 (0.16-0.6) |
| Ghana | 20084 (15703-26388) | 392.93 (305.98-515.13) | 60669 (47244-80034) | 432.42 (334.33-567.15) | 0.23 (0.17-0.3) |
| Greece | 105797 (87036-133969) | 686.94 (566.89-865.16) | 175318 (132665-230098) | 662.08 (510.11-855.56) | -0.32 (-0.58--0.06) |
| Greenland | 292 (228-377) | 1087.86 (839.27-1403.54) | 617 (478-795) | 1014.83 (775.76-1305.09) | -0.15 (-0.23--0.07) |
| Grenada | 498 (382-652) | 642.5 (501.35-833.65) | 695 (538-916) | 653.72 (506.53-851.89) | 0 (-0.04-0.05) |
| Guam | 369 (289-473) | 597.15 (469.33-773.68) | 1453 (1138-1871) | 672.25 (525.78-866.79) | 0.36 (0.32-0.41) |
| Guatemala | 17778 (13983-23674) | 604.89 (470-790.52) | 66296 (51813-86475) | 636.34 (499.2-825.44) | 0.24 (0.21-0.28) |
| Guinea | 11006 (8485-14476) | 370.46 (286.63-484.66) | 19024 (14744-25081) | 387.59 (300.45-506.19) | 0.13 (0.13-0.14) |
| Guinea-Bissau | 1218 (950-1606) | 379.44 (293.67-497.66) | 2225 (1727-2948) | 394.7 (304.39-521.63) | 0.12 (0.11-0.12) |
| Guyana | 2254 (1769-2967) | 671.43 (519.76-877.4) | 3827 (2984-5099) | 667.24 (519.54-874.3) | -0.01 (-0.03-0.01) |
| Haiti | 17161 (13462-22578) | 637.48 (497.01-823.22) | 39243 (30771-51649) | 654.08 (507-860.17) | 0.11 (0.1-0.12) |
| Honduras | 12036 (9402-15784) | 659.29 (513.99-858.55) | 37962 (29758-49433) | 664.18 (516.7-857.85) | 0.02 (0.01-0.03) |
| Hungary | 101902 (76999-132894) | 681.32 (519.83-880.64) | 118850 (90414-156204) | 574.65 (443.45-748.72) | -0.6 (-0.66--0.55) |
| Iceland | 2087 (1581-2704) | 704.04 (536.96-912.64) | 4788 (3892-5821) | 793.27 (651.04-960.17) | 0.22 (0.14-0.3) |
| India | 1860594 (1442729-2458704) | 521.35 (402.59-685.09) | 5612366 (4314558-7360330) | 533.92 (412.71-700) | 0.09 (0.08-0.11) |
| Indonesia | 560360 (442858-730937) | 700.63 (547.24-905.66) | 1474160 (1152713-1922564) | 728.2 (569.74-942.29) | 0.15 (0.14-0.16) |
| Iran (Islamic Republic of) | 75833 (58814-99095) | 385.97 (296.24-507.56) | 294249 (230024-382166) | 425.39 (327.17-559.23) | 0.23 (0.18-0.28) |
| Iraq | 26906 (20773-34733) | 373.36 (285.05-485.8) | 78701 (61203-102060) | 406.12 (308.47-532.03) | 0.26 (0.24-0.28) |
| Ireland | 31562 (23747-40777) | 757.71 (578.57-979.99) | 54616 (42201-70424) | 666.48 (516.95-858.36) | -0.86 (-0.98--0.73) |
| Israel | 41616 (31818-54187) | 848.35 (656.52-1096.61) | 147352 (122034-167473) | 1155.51 (958.33-1312.39) | 1.46 (1.15-1.77) |
| Italy | 824078 (633970-1072624) | 897.86 (697.07-1163.46) | 1327156 (1001901-1743600) | 821.57 (632.6-1065.6) | -0.6 (-0.69--0.51) |
| Jamaica | 11880 (9135-15489) | 647.52 (501.68-840.64) | 21347 (16750-27574) | 675.56 (527.03-877.87) | 0.15 (0.11-0.18) |
| Japan | 873570 (685978-1135914) | 519.91 (411.63-671.77) | 1498702 (1171946-1964169) | 391.87 (310.63-511.22) | -0.89 (-1.16--0.61) |
| Jordan | 3649 (2816-4743) | 354.23 (268.4-466.12) | 23983 (18451-31112) | 396.61 (300.63-520.3) | 0.35 (0.34-0.37) |
| Kazakhstan | 65048 (50303-84806) | 562.81 (428.43-731.6) | 96397 (74221-126067) | 589.25 (453.42-768.59) | 0.16 (0.13-0.18) |
| Kenya | 32479 (25576-42395) | 455.99 (357.55-595.62) | 90501 (71144-118517) | 467.05 (366.94-610.02) | 0.07 (0.06-0.08) |
| Kiribati | 170 (133-221) | 577.26 (450.63-748.84) | 347 (272-457) | 598.52 (466.2-771.38) | 0.09 (0.07-0.11) |
| Kuwait | 1721 (1337-2199) | 369.85 (282.78-483.44) | 9836 (7697-12638) | 408.07 (310.58-530.22) | 0.29 (0.24-0.35) |
| Kyrgyzstan | 13329 (10299-17484) | 478.91 (366.47-626.57) | 20779 (16256-27052) | 491.16 (377.25-639.22) | 0.05 (0.04-0.06) |
| Lao People's Democratic Republic | 9988 (7851-13152) | 587.21 (457.7-766.12) | 23869 (18913-31406) | 605.11 (475.86-782.28) | 0.1 (0.08-0.12) |
| Latvia | 18682 (14270-24444) | 516.84 (396.14-673.25) | 25774 (22299-29649) | 629.57 (547.41-715.02) | 0.97 (0.8-1.14) |
| Lebanon | 6336 (4939-8255) | 342.25 (261.21-444.6) | 24591 (18725-32516) | 387.14 (298.49-511.08) | 0.44 (0.42-0.45) |
| Lesotho | 3040 (2351-3994) | 403.46 (311.48-530.49) | 3809 (2967-5053) | 423.73 (327.59-555.16) | 0.14 (0.13-0.14) |
| Liberia | 4038 (3146-5396) | 397.36 (308.62-524.77) | 7117 (5638-9316) | 408.89 (317.19-535.39) | 0.1 (0.09-0.11) |
| Libya | 6061 (4645-7875) | 367.31 (276.5-479.71) | 17554 (13696-22623) | 398.27 (304.76-518.53) | 0.24 (0.21-0.26) |
| Lithuania | 25740 (19772-33473) | 567.12 (437.53-735.84) | 37996 (28984-48993) | 634.2 (495.02-808.33) | 0.42 (0.38-0.46) |
| Luxembourg | 4288 (3610-5095) | 763.59 (647.3-906.09) | 8302 (7313-9305) | 751.62 (663.73-841.08) | 0.01 (-0.04-0.05) |
| Madagascar | 16984 (13349-22344) | 391.91 (308.5-516.98) | 37729 (29479-49128) | 417.24 (327.3-548.36) | 0.22 (0.21-0.23) |
| Malawi | 13014 (10145-17325) | 406.12 (318.51-530.77) | 27955 (21964-36667) | 445.44 (350.05-586.14) | 0.29 (0.27-0.32) |
| Malaysia | 52682 (42117-68276) | 631.34 (495.34-821.09) | 174176 (137798-228296) | 673.16 (526.92-873.05) | 0.34 (0.29-0.38) |
| Maldives | 424 (329-553) | 601.49 (471.53-786.4) | 1871 (1497-2409) | 619.06 (486.08-803.07) | 0.05 (0.04-0.07) |
| Mali | 11800 (9185-15919) | 363.12 (281.72-476.4) | 28145 (22007-37160) | 384.98 (298.11-507.1) | 0.18 (0.17-0.19) |
| Malta | 2836 (2179-3655) | 676.78 (521.13-871.7) | 6323 (5524-7076) | 598.49 (529.84-673.49) | -0.06 (-0.35-0.23) |
| Marshall Islands | 73 (57-93) | 543.46 (423.67-699.52) | 157 (122-206) | 578.23 (451.61-751.27) | 0.18 (0.17-0.19) |
| Mauritania | 3395 (2607-4514) | 383.24 (295.57-505.2) | 8034 (6250-10647) | 419.11 (324.19-548.09) | 0.28 (0.27-0.3) |
| Mauritius | 3919 (3071-5108) | 621.51 (488.13-813.67) | 11425 (8978-15014) | 642.42 (500.8-837.33) | -0.02 (-0.08-0.03) |
| Mexico | 278511 (219192-361872) | 732.26 (571.33-951.32) | 899391 (705327-1167818) | 746.46 (582.69-967.96) | 0.12 (0.1-0.14) |
| Micronesia (Federated States of) | 242 (191-315) | 574.85 (450.09-745.62) | 353 (275-461) | 586.69 (458.47-759.65) | 0.05 (0.04-0.06) |
| Monaco | 602 (451-792) | 787.04 (609.64-1021.06) | 754 (573-971) | 718.17 (557.76-916.81) | -0.41 (-0.46--0.35) |
| Mongolia | 4854 (3718-6266) | 507.69 (387.87-661.82) | 10601 (8189-13897) | 544.58 (413.48-704.44) | 0.25 (0.23-0.27) |
| Montenegro | 3723 (2851-4875) | 622.42 (474.22-809.12) | 5917 (4506-7731) | 597.77 (457.54-773.59) | -0.08 (-0.12--0.05) |
| Morocco | 45335 (34423-58980) | 360.21 (273.2-470.75) | 117079 (89916-151800) | 383.34 (292.51-505.95) | 0.12 (0.09-0.15) |
| Mozambique | 20053 (15801-26456) | 397.84 (310.59-525.54) | 39677 (30931-51541) | 427.96 (333.79-561.38) | 0.2 (0.19-0.22) |
| Myanmar | 118341 (93009-156133) | 613.93 (479.1-797.7) | 267795 (211909-352206) | 614.44 (480.43-795.77) | 0.01 (0-0.02) |
| Namibia | 2284 (1755-2994) | 432.25 (335.34-566.54) | 5143 (3995-6875) | 443.93 (340.35-581.29) | 0.05 (0.04-0.06) |
| Nauru | 23 (18-30) | 642.55 (498.21-838.02) | 31 (25-40) | 671.41 (528.4-873.97) | 0.06 (0.04-0.09) |
| Nepal | 34654 (26729-45519) | 471.77 (362.87-619.63) | 96213 (73203-125250) | 471.74 (359.9-618.89) | 0.02 (-0.05-0.09) |
| Netherlands | 172361 (156082-189738) | 834.04 (753-922.78) | 287028 (243038-332612) | 769.98 (654.22-894.08) | -0.59 (-0.72--0.46) |
| New Zealand | 32004 (25316-40250) | 806.03 (643.22-1015.05) | 72708 (60818-88247) | 835.21 (704.87-1004.71) | 0.2 (0.1-0.29) |
| Nicaragua | 9056 (7066-11755) | 657.62 (505.51-861.44) | 30250 (23880-39026) | 661.52 (516.78-851.32) | 0.03 (0.02-0.05) |
| Niger | 8171 (6367-10826) | 368.55 (285.62-485.65) | 25954 (20174-34414) | 383.57 (295.92-504.59) | 0.13 (0.11-0.15) |
| Nigeria | 153286 (119379-203690) | 399.72 (310.99-523.95) | 353475 (278100-466330) | 461.68 (358.97-602.32) | 0.57 (0.52-0.62) |
| Niue | 14 (11-19) | 616.15 (483.25-798.37) | 14 (11-19) | 677.63 (531.4-878.21) | 0.27 (0.23-0.31) |
| North Macedonia | 10256 (7924-13365) | 593.55 (455.11-776.64) | 18793 (14283-24572) | 572.89 (435.57-741.4) | -0.08 (-0.11--0.05) |
| Northern Mariana Islands | 82 (64-104) | 633.21 (496.4-821.86) | 294 (229-378) | 662.43 (515.37-853.03) | 0.1 (0.06-0.13) |
| Norway | 57867 (44003-75498) | 798.36 (620.77-1032.65) | 79412 (61345-102809) | 761.48 (589.35-985.49) | -0.16 (-0.21--0.12) |
| Oman | 1615 (1248-2101) | 292.89 (225.07-380.76) | 5827 (4557-7637) | 365.26 (276.39-482.95) | 0.84 (0.77-0.91) |
| Pakistan | 270729 (206528-355785) | 554.73 (429.37-728.7) | 548174 (427141-726637) | 558.61 (429.5-735.69) | 0.06 (0.04-0.07) |
| Palau | 49 (39-65) | 585.01 (458.06-756.17) | 125 (98-163) | 646.55 (506.74-842.07) | 0.29 (0.26-0.32) |
| Palestine | 2437 (1859-3179) | 323.2 (244.98-422.73) | 7197 (5525-9405) | 347.45 (265.23-453.98) | 0.18 (0.16-0.2) |
| Panama | 9494 (7472-12317) | 676.55 (528.06-881.82) | 30751 (24204-39735) | 691.05 (542.43-895.33) | 0.1 (0.08-0.12) |
| Papua New Guinea | 7335 (5716-9600) | 532.9 (418.45-697.52) | 21815 (17006-28137) | 543.71 (426.86-705.94) | 0.05 (0.04-0.07) |
| Paraguay | 16763 (13141-21472) | 797.07 (624.59-1027.49) | 43040 (33767-55877) | 769.35 (602.53-992.61) | -0.14 (-0.17--0.11) |
| Peru | 66121 (52636-86420) | 597.59 (472.59-772.26) | 220883 (171496-283755) | 668.37 (516.03-860.23) | 0.49 (0.43-0.54) |
| Philippines | 160947 (126928-211314) | 648.69 (510.61-837.57) | 466916 (369507-610795) | 653.73 (513.13-843.12) | -0.01 (-0.02-0) |
| Poland | 276686 (211770-358761) | 640.38 (488.94-828.18) | 648195 (491254-846914) | 866.31 (664.47-1117.94) | 0.25 (-0.03-0.53) |
| Portugal | 105934 (80401-138675) | 758.21 (585.05-989.75) | 192005 (161459-229225) | 695.71 (591.04-820.14) | -0.27 (-0.31--0.23) |
| Puerto Rico | 25706 (19933-33640) | 709.64 (552.95-924.66) | 56494 (43078-73735) | 715.09 (552.46-927.13) | 0.02 (0.01-0.04) |
| Qatar | 298 (228-389) | 363.49 (278.22-475.53) | 3005 (2251-3953) | 402.59 (305.76-529.29) | 0.26 (0.22-0.3) |
| Republic of Korea | 167647 (132028-217480) | 610.53 (486.7-785.88) | 628474 (544533-739494) | 675.14 (584.7-790.59) | 0.52 (0.36-0.68) |
| Republic of Moldova | 23013 (17873-30099) | 551.37 (427.29-709.11) | 37430 (28839-48105) | 622.47 (480.68-796.46) | 0.45 (0.43-0.46) |
| Romania | 153776 (118560-200226) | 568.65 (435.33-734.23) | 162826 (141283-191969) | 412.59 (359.76-488) | -1.17 (-1.44--0.9) |
| Russian Federation | 1053310 (820702-1363643) | 599.94 (467.24-774.28) | 1639057 (1272601-2138452) | 673.56 (526.09-872.26) | 0.43 (0.33-0.52) |
| Rwanda | 9685 (7590-12457) | 414.73 (326-538.65) | 23218 (18165-30751) | 431.09 (337.04-563.82) | 0.11 (0.1-0.11) |
| Saint Kitts and Nevis | 253 (194-329) | 653.45 (516.25-841.33) | 406 (319-530) | 657.86 (511.85-859.19) | -0.02 (-0.04-0.01) |
| Saint Lucia | 583 (447-760) | 688.42 (532.55-895.22) | 1618 (1260-2099) | 680.63 (528.46-880.89) | 0.01 (-0.01-0.03) |
| Saint Vincent and the Grenadines | 443 (341-581) | 631.81 (489.77-823.79) | 939 (732-1231) | 670.59 (523.25-876.28) | 0.18 (0.17-0.19) |
| Samoa | 451 (356-591) | 612.88 (479.08-796.86) | 825 (649-1086) | 637.38 (497.38-825.04) | 0.07 (0.05-0.1) |
| San Marino | 293 (225-382) | 800.69 (623.05-1036.98) | 583 (442-761) | 720.08 (555.9-936.44) | -0.44 (-0.48--0.4) |
| Sao Tome and Principe | 234 (182-309) | 391.01 (302-510.48) | 413 (324-548) | 431.62 (332.48-560.79) | 0.3 (0.26-0.33) |
| Saudi Arabia | 15737 (12130-20450) | 330.72 (250.33-435.24) | 54167 (42125-71479) | 391.86 (301.89-511.74) | 0.53 (0.51-0.54) |
| Senegal | 11310 (8799-15063) | 404.14 (314.98-526.95) | 28239 (22128-37630) | 416.56 (324.04-545.14) | 0.1 (0.07-0.13) |
| Serbia | 56918 (44033-74679) | 549.73 (422.16-715.09) | 70792 (59259-87626) | 409.9 (345.15-506.86) | -1.01 (-1.31--0.72) |
| Seychelles | 354 (278-458) | 630.72 (495.41-812.41) | 696 (551-905) | 644.8 (506.5-834.04) | 0.04 (0.02-0.06) |
| Sierra Leone | 8193 (6376-10749) | 441.68 (342.89-578.08) | 14273 (11176-18823) | 438.39 (341.01-578.66) | -0.08 (-0.12--0.05) |
| Singapore | 10361 (8190-13361) | 496.12 (394.24-643.05) | 36795 (28982-47620) | 430.26 (339.42-557.39) | -0.62 (-0.72--0.51) |
| Slovakia | 50960 (42632-58858) | 848.64 (713.21-976.42) | 90634 (74283-106522) | 917.33 (758.01-1072.43) | 0.16 (0.08-0.24) |
| Slovenia | 15036 (11602-19562) | 610.78 (474.02-792.53) | 28595 (25053-32065) | 617.35 (543.74-691.9) | 0.05 (0.01-0.09) |
| Solomon Islands | 583 (457-752) | 551.7 (429.29-719.58) | 1594 (1258-2087) | 568.32 (443.6-747.82) | 0.06 (0.05-0.07) |
| Somalia | 6910 (5439-8954) | 374.38 (294.47-490.01) | 18687 (14347-24314) | 392.02 (308.37-516.94) | 0.15 (0.14-0.16) |
| South Africa | 98210 (77197-127867) | 532.23 (414.73-693.35) | 217357 (169171-286785) | 526.19 (408.32-689.91) | -0.08 (-0.09--0.06) |
| South Sudan | 8739 (6834-11524) | 388.78 (304.99-513.39) | 13605 (10696-17831) | 423.26 (332.01-554.83) | 0.3 (0.28-0.32) |
| Spain | 489404 (384802-625445) | 876.31 (690.91-1108.62) | 902888 (806291-1004786) | 863.92 (780.25-960.23) | 0.05 (-0.09-0.19) |
| Sri Lanka | 55986 (44216-73510) | 600.31 (472.18-777.41) | 163229 (128662-213322) | 632.17 (494.65-817.05) | 0.17 (0.15-0.19) |
| Sudan | 26219 (19909-34245) | 336.41 (256.33-438.22) | 63916 (49242-83110) | 394.5 (298.41-515.66) | 0.55 (0.54-0.57) |
| Suriname | 1552 (1216-2030) | 662.16 (514.71-863.35) | 3954 (3098-5171) | 651.22 (508.16-840.34) | -0.08 (-0.09--0.07) |
| Sweden | 163917 (128606-209330) | 1010.87 (810.08-1286.9) | 360875 (270489-465091) | 1529.82 (1166.57-1943.26) | 1.05 (0.94-1.17) |
| Switzerland | 51170 (39839-65184) | 463.76 (360.67-586.53) | 84810 (74812-97781) | 439.01 (388.91-505.6) | 0.3 (-0.1-0.7) |
| Syrian Arab Republic | 15095 (11605-19661) | 348.36 (264.02-453.87) | 41606 (32220-54570) | 377.81 (288.12-497.32) | 0.24 (0.23-0.26) |
| Taiwan (Province of China) | 90993 (73354-115877) | 645.3 (520.86-815.93) | 273226 (222314-341516) | 629.83 (513.33-784.23) | -0.18 (-0.23--0.13) |
| Tajikistan | 11955 (9331-15431) | 476.78 (369.22-619.07) | 23850 (18509-31221) | 489.48 (373.73-641.48) | 0.13 (0.1-0.15) |
| Thailand | 176405 (139408-229043) | 577.24 (454.35-745.36) | 661374 (516881-864805) | 603.8 (470.51-784.54) | 0.16 (0.14-0.18) |
| Timor-Leste | 1280 (1023-1638) | 585.93 (462.52-761.76) | 4680 (3685-6092) | 605.83 (476.38-787.01) | 0.14 (0.13-0.15) |
| Togo | 4094 (3207-5424) | 403.31 (311.69-529.91) | 12756 (9931-16740) | 411.66 (322.28-540.75) | 0.05 (0.04-0.07) |
| Tokelau | 7 (6-9) | 559.76 (436.38-722.71) | 9 (7-12) | 629.56 (494.07-812.57) | 0.36 (0.34-0.39) |
| Tonga | 292 (231-381) | 600.15 (470.99-773.65) | 507 (400-655) | 654.77 (516.14-842.08) | 0.27 (0.24-0.29) |
| Trinidad and Tobago | 5672 (4370-7442) | 710.06 (551.1-922.55) | 14037 (10919-18399) | 732.58 (567.98-954.23) | 0.08 (0.06-0.1) |
| Tunisia | 14175 (10853-18659) | 335.66 (257.82-444.15) | 45069 (34412-58372) | 365.01 (276.78-471.64) | 0.24 (0.23-0.26) |
| Turkmenistan | 8444 (6460-11054) | 501.72 (380.44-654.49) | 19896 (15619-25583) | 563.75 (432.79-729.21) | 0.4 (0.39-0.41) |
| Tuvalu | 30 (24-40) | 548.68 (433.76-708.17) | 57 (45-75) | 609.07 (478.07-796.75) | 0.31 (0.28-0.33) |
| Türkiye | 99437 (77991-127594) | 332.71 (262.14-425.52) | 247837 (221168-275262) | 282.89 (252.21-314.68) | -0.84 (-1--0.69) |
| Uganda | 24063 (18872-31962) | 431.96 (338.73-567.67) | 55891 (43879-73062) | 442.48 (347.59-586.14) | 0 (-0.04-0.04) |
| Ukraine | 397837 (308635-521139) | 559.63 (438.88-727.55) | 468750 (362555-614415) | 586.74 (457.73-760.23) | 0.19 (0.16-0.23) |
| United Arab Emirates | 1294 (990-1674) | 381.72 (289.37-494.96) | 13475 (9821-18266) | 424.69 (326.48-554.82) | 0.41 (0.33-0.49) |
| United Kingdom | 589601 (466035-750521) | 620.1 (495.41-784.31) | 888645 (725229-1093517) | 648.32 (535.59-793.81) | -0.07 (-0.2-0.05) |
| United Republic of Tanzania | 36073 (28046-47724) | 387.72 (303.94-510.65) | 99864 (78447-132449) | 445.22 (349.86-586.08) | 0.26 (0.07-0.45) |
| United States of America | 2909341 (2226181-3751437) | 875.02 (675.05-1125.91) | 6373868 (5938751-6852944) | 1040.36 (973.52-1116.25) | 0.57 (0.5-0.64) |
| United States Virgin Islands | 525 (409-695) | 695 (537.89-902.89) | 1367 (1038-1806) | 710.98 (550.75-926.06) | 0.07 (0.06-0.07) |
| Uruguay | 18817 (14625-24726) | 474.49 (372.7-619.64) | 26364 (20167-34763) | 445.88 (345.48-579.21) | -0.32 (-0.36--0.27) |
| Uzbekistan | 49809 (38683-65011) | 460.42 (354.54-596.53) | 112675 (87824-148398) | 496.56 (380.6-651.61) | 0.21 (0.2-0.23) |
| Vanuatu | 318 (247-418) | 643.66 (500.24-835.55) | 942 (739-1229) | 658.57 (516.53-852.91) | 0.02 (-0.01-0.05) |
| Venezuela (Bolivarian Republic of) | 65247 (51221-84287) | 743.12 (578.4-960.3) | 202304 (158201-263377) | 700.83 (544.82-906.43) | -0.2 (-0.21--0.19) |
| Viet Nam | 214245 (169209-280476) | 581.97 (454.92-753.15) | 581496 (457784-771131) | 644.9 (502.66-844.07) | 0.43 (0.38-0.48) |
| Yemen | 11693 (8974-15102) | 307.38 (234.12-401.08) | 38432 (29592-49671) | 337.23 (255.75-443.55) | 0.32 (0.31-0.33) |
| Zambia | 9243 (7217-12053) | 387.55 (304.25-508.42) | 23356 (18374-30162) | 404.89 (320.47-528.63) | 0.14 (0.13-0.16) |
| Zimbabwe | 14595 (11343-19291) | 430.38 (334.6-559.29) | 24452 (18907-32514) | 444.98 (344.65-580.89) | 0.08 (0.07-0.1) |

ASPR=Age-standardized prevalence rate, CI=Confidence interval, EAPC=Estimated annual percentage change, UI=Uncertainty interval
