# Supplementary material for: Global burden of atrial fibrillation/atrial flutter and its attributable risk factors from 1990 to 2021
Source: Europace. 2024 Jul 10;26(7):euae195. doi: 10.1093/europace/euae195 (PMC11287210; doi:10.1093/europace/euae195)
Supplement: euae195_Supplementary_Data [file euae195_supplementary_data.zip › Table S3 .docx]

Table S3 The deaths cases and age-standardized death of AF/AFL in 1990 and 2021, and its temporal trends from 1990 to 2021, by 204 countries and territories

| **Characteristics** | **Number of deaths cases in 1990** | **ASDR per 100,000**  **(95% UI)** | **Number of deaths cases in 2021** | **ASDR per 100,000**  **(95% UI)** | **1990-2021EAPC**  **(95% CI)** |
| --- | --- | --- | --- | --- | --- |
| Country |  |  |  |  |  |
| Afghanistan | 113 (59-190) | 2.75 (1.46-4.55) | 179 (111-264) | 3.28 (2.04-4.86) | 0.66 (0.63-0.7) |
| Albania | 59 (51-69) | 4.02 (3.41-4.65) | 181 (132-229) | 4.72 (3.41-6.04) | 0.92 (0.73-1.1) |
| Algeria | 244 (173-322) | 5.32 (3.83-6.82) | 1114 (824-1390) | 5.97 (4.5-7.24) | 1.15 (0.88-1.42) |
| American Samoa | 1 (1-1) | 6.07 (4.86-7.79) | 2 (1-3) | 6.46 (4.61-8.54) | 0.38 (0.33-0.44) |
| Andorra | 2 (1-3) | 4.8 (3.41-6.92) | 7 (5-9) | 3.66 (2.64-4.82) | -0.59 (-0.77--0.4) |
| Angola | 74 (48-108) | 3.85 (2.52-5.65) | 279 (194-392) | 4.86 (3.35-6.83) | 0.64 (0.55-0.73) |
| Antigua and Barbuda | 4 (4-5) | 7 (6.2-7.77) | 5 (5-6) | 6.61 (5.97-7.14) | -0.06 (-0.28-0.17) |
| Argentina | 946 (851-1019) | 3.75 (3.32-4.06) | 2027 (1752-2197) | 3.42 (2.95-3.7) | 0.49 (0.15-0.83) |
| Armenia | 31 (27-35) | 1.52 (1.31-1.77) | 108 (93-122) | 2.53 (2.18-2.86) | 2.04 (1.67-2.4) |
| Australia | 1155 (1033-1227) | 6.79 (5.98-7.24) | 3608 (2927-3990) | 6.42 (5.24-7.08) | 0 (-0.18-0.17) |
| Austria | 659 (592-698) | 5.55 (4.94-5.9) | 1651 (1349-1818) | 6.75 (5.58-7.41) | 0.78 (0.47-1.09) |
| Azerbaijan | 59 (48-77) | 1.65 (1.34-2.14) | 124 (93-154) | 1.79 (1.39-2.21) | 0.76 (0.51-1) |
| Bahamas | 7 (6-7) | 5.56 (4.92-6.09) | 19 (16-22) | 6.06 (5.1-7.02) | 0.17 (-0.06-0.41) |
| Bahrain | 4 (2-5) | 6.97 (3.4-9.27) | 15 (8-20) | 5.66 (2.58-7.9) | -0.74 (-1.17--0.31) |
| Bangladesh | 1026 (567-1542) | 3.26 (1.75-4.9) | 4552 (3252-6400) | 5 (3.64-6.99) | 1.21 (0.83-1.59) |
| Barbados | 17 (15-18) | 5.84 (5.3-6.29) | 29 (24-34) | 5.74 (4.81-6.66) | 0.04 (-0.13-0.2) |
| Belarus | 415 (354-477) | 3.64 (3.08-4.18) | 677 (566-781) | 4.06 (3.41-4.69) | 0.17 (0.09-0.25) |
| Belgium | 694 (605-747) | 4.57 (3.93-4.93) | 1396 (1088-1557) | 4.21 (3.35-4.67) | 0.16 (-0.09-0.4) |
| Belize | 4 (3-4) | 3.96 (3.23-4.8) | 11 (9-12) | 4.61 (3.97-5.16) | 0.3 (-0.15-0.76) |
| Benin | 42 (29-54) | 3.09 (2.09-3.93) | 108 (82-137) | 3.66 (2.75-4.62) | 0.6 (0.55-0.66) |
| Bermuda | 3 (3-4) | 6.61 (5.63-7.4) | 7 (6-9) | 4.45 (3.69-5.36) | -1.47 (-1.55--1.39) |
| Bhutan | 3 (2-5) | 2.64 (1.42-4.22) | 22 (15-30) | 4.47 (3.09-6.28) | 1.89 (1.83-1.95) |
| Bolivia (Plurinational State of) | 112 (80-158) | 5.49 (4.04-7.63) | 358 (258-486) | 5.65 (4.13-7.54) | 0.22 (0.17-0.26) |
| Bosnia and Herzegovina | 91 (72-110) | 3.21 (2.53-3.9) | 258 (207-309) | 4.07 (3.26-4.87) | 0.91 (0.8-1.01) |
| Botswana | 8 (6-11) | 3.2 (2.25-4.46) | 26 (20-36) | 3.24 (2.42-4.69) | 0.4 (0.15-0.64) |
| Brazil | 2640 (2339-2805) | 5 (4.28-5.38) | 11250 (9412-12337) | 4.84 (4.04-5.31) | -0.15 (-0.24--0.06) |
| Brunei Darussalam | 5 (4-6) | 7.15 (5.28-9.27) | 12 (10-13) | 6.22 (5.18-7.43) | 0.22 (-0.04-0.48) |
| Bulgaria | 393 (346-449) | 5.51 (4.87-6.23) | 902 (786-1026) | 6.17 (5.4-6.98) | 0.5 (0.34-0.67) |
| Burkina Faso | 82 (48-116) | 3.78 (2.22-5.35) | 270 (181-365) | 5.13 (3.49-6.87) | 1.35 (1.2-1.49) |
| Burundi | 40 (18-74) | 2.73 (1.19-5.17) | 70 (34-135) | 2.74 (1.27-5.53) | -0.33 (-0.53--0.12) |
| Cabo Verde | 8 (6-11) | 3.62 (2.65-4.73) | 22 (16-28) | 5.43 (3.85-6.88) | 1.18 (0.93-1.43) |
| Cambodia | 74 (55-109) | 3.19 (2.31-4.7) | 279 (217-359) | 4.3 (3.27-5.61) | 0.99 (0.86-1.13) |
| Cameroon | 115 (81-146) | 4.99 (3.47-6.41) | 324 (245-422) | 5.21 (4-6.66) | 0.02 (-0.03-0.07) |
| Canada | 1498 (1339-1591) | 4.87 (4.33-5.18) | 3777 (3164-4143) | 4.29 (3.62-4.69) | -0.64 (-0.77--0.51) |
| Central African Republic | 22 (13-35) | 4.12 (2.33-6.56) | 41 (24-63) | 4.24 (2.44-6.54) | 0.11 (0.07-0.14) |
| Chad | 53 (31-78) | 2.95 (1.74-4.41) | 113 (77-151) | 3.82 (2.62-5.07) | 0.84 (0.79-0.89) |
| Chile | 254 (236-266) | 3.29 (3.01-3.47) | 995 (851-1075) | 3.73 (3.19-4.02) | 1.56 (1.04-2.09) |
| China | 16449 (13240-20521) | 4.93 (3.88-6.17) | 64728 (51765-77729) | 4.33 (3.43-5.23) | -0.6 (-0.78--0.43) |
| Colombia | 558 (506-587) | 4.5 (4.03-4.76) | 2331 (1893-2705) | 4.03 (3.31-4.67) | -0.64 (-0.75--0.53) |
| Comoros | 4 (2-5) | 3.42 (1.84-5.13) | 10 (6-17) | 3.34 (1.79-5.53) | -0.25 (-0.39--0.11) |
| Congo | 32 (24-47) | 5.93 (4.41-8.6) | 80 (56-101) | 5.97 (4.33-7.41) | -0.09 (-0.19-0.02) |
| Cook Islands | 1 (0-1) | 6.82 (5.38-8.29) | 1 (1-2) | 5.99 (4.2-7.74) | -0.39 (-0.45--0.33) |
| Costa Rica | 67 (59-73) | 4.4 (3.89-4.83) | 250 (207-282) | 4.3 (3.59-4.82) | -0.25 (-0.44--0.06) |
| Croatia | 142 (132-150) | 3.02 (2.79-3.19) | 363 (319-401) | 3.51 (3.09-3.87) | 0.62 (0.2-1.04) |
| Cuba | 442 (390-484) | 5.26 (4.58-5.78) | 1090 (943-1220) | 4.87 (4.22-5.46) | -0.22 (-0.34--0.09) |
| Cyprus | 59 (40-78) | 14.11 (9.57-19.11) | 123 (102-143) | 8.36 (6.79-9.85) | -1.76 (-2.04--1.49) |
| Czechia | 505 (473-532) | 4.08 (3.79-4.3) | 1123 (973-1237) | 4.68 (4.06-5.15) | 0.68 (0.57-0.79) |
| Côte d'Ivoire | 65 (49-83) | 4.26 (3.16-5.42) | 244 (188-307) | 4.71 (3.72-5.83) | 0.25 (0.18-0.31) |
| Democratic People's Republic of Korea | 421 (296-578) | 5.07 (3.43-7.31) | 1167 (886-1665) | 5.09 (3.77-7.73) | 0.32 (0.16-0.48) |
| Democratic Republic of the Congo | 281 (167-427) | 3.88 (2.25-5.95) | 854 (527-1390) | 4.45 (2.78-7.34) | 0.45 (0.23-0.67) |
| Denmark | 416 (373-453) | 4.73 (4.22-5.13) | 874 (741-955) | 6.06 (5.17-6.6) | 0.89 (0.44-1.34) |
| Djibouti | 2 (2-3) | 3.73 (2.64-5.16) | 10 (7-15) | 3.79 (2.49-5.46) | -0.06 (-0.17-0.04) |
| Dominica | 4 (3-5) | 7.7 (6.4-9.28) | 5 (5-6) | 7.79 (6.56-9.08) | -0.02 (-0.11-0.06) |
| Dominican Republic | 134 (110-156) | 6.01 (4.83-6.97) | 464 (361-587) | 4.96 (3.88-6.24) | -0.19 (-0.53-0.14) |
| Ecuador | 193 (171-209) | 5.06 (4.5-5.47) | 553 (464-656) | 4.01 (3.4-4.71) | -0.68 (-0.81--0.56) |
| Egypt | 459 (360-583) | 4.11 (3.16-5.32) | 971 (799-1145) | 3.61 (2.87-4.25) | -0.18 (-0.33--0.03) |
| El Salvador | 133 (110-157) | 4.8 (3.96-5.66) | 402 (307-494) | 5.36 (4.13-6.56) | 0.34 (0.24-0.45) |
| Equatorial Guinea | 5 (3-7) | 4.27 (2.66-6.69) | 15 (10-21) | 5.43 (3.59-7.37) | 0.83 (0.75-0.91) |
| Eritrea | 15 (9-22) | 3.14 (1.94-4.89) | 49 (26-86) | 3.94 (2.07-7.07) | 0.63 (0.57-0.69) |
| Estonia | 70 (64-77) | 3.95 (3.57-4.34) | 190 (161-212) | 5.33 (4.54-5.96) | 0.82 (0.71-0.93) |
| Eswatini | 6 (4-7) | 3.63 (2.61-4.89) | 11 (7-14) | 3.61 (2.59-4.61) | 0.67 (0.3-1.03) |
| Ethiopia | 250 (118-380) | 2.58 (1.21-3.96) | 697 (360-1128) | 2.52 (1.29-4.11) | -0.3 (-0.43--0.17) |
| Fiji | 10 (8-12) | 5.29 (4.48-6.39) | 26 (20-33) | 6.7 (5.37-8) | 0.52 (0.31-0.74) |
| Finland | 545 (466-595) | 7.77 (6.59-8.51) | 794 (645-877) | 4.67 (3.87-5.13) | -1.85 (-2.14--1.57) |
| France | 4923 (4382-5224) | 5.51 (4.88-5.85) | 9758 (8012-10779) | 4.53 (3.78-4.99) | -0.55 (-0.61--0.48) |
| Gabon | 25 (19-35) | 6.35 (4.65-8.87) | 41 (31-50) | 6.94 (5.29-8.37) | 0.13 (0.02-0.24) |
| Gambia | 6 (4-9) | 3.86 (2.69-5.24) | 29 (22-36) | 5.11 (3.91-6.43) | 0.92 (0.88-0.95) |
| Georgia | 168 (126-202) | 3.26 (2.37-3.99) | 341 (293-380) | 5.05 (4.37-5.62) | 1.43 (0.52-2.34) |
| Germany | 8302 (7144-9077) | 6.39 (5.46-6.99) | 20539 (16509-22874) | 7.81 (6.35-8.66) | 1.17 (0.96-1.37) |
| Ghana | 128 (101-155) | 4.33 (3.42-5.3) | 346 (267-424) | 4.26 (3.27-5.27) | -0.4 (-0.55--0.25) |
| Greece | 590 (529-621) | 4.51 (4-4.77) | 1527 (1272-1673) | 4.25 (3.6-4.62) | -0.3 (-0.36--0.24) |
| Greenland | 2 (1-2) | 9.44 (7.45-10.9) | 3 (2-4) | 7.37 (5.55-9.35) | -0.51 (-0.64--0.38) |
| Grenada | 5 (4-5) | 5.35 (4.48-6.2) | 5 (5-6) | 7.08 (6.12-7.69) | 0.82 (0.47-1.17) |
| Guam | 2 (2-3) | 7.3 (6.23-8.47) | 5 (4-6) | 2.08 (1.64-2.41) | -3 (-3.56--2.45) |
| Guatemala | 84 (78-92) | 5.44 (4.77-6.18) | 358 (309-404) | 4.32 (3.74-4.84) | -0.71 (-0.88--0.54) |
| Guinea | 75 (45-109) | 3.53 (2.08-5.15) | 153 (110-198) | 4.33 (3.15-5.56) | 0.71 (0.65-0.76) |
| Guinea-Bissau | 8 (6-11) | 4.3 (3.11-6.14) | 14 (11-19) | 5.1 (3.79-6.68) | 0.61 (0.56-0.65) |
| Guyana | 12 (11-13) | 4.65 (4.16-5.09) | 24 (20-29) | 5.58 (4.58-6.68) | 0.61 (0.24-0.99) |
| Haiti | 97 (64-153) | 5.81 (3.86-9.01) | 219 (147-326) | 5.63 (3.8-8.29) | -0.04 (-0.09-0.02) |
| Honduras | 56 (40-75) | 4.29 (3.05-6.07) | 302 (230-372) | 7.51 (5.71-9.15) | 1.8 (1.47-2.14) |
| Hungary | 528 (494-563) | 4.34 (4.02-4.63) | 796 (678-894) | 3.55 (3.03-3.99) | -0.38 (-0.52--0.25) |
| Iceland | 18 (15-19) | 5.62 (4.88-6.04) | 51 (40-58) | 6.84 (5.49-7.68) | 1.05 (0.86-1.23) |
| India | 5413 (3291-8480) | 2.15 (1.32-3.41) | 27888 (20426-34928) | 3.44 (2.5-4.32) | 1.84 (1.53-2.16) |
| Indonesia | 1899 (1376-2468) | 3.6 (2.54-4.93) | 6987 (5445-8421) | 6.36 (4.84-7.8) | 1.84 (1.72-1.96) |
| Iran (Islamic Republic of) | 411 (306-510) | 3.32 (2.42-4.15) | 1949 (1463-2233) | 3.31 (2.47-3.8) | -0.07 (-0.15-0.02) |
| Iraq | 226 (161-304) | 3.25 (2.31-4.38) | 647 (481-826) | 4.85 (3.51-6.19) | 0.79 (0.58-1.01) |
| Ireland | 220 (200-231) | 6.07 (5.45-6.42) | 456 (371-510) | 5.2 (4.23-5.8) | -0.25 (-0.49-0) |
| Israel | 256 (228-273) | 6.31 (5.5-6.76) | 714 (580-794) | 4.84 (3.98-5.35) | -0.66 (-0.85--0.48) |
| Italy | 3323 (2852-3560) | 4.09 (3.47-4.41) | 9825 (7691-11040) | 4.52 (3.6-5.05) | 0.76 (0.54-0.97) |
| Jamaica | 95 (85-105) | 5.17 (4.6-5.7) | 206 (164-251) | 5.46 (4.36-6.68) | 0.19 (-0.01-0.4) |
| Japan | 4429 (3883-4699) | 3.04 (2.63-3.25) | 13837 (10679-15687) | 2.33 (1.86-2.58) | -1.58 (-1.98--1.17) |
| Jordan | 23 (19-28) | 3.14 (2.54-3.78) | 103 (80-125) | 2.72 (2.12-3.29) | -0.56 (-0.79--0.33) |
| Kazakhstan | 213 (172-265) | 2.2 (1.75-2.79) | 350 (305-395) | 2.97 (2.57-3.34) | 0.49 (0.24-0.75) |
| Kenya | 122 (74-167) | 2.43 (1.48-3.32) | 407 (264-581) | 3.39 (2.18-4.95) | 1.22 (1.1-1.34) |
| Kiribati | 1 (1-1) | 3.19 (2.42-4.37) | 1 (1-2) | 3.86 (3.02-5.05) | 0.55 (0.45-0.65) |
| Kuwait | 9 (7-10) | 2.59 (2.08-2.95) | 60 (48-72) | 3 (2.4-3.57) | 0.82 (0.26-1.39) |
| Kyrgyzstan | 46 (38-56) | 2.01 (1.65-2.47) | 73 (61-84) | 2.17 (1.82-2.49) | 0.37 (0.12-0.63) |
| Lao People's Democratic Republic | 41 (27-60) | 4.19 (2.82-6.25) | 130 (102-169) | 5.14 (4.04-6.72) | 0.6 (0.57-0.64) |
| Latvia | 122 (112-129) | 3.74 (3.44-3.97) | 226 (194-250) | 4.49 (3.88-4.98) | 0.8 (0.59-1.02) |
| Lebanon | 73 (41-113) | 4.96 (2.67-7.76) | 239 (193-308) | 3.41 (2.75-4.35) | -1.46 (-1.61--1.32) |
| Lesotho | 14 (10-20) | 2.42 (1.67-3.51) | 22 (16-29) | 3.53 (2.63-4.59) | 2.28 (1.85-2.71) |
| Liberia | 26 (17-33) | 4.12 (2.69-5.34) | 50 (36-67) | 4.52 (3.28-5.97) | 0.35 (0.29-0.4) |
| Libya | 38 (27-53) | 2.48 (1.79-3.43) | 111 (69-163) | 3.07 (1.9-4.49) | 1.43 (1.15-1.71) |
| Lithuania | 154 (138-170) | 3.67 (3.3-4.04) | 328 (288-363) | 4.52 (3.98-5.01) | 0.73 (0.62-0.84) |
| Luxembourg | 33 (31-35) | 6.64 (6.11-7.02) | 83 (70-92) | 6.32 (5.38-7.03) | 0.24 (0.07-0.41) |
| Madagascar | 153 (98-205) | 4.9 (3.08-6.61) | 271 (177-388) | 5.12 (3.35-7.35) | 0.14 (0.02-0.26) |
| Malawi | 48 (25-70) | 2.42 (1.22-3.61) | 122 (77-179) | 2.94 (1.86-4.29) | 0.58 (0.48-0.69) |
| Malaysia | 237 (193-289) | 3.22 (2.6-3.94) | 931 (786-1069) | 4.94 (4.12-5.74) | 1.54 (1.14-1.94) |
| Maldives | 1 (1-2) | 4.05 (2.57-5.55) | 10 (7-12) | 4.06 (2.99-5.05) | -0.22 (-0.37--0.06) |
| Mali | 52 (34-75) | 2.84 (1.81-4.14) | 128 (90-170) | 2.95 (2.12-3.84) | 0.24 (0.11-0.37) |
| Malta | 19 (17-20) | 5.28 (4.67-5.65) | 58 (47-66) | 4.88 (3.97-5.49) | -0.02 (-0.18-0.14) |
| Marshall Islands | 1 (0-1) | 6.9 (5.34-9.13) | 1 (1-2) | 7.35 (5.53-9.4) | 0.1 (0.03-0.16) |
| Mauritania | 28 (22-35) | 4.85 (3.79-5.96) | 73 (54-96) | 5.36 (4.02-7.01) | 0.12 (0-0.24) |
| Mauritius | 23 (21-24) | 5.36 (4.79-5.74) | 76 (66-82) | 4.84 (4.24-5.26) | -0.42 (-0.58--0.27) |
| Mexico | 1500 (1417-1547) | 5.69 (5.32-5.89) | 5147 (4611-5659) | 4.86 (4.33-5.33) | -0.44 (-0.53--0.36) |
| Micronesia (Federated States of) | 2 (2-3) | 8.31 (5.76-11.84) | 3 (2-4) | 8.86 (6.48-11.44) | 0.16 (0.14-0.18) |
| Monaco | 4 (3-5) | 4.34 (3.2-5.36) | 6 (4-7) | 4.35 (3.29-5.49) | 0.11 (-0.1-0.31) |
| Mongolia | 15 (12-19) | 2 (1.54-2.61) | 26 (20-33) | 1.91 (1.49-2.44) | -0.59 (-0.74--0.43) |
| Montenegro | 61 (52-78) | 11.32 (9.48-14.4) | 133 (107-163) | 17.26 (13.44-21.44) | 1.7 (1.23-2.18) |
| Morocco | 304 (183-417) | 3.04 (1.79-4.21) | 991 (711-1280) | 4.28 (3.05-5.49) | 1.2 (1.05-1.35) |
| Mozambique | 104 (56-152) | 3.3 (1.83-4.93) | 252 (156-395) | 4.34 (2.74-6.93) | 1.25 (1.12-1.39) |
| Myanmar | 504 (350-722) | 4.16 (2.93-5.94) | 1630 (1236-2084) | 5.15 (3.9-6.6) | 0.46 (0.36-0.57) |
| Namibia | 10 (7-13) | 3.08 (2.29-4.14) | 29 (21-40) | 3.77 (2.68-5.09) | 0.66 (0.46-0.85) |
| Nauru | 0 (0-0) | 8.16 (6.34-10.16) | 0 (0-0) | 10.35 (6.38-18.44) | 0.82 (0.79-0.84) |
| Nepal | 116 (63-184) | 2.31 (1.25-3.65) | 589 (401-829) | 4.05 (2.76-5.8) | 2 (1.72-2.28) |
| Netherlands | 1399 (1213-1507) | 6.99 (6.03-7.56) | 2501 (2084-2738) | 6.01 (5.03-6.57) | -0.72 (-0.86--0.59) |
| New Zealand | 281 (246-300) | 8.01 (6.94-8.6) | 729 (593-805) | 7.52 (6.14-8.3) | 0.04 (-0.1-0.17) |
| Nicaragua | 39 (32-46) | 3.45 (2.84-4.13) | 137 (111-165) | 3.7 (3-4.48) | 0.57 (0.31-0.82) |
| Niger | 32 (17-49) | 2.77 (1.43-4.25) | 114 (57-174) | 3.07 (1.61-4.71) | 0.36 (0.3-0.42) |
| Nigeria | 1258 (955-1655) | 5.1 (3.87-6.71) | 2185 (1644-2671) | 4.48 (3.44-5.46) | -0.74 (-0.89--0.59) |
| Niue | 0 (0-0) | 7.28 (5.81-8.89) | 0 (0-0) | 7.39 (5.57-8.79) | -0.07 (-0.11--0.03) |
| North Macedonia | 64 (54-77) | 4.41 (3.68-5.34) | 149 (110-214) | 7.45 (5.8-9.5) | 1.47 (0.87-2.08) |
| Northern Mariana Islands | 0 (0-1) | 6.73 (5.51-8.36) | 2 (2-3) | 7.7 (6.44-8.85) | 0.36 (0.06-0.65) |
| Norway | 491 (431-522) | 6.27 (5.51-6.68) | 778 (629-857) | 5.94 (4.86-6.51) | -0.25 (-0.51-0.01) |
| Oman | 17 (12-23) | 4.03 (2.91-5.59) | 45 (36-55) | 4.73 (3.62-5.93) | 1.06 (0.62-1.5) |
| Pakistan | 1035 (660-1504) | 2.8 (1.81-4.06) | 3115 (2311-4332) | 4.58 (3.33-6.38) | 1.41 (1.14-1.67) |
| Palau | 0 (0-0) | 4.84 (3.86-6.01) | 1 (0-1) | 4.69 (3.77-5.59) | 0.08 (-0.02-0.18) |
| Palestine | 29 (21-37) | 5.11 (3.74-6.68) | 68 (55-82) | 5.37 (4.31-6.66) | 0 (-0.17-0.18) |
| Panama | 53 (47-57) | 4.23 (3.73-4.57) | 221 (170-263) | 4.59 (3.55-5.46) | 0.34 (0.23-0.45) |
| Papua New Guinea | 32 (19-46) | 3.46 (2.1-4.84) | 108 (67-154) | 3.89 (2.43-5.52) | 0.38 (0.35-0.41) |
| Paraguay | 88 (71-102) | 4.78 (3.89-5.6) | 290 (218-360) | 5.73 (4.31-7.1) | 0.9 (0.74-1.06) |
| Peru | 435 (364-513) | 4.33 (3.61-5.09) | 1169 (896-1501) | 3.47 (2.66-4.45) | -1.03 (-1.34--0.73) |
| Philippines | 691 (595-803) | 5.26 (4.52-6.12) | 2445 (2054-2912) | 4.82 (4.02-5.81) | 0.16 (0-0.32) |
| Poland | 2503 (2303-2671) | 6.8 (6.18-7.24) | 4021 (3480-4421) | 4.96 (4.31-5.45) | -0.67 (-1.18--0.16) |
| Portugal | 507 (462-531) | 4.69 (4.21-4.93) | 1173 (969-1288) | 3.46 (2.9-3.78) | -1.17 (-1.36--0.99) |
| Puerto Rico | 170 (156-180) | 5.97 (5.48-6.31) | 390 (313-452) | 3.82 (3.11-4.43) | -1.6 (-1.72--1.47) |
| Qatar | 2 (1-3) | 7.37 (3.17-10.02) | 10 (5-15) | 4.06 (1.45-6.04) | -2.71 (-3.39--2.04) |
| Republic of Korea | 553 (422-873) | 3.38 (2.55-5.22) | 3165 (2356-3785) | 3.5 (2.6-4.17) | 0.61 (0.41-0.81) |
| Republic of Moldova | 108 (99-116) | 3.96 (3.65-4.24) | 215 (189-238) | 3.5 (3.09-3.88) | -0.63 (-0.8--0.46) |
| Romania | 674 (625-726) | 3.58 (3.33-3.83) | 1429 (1263-1586) | 3.35 (2.97-3.72) | -0.56 (-0.74--0.39) |
| Russian Federation | 5109 (4515-5816) | 3.92 (3.42-4.52) | 10825 (9597-11704) | 4.44 (3.92-4.8) | 0.38 (0.25-0.51) |
| Rwanda | 56 (27-84) | 3.74 (1.71-5.7) | 102 (42-179) | 3.03 (1.24-5.39) | -1.31 (-1.62--1) |
| Saint Kitts and Nevis | 2 (2-3) | 8.38 (7.51-9.3) | 3 (3-3) | 7.65 (6.77-8.39) | -0.05 (-0.26-0.17) |
| Saint Lucia | 6 (5-6) | 10.41 (9.62-11.35) | 16 (13-19) | 7.2 (5.98-8.44) | -2 (-2.33--1.68) |
| Saint Vincent and the Grenadines | 4 (4-5) | 7.93 (7.37-8.39) | 8 (7-9) | 7.43 (6.63-8.15) | -0.18 (-0.39-0.03) |
| Samoa | 4 (3-5) | 6.77 (4.91-8.9) | 7 (5-9) | 6.72 (5.11-8.46) | -0.05 (-0.14-0.03) |
| San Marino | 2 (2-3) | 5.93 (4.79-7) | 4 (2-5) | 3.03 (2.03-4.31) | -1.3 (-1.69--0.91) |
| Sao Tome and Principe | 2 (1-2) | 3.49 (2.42-5.7) | 3 (2-4) | 4.94 (3.79-5.87) | 1.42 (1.28-1.55) |
| Saudi Arabia | 102 (78-142) | 2.97 (2.27-4.12) | 249 (197-305) | 3.35 (2.67-4.11) | 0.3 (0.2-0.4) |
| Senegal | 72 (49-97) | 3.89 (2.64-5.32) | 232 (173-291) | 5.03 (3.76-6.29) | 0.67 (0.6-0.75) |
| Serbia | 427 (363-489) | 6.22 (5.28-7.24) | 810 (665-962) | 4.63 (3.83-5.5) | -1.49 (-1.84--1.13) |
| Seychelles | 3 (2-3) | 5.03 (4.27-6.17) | 5 (4-6) | 5.46 (4.33-6.73) | 0.47 (0.29-0.66) |
| Sierra Leone | 45 (30-62) | 3.46 (2.27-4.78) | 82 (61-106) | 3.86 (2.96-4.89) | 0.38 (0.33-0.43) |
| Singapore | 32 (29-33) | 1.94 (1.78-2.04) | 99 (84-108) | 1.22 (1.03-1.33) | -1.82 (-2.05--1.59) |
| Slovakia | 305 (263-366) | 5.75 (4.94-6.89) | 562 (463-658) | 5.99 (4.9-7.02) | 0.39 (0.28-0.5) |
| Slovenia | 83 (76-89) | 3.54 (3.22-3.83) | 214 (175-242) | 3.67 (3.01-4.15) | 0.46 (0.03-0.88) |
| Solomon Islands | 3 (1-4) | 4.09 (2.11-6.13) | 9 (5-13) | 4.92 (2.65-8.18) | 0.58 (0.56-0.6) |
| Somalia | 32 (15-51) | 2.79 (1.29-4.5) | 61 (26-111) | 2.23 (1.02-3.98) | -0.56 (-0.64--0.48) |
| South Africa | 411 (328-515) | 2.77 (2.2-3.48) | 1208 (1035-1329) | 4.04 (3.43-4.51) | 1.16 (0.82-1.51) |
| South Sudan | 54 (25-82) | 3.32 (1.57-4.96) | 63 (31-99) | 2.97 (1.47-4.74) | -0.4 (-0.52--0.28) |
| Spain | 2611 (2310-2779) | 5.21 (4.56-5.56) | 6767 (5396-7550) | 4.54 (3.69-5.04) | -0.35 (-0.44--0.26) |
| Sri Lanka | 209 (180-246) | 3.64 (3.07-4.33) | 777 (556-1029) | 4.05 (2.89-5.35) | 1.03 (0.74-1.31) |
| Sudan | 155 (98-232) | 2.87 (1.76-4.2) | 394 (287-519) | 3.24 (2.32-4.25) | 0.32 (0.21-0.42) |
| Suriname | 12 (10-14) | 5.52 (4.6-6.35) | 29 (21-37) | 5.28 (3.89-6.83) | 0.11 (-0.04-0.26) |
| Sweden | 839 (737-898) | 4.9 (4.29-5.25) | 2851 (2296-3193) | 9.47 (7.71-10.59) | 2.57 (2.28-2.87) |
| Switzerland | 317 (271-346) | 2.74 (2.35-3.01) | 773 (609-867) | 3 (2.39-3.34) | 0.62 (0.5-0.75) |
| Syrian Arab Republic | 153 (109-201) | 4.26 (3.02-5.62) | 318 (210-419) | 4.57 (2.89-5.92) | -0.11 (-0.31-0.09) |
| Taiwan (Province of China) | 403 (369-425) | 5.23 (4.64-5.59) | 1771 (1474-1977) | 3.71 (3.12-4.12) | -1.36 (-1.67--1.04) |
| Tajikistan | 28 (20-41) | 1.26 (0.9-1.9) | 39 (32-48) | 1.15 (0.92-1.44) | -0.61 (-0.96--0.26) |
| Thailand | 1206 (930-1572) | 5.81 (4.39-7.57) | 5064 (3744-6440) | 4.56 (3.39-5.83) | -1.35 (-1.56--1.15) |
| Timor-Leste | 5 (3-7) | 3.56 (2.28-5.09) | 22 (17-28) | 4.54 (3.47-5.8) | 0.89 (0.77-1.01) |
| Togo | 24 (17-31) | 3.83 (2.84-5.08) | 77 (57-98) | 4.7 (3.6-5.93) | 0.6 (0.57-0.63) |
| Tokelau | 0 (0-0) | 7.36 (5.74-9.22) | 0 (0-0) | 7.16 (4.95-10.39) | -0.06 (-0.08--0.03) |
| Tonga | 2 (1-2) | 4.41 (3-6.32) | 4 (3-5) | 5.01 (3.51-6.87) | 0.48 (0.36-0.6) |
| Trinidad and Tobago | 38 (35-40) | 7.53 (6.81-8.15) | 99 (77-119) | 5.64 (4.41-6.79) | -0.79 (-0.92--0.66) |
| Tunisia | 94 (69-117) | 3.62 (2.6-4.59) | 463 (305-649) | 4.61 (2.98-6.69) | 0.6 (0.52-0.68) |
| Turkmenistan | 29 (24-34) | 2.19 (1.83-2.65) | 86 (68-112) | 2.89 (2.27-3.76) | 0.5 (0.29-0.72) |
| Tuvalu | 0 (0-0) | 6.98 (5.26-9.23) | 0 (0-1) | 7.14 (5.55-8.84) | 0.02 (-0.03-0.06) |
| Türkiye | 836 (659-1100) | 3.59 (2.79-4.75) | 2923 (2332-3533) | 3.88 (3.05-4.69) | 0.75 (0.17-1.33) |
| Uganda | 103 (44-165) | 2.69 (1.13-4.35) | 251 (126-429) | 2.91 (1.43-4.96) | 0.15 (0.02-0.28) |
| Ukraine | 2156 (1971-2362) | 3.85 (3.47-4.24) | 3236 (2489-4051) | 4.1 (3.16-5.13) | -0.21 (-0.44-0.03) |
| United Arab Emirates | 7 (5-10) | 3.62 (2.64-5.17) | 30 (24-37) | 3.82 (2.51-4.93) | 2.97 (1.9-4.04) |
| United Kingdom | 5083 (4544-5338) | 5.47 (4.84-5.77) | 9412 (7868-10176) | 5.71 (4.8-6.15) | 0.3 (0.17-0.43) |
| United Republic of Tanzania | 216 (115-327) | 3.61 (1.91-5.49) | 580 (329-891) | 3.6 (2.05-5.39) | -0.16 (-0.36-0.04) |
| United States of America | 13044 (11221-13964) | 3.88 (3.33-4.16) | 35285 (28993-38677) | 5.26 (4.36-5.74) | 0.95 (0.88-1.02) |
| United States Virgin Islands | 4 (3-4) | 7.66 (6.41-8.8) | 8 (6-10) | 5.15 (3.97-6.7) | -1.04 (-1.26--0.82) |
| Uruguay | 117 (105-126) | 3.23 (2.87-3.49) | 277 (235-301) | 3.78 (3.24-4.1) | 0.71 (0.58-0.84) |
| Uzbekistan | 97 (57-158) | 1.03 (0.6-1.69) | 240 (204-278) | 1.39 (1.18-1.6) | 1.26 (1-1.52) |
| Vanuatu | 1 (1-2) | 4.62 (3.07-6.47) | 5 (3-6) | 5.14 (3.8-6.54) | 0.35 (0.29-0.41) |
| Venezuela (Bolivarian Republic of) | 327 (292-355) | 4.65 (4.08-5.09) | 1204 (948-1481) | 4.58 (3.63-5.6) | -0.14 (-0.29-0.01) |
| Viet Nam | 1219 (961-1630) | 4.14 (3.22-5.56) | 4015 (2990-5030) | 5.83 (4.35-7.36) | 1.06 (0.97-1.15) |
| Yemen | 76 (44-121) | 3.08 (1.79-4.86) | 291 (198-404) | 3.74 (2.57-5.12) | 0.6 (0.55-0.65) |
| Zambia | 64 (44-81) | 4.11 (2.84-5.3) | 218 (129-366) | 5.71 (3.59-9.09) | 1.25 (0.95-1.55) |
| Zimbabwe | 63 (50-76) | 3.08 (2.34-3.75) | 118 (80-149) | 3.39 (2.23-4.25) | 0.81 (0.5-1.13) |

ASDR=Age-standardized deaths rate, CI=Confidence interval, EAPC=Estimated annual percentage change, UI=Uncertainty interval
